# Selective NH_3_-to-N_2_H_4_ conversion electrocatalysed by ruthenium(ii)-cymene complexes

**DOI:** 10.1039/d5sc08826g

**Published:** 2026-01-13

**Authors:** Xi Zhang, Shan Zhao, Chen Zhou, Guo Chen, Liru Cao, Jian Lin, Chen Tang, Zhi-Yan Liu, Piao He, Xiao-Yi Yi

**Affiliations:** a College of Chemistry and Chemical Engineering, Central South University Changsha Hunan 410083 P. R. China xyyi@csu.edu.cn; b School of Chemistry and Chemical Engineering, Southwest University Chongqing 400715 P. R. China; c CAS Key Laboratory of Science and Technology on Applied Catalysis, Dalian Institute of Chemical Physics, Chinese Academy of Sciences Dalian 116023 P. R. China

## Abstract

A series of ruthenium(ii)-cymene complexes [(*η*^6^-*p*-cymene)Ru(pp)Cl] (1–4) and corresponding NH_3_-ligated complexes [(*η*^6^-*p*-cymene)Ru(pp)(NH_3_)]PF_6_ ([1-NH_3_]PF_6_ to [4-NH_3_]PF_6_), where cymene = 4-isopropyltoluene and pp^−^ = pyridylpyrrole ligand, have been designed and synthesized. Structural modifications of pp^−^ ligands are accomplished through the use of an increasing number of electron-donating methyl groups on the pyrrole unit. Solid-state structural analysis shows that these complexes have a typical piano-stool structure. Electrochemical studies of these complexes illustrate that the introduction of a methyl group to the pp^−^ ligand can greatly decrease the oxidation potential of Ru^iii/ii^ from 0.49 V *vs.* Cp_2_Fe^+/0^ for [1-NH_3_]PF_6_ to 0.16 V *vs.* Cp_2_Fe^+/0^ for [4-NH_3_]PF_6_. Controlled potential coulometry experiments show that these complexes exhibit selective catalysis for the oxidation of NH_3_ to N_2_H_4_ with a turnover number of up to 453.2 at *E*_app_ 0.8 V *vs.* Cp_2_Fe^+/0^ for the [4-NH_3_]PF_6_ complex. Kinetic and theoretical thermodynamic studies show that the pathway of bimolecular coupling of Ru^ii^-aminyl species and the pathway of ammonia nucleophilic attack of Ru^iv^-imide (generated from the disproportionation of Ru^iii^-amide) are involved in N–N formation.

## Abstract 2

NH_3_-ligated complexes [(*η*^6^-*p*-cymene)Ru(pp)(NH_3_)]PF_6_ display good catalytic performance for selective conversion NH_3_ to N_2_H_4_.

## Graphical abstract

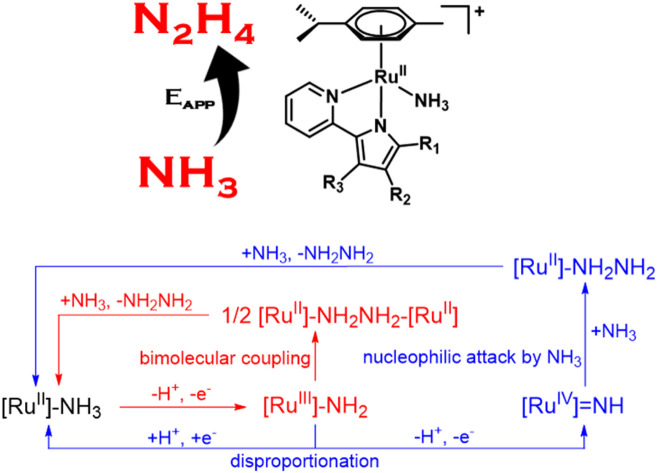

To meet the challenges of a large-scale energy crisis and environmental pollution, energy-rich H_2_ of a green and sustainable nature has attracted much interest as an alternative energy source, although storage and distribution of liquid H_2_ still suffer from harsh conditions along with a lack of infrastructure. Ammonia (NH_3_) is a good candidate as a hydrogen energy carrier, and offers approximately 1.7 times the energy density of liquid H_2_.^1–4^ The NH_3_ industry has not only seen applications worldwide but also has widespread facilities for storage, transport and handing. The classical catalytic cracking reaction for NH_3_-to-H_2_ conversion (eqn (1)) requires a precious metal catalyst and high temperature, which are of relatively high cost. Moreover, the generated N_2_ product is directly discharged into the air, resulting in low atomic utilization and relatively poor economy in NH_3_-to-H_2_ conversion.

1 2NH_3_(g) → N_2_(g) + 3H_2_(g) Δ*G*^θ^ = 7.9 kcal mol^−1^

2 2NH_3_(g) → N_2_H_4_(l) + H_2_(g) Δ*G*^θ^ = 43.6 kcal mol^−1^

3 N_2_H_4_(l) → N_2_(g) + 2H_2_(g) Δ*G*^θ^ = −35.7 kcal mol^−1^

Selective electrocatalytic conversion of NH_3_ into N_2_H_4_ and H_2_ (NH_3_-to-N_2_H_4_ conversion, eqn (2)) seems more appealing than NH_3_-to-H_2_ conversion due to the advantages of not only generating H_2_, but also simultaneously producing high value-added N_2_H_4_ (the price of anhydrous hydrazine is about 58 000 USD t^−1^). However, this route is a thermodynamically demanding process (Δ*G*^θ^ = 43.6 kcal mol^−1^), and needs to overcome the competitive reaction of spontaneous dehydrogenation of N_2_H_4_ to N_2_ (eqn (3), Δ*G*^θ^ = −35.7 kcal mol^−1^). Hence, highly efficient and selective NH_3_-to-N_2_H_4_ conversion is appealing, but remains a huge scientific challenge.^5^

Molecular catalysts can offer several advantages over their heterogeneous counterparts, such as controllable structure, convenient characterization, and well-defined active site nature, which allow for mechanistic studies to elucidate the factors controlling the catalytic activity and selectivity. Since the seminal work by Hamann and Smith III on the electro-oxidation of NH_3_ to N_2_ catalyzed by [(trpy)(bpy^NMe2^)Ru(NH_3_)](PF_6_)_2_ (trpy = 2,2′:6′,2″-terpyridine, bpy^NMe2^ = 4,4′-bis(dimethylamino)-2,2′-bipyridine),^6^ significant progress has been made in the development of molecular catalysts for ammonia oxidation.^7–22^ Nevertheless, the reported catalytic systems are mostly concerned with the oxidation of NH_3_ to N_2_, and there are few reports on the selective catalytic conversion of NH_3_ into N_2_H_4_. In 2023, we demonstrated that [Ru(*κ*^2^-*N*,*N*′-dpp)(bpy)(dmso)(NH_3_)]PF_6_ (Hdpp = 2,5-di(pyridin-2-yl)-1H-pyrrole)^15*a*^ can catalyze the electrocatalytic conversion of NH_3_ into N_2_H_4_ with unprecedentedly high selectivity (over 97.9%) and turnover frequency (238.9 h^−1^). Although a similar ligated-N_2_H_4_ intermediate and a similar N–N formation pathway—such as either bimolecular coupling of a metal-imide^15,18,19,20*a*^ or ammonia nucleophilic attack of a metal-imide^6,10,17,22^—are involved, this catalyst is in sharp contrast with conventional ones that usually generate N_2_ as the major N–N coupling product with relatively low turnover frequencies. N_2_H_4_/N_2_ selectivity is usually represented by a branch from a ligated-N_2_H_4_ intermediate,^14,15*a*^ which could directly release N_2_H_4_ or continue to be over-oxidized to release N_2_. The release of N_2_H_4_ from a ligated-N_2_H_4_ intermediate to restart the catalytic cycle is one of the key issues for the selective oxidation of NH_3_ to N_2_H_4_. Thus, rational design of the ancillary ligand backbone is still a desirable strategy for developing highly efficient and selective catalysts for NH_3_-to-N_2_H_4_ conversion.

We have long been interested in the study of metal complexes based on the pyridylpyrrole (pp^−^) ligand, which is structurally analogous to bipyridine.^23^ Notwithstanding the similar structure and coordination properties, the π-donation from the pyrrolyl group in the pp^−^ ligand increases the energy of the metal-based LUMO in the M–N_2_H_4_ intermediate, thus weakening the M–N_2_H_4_ bond and facilitating the release of N_2_H_4_.^24^ In addition, the negative charge of the pyrrolyl unit not only lowers the overpotential of the metal complex but also reduces the overall positive charge of the reactive intermediate, which is a major cause of instability in the intermediates during the AO catalytic cycle.^23^ Herein, we design an ancillary pp^−^ ligand using an increasing number of electron-donating methyl groups on the pyrrole unit to regulate the electronic structure of the metal complex. Corresponding ruthenium(ii)-cymene complexes [(*η*^6^-*p*-cymene)Ru(pp)Cl] (where pp^−^ is HL1 = 2-(1*H*-pyrrol-2-yl)pyridine (1), HL2 = 2-(4-methyl-1*H*-pyrrol-2-yl)pyridine (2), HL3 = 2-(3,5-dimethyl-1*H*-pyrrol-2-yl)pyridine (3), and HL4 = 2-(3,4,5-trimethyl-1*H*-pyrrol-2-yl)pyridine (4)) and corresponding NH_3_-ligated complexes [(*η*^6^-*p*-cymene)Ru(pp)(NH_3_)]PF_6_ ([1-NH_3_]PF_6_ to [4-NH_3_]PF_6_) are reported. Their selective catalysis for NH_3_-to-N_2_H_4_ conversion and the catalytic mechanism are also presented.

As shown in Fig. 1a, complexes 1–4 are synthesized by treatment of dimeric precursor of [(*η*^6^-*p*-cymene)RuCl_2_]_2_ and a deprotonated pyridylpyrrole ligand in CH_2_Cl_2_ at 0 °C in moderate yield (∼50%). The corresponding NH_3_-ligated complexes [(*η*^6^-*p*-cymene)Ru(pp)(NH_3_)]PF_6_ ([1-NH_3_]PF_6_ to [4-NH_3_]PF_6_) are synthesized in over 60% yield by treatment of 1–4 with one equiv. of AgPF_6_ in CH_3_CN followed by bubbling NH_3_ gas. These complexes are stable in common organic solvents, and are fully characterized by ESI-MS, NMR and IR spectroscopy (Fig. S6–S29). Compared to 1–4, the ^1^H NMR spectra of [1-NH_3_]PF_6_ to [4-NH_3_]PF_6_ show a newly added broad single peak at ∼2.0 ppm due to the ligated-NH_3_. Similarly, the IR spectra of [1-NH_3_]PF_6_ to [4-NH_3_]PF_6_ show an additional band at ∼3340–3350 cm^−1^ due to N–H stretching, also indicating that NH_3_ binds to the Ru center. The solid-state structures of 1–4 and [2-NH_3_]PF_6_ are shown in Fig. 1b. The crystallographic data and selected bond distances and angles are listed in Tables S1–S6. These complexes exhibit a typical piano-stool structure with one pp^−^, one cymene and one Cl^−^ (or NH_3_) ligand coordinating to the ruthenium center. The bond distance of Ru–N_pyrrole_ (2.042(10)–2.074(3) Å) is slightly shorter than that of Ru–N_pyridine_ (2.101(2)–2.113(3) Å), mainly due to the electrostatic interaction between the Ru atom and the anionic pyrrolide N atom. In [2-NH_3_]PF_6_, NH_3_ coordinates to the Ru center with a Ru–N bond distance of 2.138(12) Å, which is similar to that of other NH_3_-ligated Ru(ii) complexes.

**Fig. 1:**
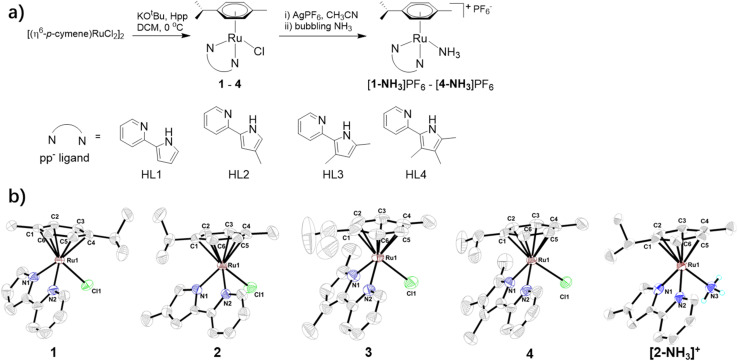
(a) The synthetic routes of 1–4, and [1-NH_3_]PF_6_ to [4-NH_3_]PF_6_. (b) Solid-state structures of complexes 1–4 and [2-NH_3_]^+^. The hydrogen atoms on cymene, the pp^−^ ligand and anionic PF_6_^−^ in [2-NH_3_]PF_6_ are omitted for clarity.

The electrochemical behavior of the title complexes in CH_3_CN is investigated by cyclic voltammetry (CV) and differential pulse voltammetry (DPV) using a AgCl/Ag electrode in a saturated KCl solution as a reference electrode. Unless otherwise specified, all potentials are converted into *E*_1/2_*vs.* Cp_2_Fe^+/0^ in CH_3_CN by adding −0.43 V to the measured potential.

The electrochemical behavior of 1–4 is shown in Fig. 2a. The first oxidation wave is assigned to ruthenium center oxidation (Ru^ii^ → Ru^iii^). The electro-donating nature of the pp^−^ ligand significantly influences the redox potential of these complexes, leading to a decreasing Ru^iii/ii^ reduction potential from 0.37 V for 1 to 0.04 V for 4 with an increase in the number of electron-donating methyl groups on the pyrrole unit. The redox potentials of the second oxidation wave at ∼1.2 V and third oxidation wave at ∼1.6 V (see the DPV curves in Fig. 2a) are independent of the methyl substituted pp^−^ ligand. These could clearly be assigned to Cl^−^ and cymene ligand oxidation events, respectively.^25,26^Fig. 2b shows that [1-NH_3_]PF_6_ to [4-NH_3_]PF_6_ exhibit two oxidation waves. The first one (0.49 V, 0.36 V, 0.23 V and 0.16 V for [1-NH_3_]PF_6_ to [4-NH_3_]PF_6_, respectively) is attributed to their metal center oxidation from Ru^ii^ to Ru^iii^. Compared to 1–4, the Ru^iii/ii^ redox potentials of [1-NH_3_]PF_6_ to [3-NH_3_]PF_6_ are anodically shifted by ∼0.12 V, mainly due to the π-donating capability of the Cl^−^ ligand in the former. Upon expanding the voltage window, unlike the second oxidation peak corresponding to Ru^iv/iii^ reported in our previous literature,^15^ these complexes exhibited no additional metal-centered oxidation waves except for the second oxidation peak assigned to cymene ligand oxidation at ∼1.6 V.^26^ However, a weak new wave marked by a rhombus appears at 0.75–0.88 V when scanning in the cathodic direction (Fig. 2c). This suggests that a disproportionation of Ru^iii^ species possibly occurs to give Ru^ii^ and Ru^iv^ species, and then Ru^iv^ is reduced to Ru^iii^ on the reverse scan. Taking [1-NH_3_]PF_6_ as an example, its Ru^iii^ intermediate formed by 1e^−^ oxidation is proposed to undergo rapid disproportionation to Ru^ii^ [1-NH_3_]^+^ and a Ru^iv^ imido intermediate and simultaneously reach equilibrium. Thus, the wave at 0.88 V on the reverse scan can be assigned to the reduction of Ru^iv^ species to Ru^iii^ species of [1-NH_3_]^+^. To further confirm the 1e^−^ oxidation to form a Ru^iii^ intermediate, CV was employed, with ferrocene added as the internal reference at an equimolar concentration to [1-NH_3_]^+^. As shown in Fig. S30, the diffusion coefficient of complex [1-NH_3_]^+^ (1.58 × 10^−5^ cm^2^ s^−1^) is very close to that of ferrocene (1.37 × 10^−5^ cm^2^ s^−1^), and the peak areas of [1-NH_3_]^+^ and ferrocene are almost the same in their respective CV plots, preliminarily indicating that the first oxidation wave of [1-NH_3_]^+^ involved only one electron transfer. The more critical evidence in the reversible charge transfer process is the slope analysis of *E vs.* lg[(*I*_l_ − *I*)/*I*] (where *E* is the potential and *I*_l_ is the limiting current).^27^ As shown in Fig. S31, a linear relationship was observed between *E* and lg[(*I*_p_ − *I*)/*I*] (where *I*_p_ is the peak current, used in place of *I*_l_ due to the irreversible oxidation wave of [1-NH_3_]^+^), with a slope *m* = 2.3*RT*/*nF* (where *R* is the ideal gas constant, *T* = 298.15 K, and *F* = 96 485C mol^−1^). The fitted slope from the experimental data was 0.84, yielding an electron transfer number *n* ≈ 0.84. These results collectively confirm that only a single electron transfer occurs at the electrode surface, followed by a redox disproportionation chemical step.

**Fig. 2:**
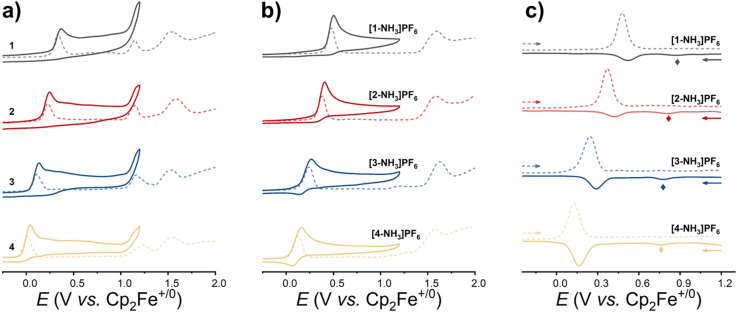
Electrochemical behavior of the title complexes in CH_3_CN solution. (a) CV (solid lines) and DPV (dotted lines) plots of 1–4; (b) CV (solid lines) and DPV (dotted lines) plots of [1-NH_3_]PF_6_ to [4-NH_3_]PF_6_; and (c) DPV plots of [1-NH_3_]PF_6_ to [4-NH_3_]PF_6_ in the anodic direction (dotted lines) and cathodic direction (solid lines). Conditions: [Ru] = 1 mM, scan rate 0.1 V s^−1^ for CV and 0.008 V s^−1^ for DPV, 0.1 M Bu_4_NPF_6_ as the supporting electrolyte, glassy carbon as the working electrode, platinum wire as the counter electrode, AgCl/Ag in saturated KCl aqueous solution as the reference electrode.

The electrochemical behavior of [1-NH_3_]PF_6_ to [4-NH_3_]PF_6_ in the presence of 0.05 M NH_3_ is shown in Fig. 3. Complexes [1-NH_3_]PF_6_ and [2-NH_3_]PF_6_ display a slightly increased catalytic current (*i*_cat_) over Ru^iii^ species with low onset potentials of 0.15 V and 0.10 V, respectively (inset figures of Fig. 3). Although Ru^iv^ species of the title complexes are not clearly observed in the CV studies, the catalytic current increases sharply at potentials over 0.6 V. For complexes [3-NH_3_]PF_6_ and [4-NH_3_]PF_6_, a catalytic current only appears at a high potential, and there is no catalytic current over Ru^iii^ species. Anodic currents (*i*_p_) of the Ru^iii/ii^ redox couple and the catalytic current of [1-NH_3_]PF_6_ to [4-NH_3_]PF_6_ in the presence of 0.05 M NH_3_ increase linearly with the square root of the scan rate (Fig. S33), indicating diffusion-controlled behavior of these ruthenium catalysts under test conditions. The rate constant (*k*_cat_) and maximum turnover frequency (TOF_max_) of [1-NH_3_]PF_6_ to [4-NH_3_]PF_6_ for ammonia oxidation are estimated. The diffusion coefficients (*D*_Ru_) and rate constants (*k*_cat_) of [1-NH_3_]PF_6_ to [4-NH_3_]PF_6_ are 1.58 × 10^−5^ cm^2^ s^−1^, 1.71 × 10^−5^ cm^2^ s^−1^, 1.82 × 10^−5^ cm^2^ s^−1^, and 1.94 × 10^−5^ cm^2^ s^−1^; and 4.5 s^−1^, 4.9 s^−1^, 5.2 s^−1^, and 5.4 s^−1^, respectively, which are determined from the dependence of *i*_p_ and *ν*^1/2^ based on the Randles–Sevcik relation (eqn (4)) and linear fitting of *i*_cat_/*i*_p_ with ν^−1/2^ based on eqn (5), respectively. The TOF_max_ values at a scan rate of 0.1 V s^−1^ based on eqn (6) are estimated to be 4.92 × 10^−2^ s^−1^, 5.48 × 10^−2^ s^−1^, 5.81 × 10^−2^ s^−1^, and 6.29 × 10^−2^ s^−1^.4
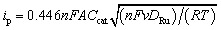
5
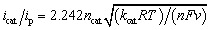
6
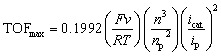


**Fig. 3:**
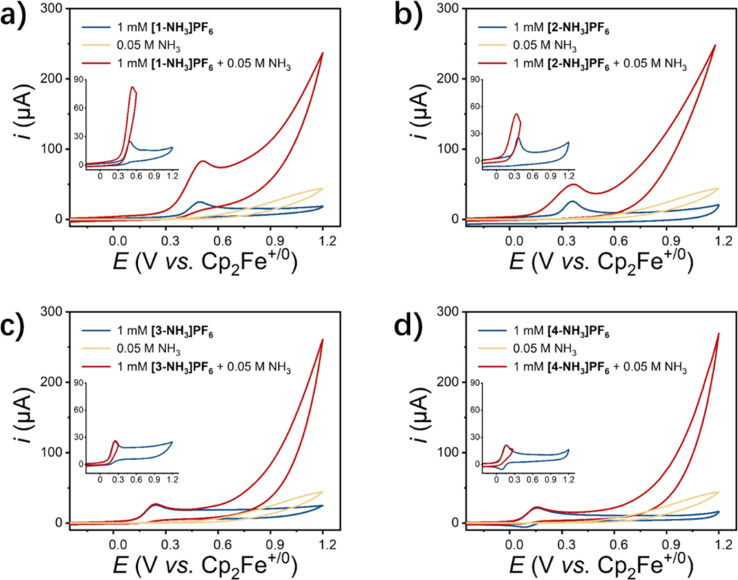
CV plots of (a) [1-NH_3_]PF_6_, (b) [2-NH_3_]PF_6_, (c) [3-NH_3_]PF_6_ and (d) [4-NH_3_]PF_6_ solution with/without 0.05 M NH_3_/NH_4_PF_6_ in CH_3_CN. Conditions: [Ru] = 1 mM, scan rate 0.1 V s^−1^, 0.1 M Bu_4_NPF_6_ as the supporting electrolyte, glassy carbon as the working electrode, platinum wire as the counter electrode, AgCl/Ag in saturated KCl aqueous solution as the reference electrode. Insets: CVs. in the voltage window of −0.25–1.2 V.

To confirm the electrocatalysis of [1-NH_3_]PF_6_ to [4-NH_3_]PF_6_ for NH_3_-to-N_2_H_4_ conversion, controlled potential coulometry (CPC) experiments are conducted in a conventional sealed three-electrode cell with 2.0 M NH_3_, 0.01 mM catalyst and 0.1 M [*n*-Bu_4_N]PF_6_ electrolyte in dried CH_3_CN under an Ar atmosphere. Carbon cloth (1 cm^2^), a Pt plate (1 cm^2^) and Ag/AgCl in saturated KCl aqueous solution are used as the working electrode, counter electrode and reference electrode, respectively. Possible gas products (such as N_2_, H_2_) are determined by the GC method (Fig. S1). Possible products in the electrolyte (such as N_2_H_4_, NO_2_^−^, NO_3_^−^) are quantitatively analyzed *via* chemical methods (Fig. S2–S5).^28–30^

To obtain general information for the CPC experiments, first, control experiments are employed. As shown in Table S7, only negligible N_2_H_4_ and N_2_ are generated at low applied potential (such as *E*_app_ 0.39 V) for 24 h. However, 21.6 µmol of N_2_H_4_ and 0.5 µmol of N_2_ are produced at *E*_app_ 0.8 V for 2 h, indicating that the bare electrode causes slow ammonia oxidation at relatively high potential. Subsequently, the complex [2-NH_3_]PF_6_ is chosen to investigate the relationship between catalytic performance over time and applied potential (Table 1 and Fig. S38, S39, Tables S8, S9). The amounts of N_2_H_4_ as an absolutely dominant anodic product and H_2_ as a cathodic product increase with an increase of the applied potential from 0.2 V to 1.0 V (Fig. S38). Other anode products of NO_2_^−^ and NO_3_^−^ are not determined due to the absence of oxygen sources in the catalytic system. Holding the applied potential at 0.8 V, the generation of N_2_H_4_ and H_2_ keeps increasing over time, however, the turnover frequency (TOF) and Faraday efficiency (FE) of N_2_H_4_ formation continuously decrease (Fig. S39 and Table S9). The loss of TOF and FE for long-term electrolysis might be caused by over-oxidation of the produced H_2_ in the sealed CPC cell. A decrease in *i*_cat_ under a H_2_ atmosphere indicating the current consumption of H_2_ oxidation cannot be ignored (Fig. S40). The *i*_cat_ remains almost unchanged after 100 consecutive CV cycles or electrolysis for 2 h, indicating that these Ru catalysts under catalytic conditions have satisfactory stability (Fig. S41 and S42). A thoroughly rinsed electrode after catalysis shows no ruthenium deposition and no catalytic activity, indicating solution-based electrocatalysis (Fig. S43–4S5 and Table S7). Notably, N_2_H_4_ is prone to undergo either catalytic oxidation or disproportionation decomposition to yield N_2_. In contrast, complexes [1-NH_3_]PF_6_–[4-NH_3_]PF_6_ exhibit good selectivity toward N_2_H_4_ formation. CV measurements of [1-NH_3_]PF_6_ in MeCN containing N_2_H_4_ confirm that they are inactive toward N_2_H_4_ oxidation (Fig. S54).

**Table 1:** The electrocatalytic performances of [1-NH_3_]PF_6_ to [4-NH_3_]PF_6_ in CH_3_CN^a^

Entry	Cat	[NH_3_] (M)	*E*_app_	Time (h)	TON_H_2__/*n*_H_2__(µmol)	TOF_H2_ (h^−1^)	TON_N_2_H_4__/*n*_N_2_H_4__(µmol)	TOF_N_2_H_4__ (h^−1^)	TON_N_2__/*n*_N_2__(µmol)	TOF_N_2__ (h^−1^)	FE_N_2_H_4__^b^ (%)	*S*_N_2_H_4__ c (%)
1	[1-NH_3_]PF_6_	2.0	0.39	24	17.2	0.7	16.5	0.7	Trace	—	86.3	100
					13.7		13.2					
2	[2-NH_3_]PF_6_	2.0	0.26	24	11.5	0.5	11.0	0.5	Trace	—	89.7	100
					9.2		8.8					
3	[3-NH_3_]PF_6_	2.0	0.13	24	Trace	—	Trace	—	Trace	—	—	—
4	[4-NH_3_]PF_6_	2.0	0.06	24	Trace	—	Trace	—	Trace	—	—	—
5	[1-NH_3_]PF_6_	2.0	0.8	2	377.3	188.7	356.2	178.1	5.3	2.7	88.2	98.6
					301.9		284.9		4.2			
6	[2-NH_3_]PF_6_	2.0	0.8	2	403.5	201.8	393.9	197.0	2.2	1.1	90.8	99.4
					322.8		315.2		1.8			
7	[3-NH_3_]PF_6_	2.0	0.8	2	430.1	215.1	418.2	209.1	4.3	2.2	93.5	99.0
					344.1		334.5		3.4			
8	[4-NH_3_]PF_6_	2.0	0.8	2	461.6	230.8	453.2	230.8	2.4	1.2	91.9	99.5
					369.3		362.5		1.9			
9	[4-NH_3_]PF_6_	0.05	0.8	2	156.6	78.3	151.0	75.5	1.5	0.8	87.6	99.0
					125.3		120.8		1.2			
10	5 (ref. 15a)	2.0	1.0	24	5870	244.6	5735	238.9	43.9	1.8	36.6	97.8
					2348		2293.8		17.56			
11	6 (ref. 15b)	2.0	1.0	2	801.5	400.8	721.5	360.8	13.7	6.9	99.2	98.1
					561.1		505.1		9.6			
12	7 (ref. 15c)	2.0	1.0	2	844.2	422.1	786.8	393.4	19.8	9.9	99.3	93.1
					616.2		566.4		14.3			

a Conditions: carbon cloth (1 cm^2^) as the working electrode, a platinum plate (1 cm^2^) as the counter electrode, AgCl/Ag in saturated KCl aqueous solution as the reference electrode, [cat] = 0.01 mM. The background is subtracted to obtain the number of moles of the products H_2_, N_2_ and N_2_H_4_.

b FE_N_2_H_4__ = 2 *n*_N_2_H_4__*F*/*it* × 100%.

c *S*_N_2_H_4__ = *n*_N_2_H_4__/(*n*_N_2__ + *n*_N_2_H_4__) × 100%.

To clarify the catalytic performance of the catalysts at the initial stage of Ru^iii^ species formation, we selected a potential 0.1 V lower than the Ru^iii/ii^ potential of the catalysts (0.39 V for [1-NH_3_]PF_6_, 0.26 V for [2-NH_3_]PF_6_, 0.13 V for [3-NH_3_]PF_6_, and 0.06 V for [4-NH_3_]PF_6_) as the applied potential in the CPC experiment. N_2_H_4_ and almost equiv. molar of H_2_ are produced in the [1-NH_3_]PF_6_ and [2-NH_3_]PF_6_ catalyst systems, and the generation of N_2_ could be negligible (entries 1 and 2). From comparison to the result of the control CPC experiments at *E*_app_ 0.39 V, this indicates that the catalytic activity originates from the catalyst but not the bare electrode. The catalytic conversion of NH_3_ to N_2_H_4_ achieves ∼100% selectivity. This behavior also aligns with our earlier ruthenium catalysts containing pyridylpyrrole operated at low applied potentials *via* bimolecular N–N coupling of Ru^iii^-amide to form N_2_H_4_.^15,18,19,20*a*^ As expected in the CV studies, no ammonia oxidation products (N_2_H_4_ or N_2_) are generated in the [3-NH_3_]PF_6_ and [4-NH_3_]PF_6_ catalyst systems, only over Ru^iii^ species at low potential (entries 3 and 4). Next, we carried out CPC experiments at higher applied potential *E*_app_ of 0.80 V. Correspondingly, the catalytic efficiency of [1-NH_3_]PF_6_ to [4-NH_3_]PF_6_ significantly increased. As shown in Table 1, entries 5–8, N_2_H_4_ is still determined as the dominant anodic product with a yield range from 284.9 µmol to 362.5 µmol. The turnover frequency (TOF) of N_2_H_4_ formation reaches 178.1 h^−1^ for [1-NH_3_]PF_6_, 197.0 h^−1^ for [2-NH_3_]PF_6_, 209.1 h^−1^ for [3-NH_3_]PF_6_ and 226.6 h^−1^ for [1-NH_3_]PF_6_. The selectivity and Faraday efficiency based on N_2_H_4_ formation are over 98.6% and 88.2%, respectively. In this work, [4-NH_3_]PF_6_ also shows excellent selectivity and high catalytic activity for the oxidation of low-concentration NH_3_ to generate N_2_H_4_. We carried out CPC experiments in a low-concentration NH_3_ solution (0.05 M) at the same potential. After 2 h, catalytic amounts of H_2_ (125.3 µmol), N_2_H_4_ (120.8 µmol) and N_2_ (1.2 µmol) were generated. This result breaks through the limitation of conventional catalytic systems that rely on high ammonia concentrations to achieve high selectivity (entry 9).

The CV and CPC experiments illustrate that complexes [1-NH_3_]PF_6_ and [2-NH_3_]PF_6_ for Ru^iii^ species and [1-NH_3_]PF_6_ to [4-NH_3_]PF_6_ for Ru^iv^ species readily undergo ammonia oxidation to generate N_2_H_4_. To explore the detailed mechanism of these complexes, we carried out theoretical calculations on the activation energy of each step in the catalytic procedure for the [1-NH_3_]PF_6_ and [4-NH_3_]PF_6_ catalysts. A summary of the proposed mechanism and alternative pathway is shown in Fig. 4.

**Fig. 4:**
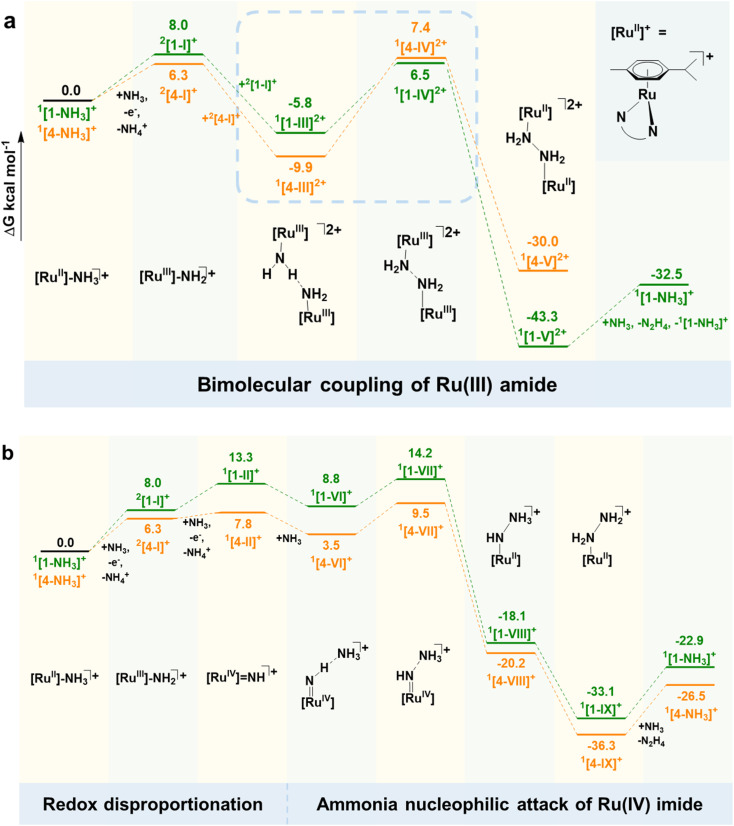
Calculated pathways of ammonia oxidation catalysed by [1-NH_3_]PF_6_ and [4-NH_3_]PF_6_, where the free energy changes (Δ*G*) of individual steps for [1-NH_3_]PF_6_ and [4-NH_3_]PF_6_ are presented in green and orange, respectively. (a) At low potential, hydrazine formation *via* bimolecular coupling of Ru^iii^ amide; (b) at high potential, hydrazine generation through ammonia nucleophilic attack of Ru^iv^ imide *via* redox disproportionation. [Ru^ii^]^+^ = [(*η*^6^-*p*-cymene)Ru(pp)]^+^. Δ*G* is calculated relative to Cp_2_Fe^+/0^, with values given in kcal mol^−1^.

As shown in Fig. 4a, complexes [1-NH_3_]^+^ and [4-NH_3_]^+^ are first oxidized to Ru^iii^-amide ^2^[1-I]^+^ and ^2^[4-I]^+^ (the left superscript shows the spin state) *via* a 1e^−^/H^+^ transfer process with Δ*G* of 8.0 and 6.3 kcal mol^−1^, respectively. Spin density distribution calculations (Fig. S55) reveal that the spin densities of ^2^[1-I]^+^ and ^2^[4-I]^+^ are primarily located on the Ru center (0.50 and 0.47 e^−^) and N atom of NH_2_ (0.50 and 0.50 e^−^), indicating that the Ru^iii^-amide species has Ru^ii^-aminyl character *via* inter-conversion of Ru^iii^–NH_2_^−^ ⇋ Ru^ii^–NH_2͘͘_˙. The Ru^ii^-aminyl species is an active intermediate and can readily generate N_2_H_4_*via* a bimolecular Ru^ii^–NH_2_^−^ coupling reaction.^15,18,19,20*a*^ For the [1-NH_3_]PF_6_ catalyst, theoretical calculations elucidate the coupling process between two ^2^[1-I]^+^ molecules, which proceeds through transition states ^1^[1-III]^2+^ and ^1^[1-IV]^2+^ to ultimately form the thermodynamically stable hydrazine-bridged bimetallic complex ^1^[1-V]^2+^. The catalytic cycle is completed by a mildly endothermic hydrazine dissociation process, with an overall energy barrier of 12.3 kcal mol^−1^.

This bimolecular coupling pathway is supported by the CPC experiment of [1-NH_3_]PF_6_ at low applied potential (0.39 V), where only N_2_H_4_ as an ammonia oxidation product is observed. In addition, its catalytic current over Ru^iii^ species is independent of the increase of [NH_3_] (10–50 mM), also indicating that ammonia seems not to be involved in N_2_H_4_ formation (Fig. S35) only over Ru^iii^ species. A similar bimolecular coupling pathway to form N_2_H_4_ over Ru^iii^ species is observed in our reported ruthenium catalysts,^15^ and has recently been confirmed in the [(trpy)(bpy^NMe2^)Ru(NH_3_)](PF_6_)_2_ catalyst system.^14^ Interestingly, in the bimolecular coupling pathway, the energy barrier for the conversion of ^1^[4-III]^2+^ to ^1^[4-V]^2+^ (17.3 kcal mol^−1^) is only 5 kcal mol^−1^ higher than that for ^1^[1-III]^2+^ to ^1^[1-V]^2+^. However, CV and CPC studies on the structurally analogous complex [4-NH_3_]PF_6_ demonstrate that its Ru^iii^ species (^2^[4-I]^+^) cannot trigger ammonia oxidation. This suggests that the difference in energy barrier does not play a decisive role in determining whether N–N bond formation occurs. From a kinetic perspective, an increased number of methyl groups (three methyl groups in [4-NH_3_]PF_6_) enhances steric hindrance, which disfavors the bimolecular coupling pathway and appears to be the dominant controlling factor.

Following the 1e^−^/H^+^ transfer process, the subsequent second 1e^−^/H^+^ oxidation to form Ru^iv^-imide species ^1^[1-II]^+^ and ^1^[4-II]^+^ is less endergonic with Δ*G* of 5.3 and 1.5 kcal mol^−1^, respectively. Compared to the first oxidation step, the significantly reduced Δ*G* values indicate thermodynamically more favorable formation of Ru^iv^-imide species. Analysis of the combined free energy changes reveals that the redox disproportionation of Ru^iii^-amide to generate Ru^II^-ammine and Ru^iv^-imide exhibits Δ*G* values of −2.7 and −4.8 kcal mol^−1^, respectively, demonstrating a highly spontaneous thermodynamic process. Therefore, the redox disproportionation pathway to form Ru^iv^-imide species is thermodynamically preferred over direct oxidation of Ru^iii^-amide, explaining the absence of an observable Ru^iv/iii^ redox couple under CV test conditions. Notably, the redox disproportionation of ^2^[4-I]^+^ seems to be more favorable than that of ^2^[1-I]^+^, which is less exergonic. Subsequently, the Ru^iv^-imide species ^1^[1-II]^+^ and ^1^[4-II]^+^ initiate ammonia oxidation *via* nucleophilic attack pathways. First, ^1^[1-II]^+^ and ^1^[4-II]^+^ readily interact with NH_3_ to generate ^1^[1-VI]^+^ and ^1^[4-VI]^+^ intermediates (Δ*G* = −4.5 and −4.3 kcal mol^−1^, respectively) due to the formation of a hydrogen bond between the H atom of the imide and the N atom of the approaching NH_3_. Subsequently, terminal N_2_H_4_-ligated ^1^[1-IX]^+^ and ^1^[4-IX]^+^ are formed through transition states ^1^[1-VII]^+^ and ^1^[4-VII]^+^ (Δ*G*^‡^ = 5.4 and 6.0 kcal mol^−1^, respectively) and intermediates ^1^[1-V^iii^]^+^ and ^1^[4-V^iii^]^+^. The formation of ^1^[1-IX]^+^ and ^1^[4-IX]^+^ N_2_H_4_-ligated intermediates through the reaction of ammonia and Ru^iv^-imides ^1^[1-II]^+^ and ^1^[4-II]^+^ is highly exergonic by 46.4 and 44.1 kcal mol^−1^, respectively. Finally, the catalytic cycle is restarted by endergonic evolution of N_2_H_4_ through N_2_H_4_-by-NH_3_ substitution of ^1^[1-IX]^+^ and ^1^[4-IX]^+^ (Δ*G* = 10.2 and 9.8 kcal mol^−1^, respectively). Unlike other ruthenium molecular catalysts only generating N_2_ as the N–N coupling product, the π-donor capability of the pyrrolyl group of the ancillary pp^−^ ligand in the title complexes helps to release N_2_H_4_ through N_2_H_4_-by-NH_3_ substitution of the N_2_H_4_-ligated intermediate, thus hindering N_2_H_4_ overoxidation to generate N_2_.^24^

The kinetic studies of the title complexes also support an ammonia nucleophilic attack route over Ru^iv^-imide species. As shown in Fig. S35 and S36, the catalytic current *i*_cat_ (at *E* = 1.2 V) linearly increases with the increase of [NH_3_] (0.010–0.050 M) and [cat] (0.2–1.0 mM), clearly indicating that there is a single-site molecular catalytic pathway. Notably, unlike *i*_cat_ at 0.05 M NH_3_ showing a linear relationship with *ν*^1/2^, the *i*_cat_ at 1.0 M NH_3_ almost does not change with increasing scan rate, indicating that the *i*_cat_ is no longer determined by the bulk diffusion of catalyst or NH_3_ but by the rate of regeneration of active Ru^iv^-imide species at the electrode.^11^ This seems to confirm that when ammonia is present in high concentrations, redox disproportionation to generate Ru^iv^-imide could be the slow step, and the nucleophilic coupling pathway could prevail, which is consistent with the recent results of mechanism studies of the [(trpy)(bpy^NMe2^)Ru(NH_3_)](PF_6_)_2_ catalyst system.^14^

In summary, a series of ruthenium(ii)-cymene NH_3_-ligated complexes are synthesized and fully characterized. By regulating the electronic structure of the ancillary ligand, the oxidation potential of the ruthenium center is gradually reduced from 0.49 V for [1-NH_3_]PF_6_ to 0.16 V for [4-NH_3_]PF_6_. Unlike the structurally analogous half-sandwich ferric catalyst [Cp*Fe(1,2-Ph_2_PC_6_H_4_NH)(NH_3_)]^+^ containing a phosphinoamido ligand to only generate N_2_H_4_ stoichiometrically,^17^[1-NH_3_]PF_6_ to [4-NH_3_]PF_6_ exhibit good performance for the selective electrocatalytic conversion of NH_3_ to N_2_H_4_ with at least 98.6% selectivity and 86.3% Faraday efficiency. The mechanism studies illustrate that the Ru^iii^-amide intermediate has radical Ru^ii^-aminyl character *via* the inter-conversion Ru^iii^–NH_2_ ⇋ Ru^ii^–NH_2͘͘_˙. Bimolecular coupling of the Ru^ii^-aminyl species readily generates a N_2_H_4_-bridged biruthenium intermediate in [1-NH_3_]PF_6_ and [2-NH_3_]PF_6_ catalyst systems, but not in [3-NH_3_]PF_6_ and [4-NH_3_]PF_6_ catalyst systems. Another pathway of N_2_H_4_ formation in [1-NH_3_]PF_6_ and [4-NH_3_]PF_6_, the nucleophilic attack of Ru^iv^-imide species by ammonia, is more feasible due to lower energy barriers of 5.4 kcal mol^−1^ and 6.0 kcal mol^−1^, compared to the bimolecular coupling pathway with energy barriers of 12.3 and 17.3 kcal mol^−1^, respectively. This single-site molecular catalytic pathway is supported by a linear relationship between the catalytic current and concentration of catalyst and ammonia.

## Author contributions

Xiao-Yi Yi as the corresponding author contributed to project design and paper revision. Xi Zhang mainly contributed to synthesis and electrocatalysis studies of the titled complexes, writing the preliminary draft. Shan Zhao and Guo Chen contributed to the assist with synthesis. Contribution of Liru Cao and Jian Lin lied in assisting with project design. Zhi-Yan Liu contributed to supplement data during the paper revision process. Piao He contributed to the DFT calculations.

## Conflicts of interest

The authors declare no competing financial interest.

## Supplementary Material

SC-OLF-D5SC08826G-s001

SC-OLF-D5SC08826G-s002

## Data Availability

The raw data including synthesis, characterization, and photophysical and catalytic properties of these complexes are available from the corresponding author, upon reasonable request. CCDC 1 (2320736), 2 (2324232), 3 (2321021), 4 (2321506) and [2-NH_3_]PF_6_ (2427138) contain the supplementary crystallographic data for this paper. The authors confirm that the data supporting the findings of this study are available within the article and/or its supplementary information (SI). Supplementary information: general methods for synthesis and characterization, crystallographic refinement, cyclic voltammetry experiments and electrolysis experiments, and DFT calculations. See DOI: https://doi.org/10.1039/d5sc08826g.
