# Supplementary material for: Selective NH3-to-N2H4 conversion electrocatalysed by ruthenium(ii)-cymene complexes
Source: Chem Sci. 2026 Jan 13;17(11):5595–603. doi: 10.1039/d5sc08826g (PMC12833753; doi:10.1039/d5sc08826g)
Supplement: SC-017-D5SC08826G-s001 [file SC-017-D5SC08826G-s001.pdf]

Supplementary Information (SI) for Chemical Science.
This journal is © The Royal Society of Chemistry 2026

Supplementary Information for

Selective electrocatalytic ammonia oxidation by ruthenium(II)-cymene complexes

Xi Zhang,[a] Shan Zhao,[a] Chen Zhou,[a] Guo Chen,[b] Liru Cao,[c] Jian Lin,[c] Chen Tang,[a] Zhi-Yan Liu,[a] Piao He,[a] and Xiao-Yi Yi*[a]

[a] College of Chemistry and Chemical Engineering, Central South University, Changsha, Hunan 410083, P. R. China

[b] School of Chemistry and Chemical Engineering, Southwest University, Chongqing, 400715, P. R. China

[c] CAS Key Laboratory of Science and Technology on Applied Catalysis, Dalian Institute of Chemical Physics, Chinese Academy of Sciences, Dalian, 116023, P. R. China

Corresponding authors: Xiao-Yi Yi
Fax: 86 731 88879616; Tel: 86 731 88879616;
E-mail address: xyyi@csu.edu.cn

Contents

1. General considerations 3

Materials 3

Synthesis and characterization 3

Additional experimental methods 5

2. Crystallographic data, ESI-MS, NMR, IR and UV-vis 9

Crystallographic data 9

^{1}H NMR, ^{13}C NMR, IR and ESI-MS 15

3. Electrochemical studies 27

CV 27

CPC experiment 34

4. Coordinates from Geometry Optimizations 45

Reference: 82

1. General considerations

Materials

All syntheses were carried using a standard high vacuum line, Schlenk containing an atmosphere of purified argon. Solvents for air- and moisture-sensitive manipulations were dried and deoxygenated using a JC-Meyer Phoenix solvent drying system. The ligands HL1 = 2-(1Hpyrrol-2-yl)pyridine, HL2 = 2-(4-methyl-1H-pyrrol-2-yl)pyridine, HL3 = 2-(3,5-dimethyl-1H-pyrrol-2-yl)pyridine, and HL4 = 2-(3,4,5-trimethyl-1H-pyrrol-2-yl)pyridine were prepared according to literature methods.[1, 2] All other chemicals were obtained from J&K Scientific Ltd and used without further purification.

Synthesis and characterization

All of chemicals were obtained from J&K Scientific in China. Ultradry solvents were treated by Solvent Drying System (J. C. Meyer, USA). The UV-visible spectra were measured by spectrophotometer Cary 8454 (Agilent Technologies, USA). Infrared spectra (KBr) were recorded on Nicolet 6700 spectrometer FT-IR spectrophotometer (Thermo Fisher Scientific, USA). Electrochemical measurements were accomplished with a CHENHUA CHI660E (China). Gas quantifications were conducted with a GC-2014C gas chromatograph (Shimadzu, Japan). Single crystal X-ray diffraction analysis was carried out at the Bruker Smart ApexII CCD diffractometer (Mo Kα radiation) (Germany). The NMR spectra were recorded on a Bruker AVANCE (III) 400 M spectrometer (Germany). The morphology of the samples was monitored through a scanning electron microscope (SEM). ESI-MS was performed in a Bruker Daltonik GmbH, Bremen mass spectrometer equipped with an electrospray ionization (ESI) source. Capillary temperature, 200 ^{o}C; Capillary voltage, 3500 V; Flow rate, 4 L/min; Hexapole, 400 Vpp.

Synthesis of complex 1. In N_2 atmosphere, mixture of HL1 ligand (144 mg, 1.0 mmol) and potassium tert-butoxide (112 mg, 1.0 mmol) in 5 mL ultra-dry CH_2Cl_2 were stirred for 1 h. The color of solution changed from colorless to pink. Ruthenium precursor [(η^6-*p*-cymene)$RuCl_2$]$_2$ (306 mg, 0.5 mmol) solution in 5 mL CH_2Cl_2 was slowly added at 0 ^{o}C. The reaction mixture was continued to stir for 4 h at 0 ^{o}C. The color of suspension solution changed to orange red. After filtration, filtrate was concentrated to ~3 mL. The orange-yellow solid as pure **1** was obtained by recrystallization through addition of Et_2O. The yield: 201 mg (51 %). ^{1}H NMR (400 MHz, $CDCl_3$): δ 8.75 (d, 1H), 7.48 (d, 1H), 7.34 (d, 2H), 6.77 (t, 1H), 6.70 (d, 1H), 6.36 – 6.30 (m, 1H), 5.57 (d, 1H), 5.44 (d, 1H), 5.33 (d, 1H), 5.28 (d, 1H), 2.66 (dt, 1H), 2.18 (s, 3H), 1.08 (d, 3H), 1.04 (d, 3H) ppm. ^{13}C NMR (101 MHz, $CDCl_3$): δ 155.61, 151.48, 137.03, 135.82, 133.75, 116.96, 116.51, 110.70, 108.37, 99.92, 98.66, 84.09, 82.37, 82.08, 80.20, 29.81, 21.28, 20.77, 17.68 ppm. IR (KBr, cm^{-1}): 3051 (m), 2964 (m), 2872 (m), 1925 (w), 1608 (s), 1531 (s), 1445 (s), 1381 (s), 1306 (m), 1153 (m), 1040 (s), 1013 (m), 941 (m), 868 (w), 745 (m), 717 (s), 613 (w), 515 (w). ESI-MS (in MeCN): *m/z* calcd. (found) for [**1**]$^+$: 379.1170 (379.0748).

Complex **2** was prepared like **1** using HL2 ligand. Yiled: 52%. ^{1}H NMR (400 MHz, $CDCl_3$): δ 8.72 (d, 1H), 7.49 – 7.40 (m, 1H), 7.24 (s, 1H), 7.10 (s, 1H), 6.72 (s, 1H), 6.49 (s, 1H), 5.54 (d, 1H), 5.44 (d, 1H), 5.31 (d, 1H), 5.27 (d, 1H), 2.69 (dt, 1H), 2.19 (s, 3H), 2.16 (s, 3H), 1.10 (d, 3H), 1.07 (d, 3H) ppm. ^{13}C NMR (101 MHz, $CDCl_3$): δ 155.49, 151.36, 136.69, 135.62, 133.24, 121.62, 116.34, 116.26, 109.14, 100.02, 97.97, 83.97, 82.33, 81.99, 80.41, 29.80, 21.36, 20.83, 17.64, 11.54 ppm. IR (KBr, cm^{-1}): 2956 (m), 2914 (w), 2860 (w), 1608 (s), 1539 (s), 1496 (s), 1439 (s), 1355 (s), 1318 (m), 1147 (m), 984 (w), 960 (m), 868 (w), 804 (w), 778 (s), 630 (w). ESI-MS (in MeCN): *m/z* calcd. (found) for [**2**]$^{+}$: 393.1313 (393.0905).

Complex **3** was prepared like **1** using HL3 ligand. Yiled: 52%. ^{1}H NMR (400 MHz, $CDCl_3$): δ 8.64 (d, 1H), 7.41 (ddd, 1H), 7.22 (d, 1H), 6.66 – 6.59 (m, 1H), 5.91 (s, 1H), 5.64 (d, 1H), 5.42 (d, 1H), 5.37 (d, 1H), 5.32 (d, 1H), 2.54 (s, 3H), 2.46 (dt, 1H), 2.31 (s, 3H), 2.26 (s, 3H), 0.99 (d, 3H), 0.98 (d, 3H) ppm. ^{13}C NMR (101 MHz, $CDCl_3$): δ 155.58, 151.69, 141.80, 135.39, 132.44, 122.86, 115.62, 115.05, 112.87, 101.36, 98.46, 84.63, 83.28, 82.17, 52.57, 29.84, 21.21, 20.89, 17.98, 16.44, 12.95 ppm. IR (KBr, cm^{-1}): 3066 (w), 2953 (m), 1603 (s), 1541 (s), 1489 (s), 1360 (s), 1277 (m), 1207 (w), 1157 (m), 1113 (w), 1030 (w), 972 (s), 850 (w), 768 (s), 696 (w), 636 (w), 519 (w). ESI-MS (in MeCN): *m/z* calcd. (found) for [**3**]$^{+}$: 407.1483 (407.1069).

Complex **4** was prepared like **1** using HL4 ligand. Yiled: 52%. ^{1}H NMR (400 MHz, $CDCl_3$): δ 8.61 (d, 1H), 7.36 (ddd, 1H), 7.22 (d, 1H), 6.56 (t, 1H), 5.59 (d, 1H), 5.41 (d, 1H), 5.37 (d, 1H), 5.32 (d, 1H), 2.53 – 2.43 (m, 4H), 2.25 (s, 3H), 2.23 (s, 3H), 1.99 (s, 3H), 1.00 (d, 3H), 0.98 (d, 3H) ppm. ^{13}C NMR (101 MHz, $CDCl_3$): δ 155.47, 151.65, 140.16, 135.18, 131.41, 120.91, 117.79, 115.36, 114.38, 100.69, 98.79, 84.28, 83.08, 82.48, 78.92, 29.82, 21.11, 21.07, 17.92, 14.80, 10.68, 9.18 ppm. IR (KBr, cm^{-1}): 3045 (w), 2966 (w), 2903 (m), 2853 (m), 1600 (s), 1537 (s), 1477 (s), 1360 (s), 1267 (m), 1211 (m), 1151 (m), 1011 (m), 932 (m), 851 (w), 777 (s), 669 (w), 644 (w), 525 (w), 438 (w). ESI-MS (in MeCN): *m/z* calcd. (found) for [**4**]$^{+}$: 421.1646 (421.1218).

Synthesis of complex [1-NH_3]PF_6. A mixture of **1** and one equiv. of $AgPF_6$ in 5 mL CH_3CN was stirred for 1 h, and the suspension solution was filtered. The NH_3 gas was bubbled into the filtrate for 1 h. The greyish-green solid as pure [**1-NH_3**]PF_6 was obtained by recrystallization through addition of Et_2O. The yield: 72 mg (61 %). ^{1}H NMR (400 MHz, $CDCl_3$): δ 8.83 (d, 1H), 7.66 – 7.61 (m, 1H), 7.38 (d, 1H), 7.21 (s, 1H), 6.98 (dd, 1H), 6.73 (dd, 1H), 6.33 (dd, 1H), 5.76 (d, 1H), 5.68 (d, 1H), 5.61 (d, 1H), 5.56 (d, 1H), 2.53 (dt, 1H), 2.22 (s, 3H), 2.06 (s, 3H), 1.07 (d, 3H), 1.01 (d, 3H) ppm.

The similar method was used for synthesis of [**2-NH_3**]PF_6 - [**4-NH_3**]PF_6.

[**2-NH_3**]PF_6, yield: 65%. ^{1}H NMR (400 MHz, $CDCl_3$): δ 8.78 (d, 1H), 7.63 – 7.54 (m, 1H), 7.28 (d, 1H), 6.98 (s, 1H), 6.95 – 6.88 (m, 1H), 6.51 (s, 1H), 5.74 (d, 1H), 5.68 (d, 1H), 5.58 (d, 1H), 5.54 (d, 1H), 2.56 (dt, 1H), 2.19 (s, 3H), 2.17 (s, 3H), 2.06 (s, 3H), 1.10 (d, 3H), 1.03 (d, 3H) ppm.

[**3-NH_3**]PF_6, yield: 68%. ^{1}H NMR (400 MHz, $CDCl_3$): δ 8.71 (d, 1H), 7.59 – 7.48 (m, 1H), 7.25 (s, 1H), 6.88 – 6.72 (m, 1H), 5.89 (s, 1H), 5.78 (d, 1H), 5.75 (d, 1H), 5.62 (d, 1H), 5.52

(d, 1H), 2.44 (s, 3H), 2.36 (dd, 1H), 2.32 (s, 3H), 2.28 (s, 3H), 2.15 (d, 3H), 0.94 (d, 3H), 0.89 (d, 3H) ppm.

[**4-NH**$_3$]PF_6, yield: 63%. ^{1}H NMR (400 MHz, $CDCl_3$): δ 8.68 (d, 1H), 7.50 (dd, 1H), 7.28 (s, 1H), 6.76 (t, 1H), 5.77 (d, 1H), 5.73 (d, 1H), 5.62 (d, 1H), 5.53 (d, 1H), 2.40 (d, 3H), 2.36 (d, 1H), 2.31 (s, 3H), 2.21 (s, 3H), 2.11 (s, 3H), 1.98 (s, 3H), 0.96 (d, 3H), 0.90 (d, 3H) ppm.

Additional experimental methods

Gas Chromatography (GC) methods

Gas quantification was performed using a molecular sieve column (Porapak-N 80/100 mesh 3.2mm*2.1mm*1.0M, Molecular Sieve 13X 80/100 mesh 3.2mm*2.1mm*3.0M) attached to a thermal conductivity detector. Ar was used as the carrier gas. Standard curves were generated by direct injection of standard mixture gas of H_2 and N_2. The mixture gas (100 μL, 200 μL, 300 μL, 400 μL, 500 μL, 600 μL, 700 μL, 800 μL, 900 μL, 1000 μL) at headspace of reactor was manually injected to the GC using a gastight syringe (SGE Analytical Science).

Notably, the oxygen signal (aerobic contamination) in the cell of CPC experiments is inevitably observed. The aerobic contamination was estimated by assuming air as 4/1 mixture of N_2/O_2. Thus, the generated N_2 in the CPC experiments was quantified after correction of aerobic contamination estimated from the O_2 signal.

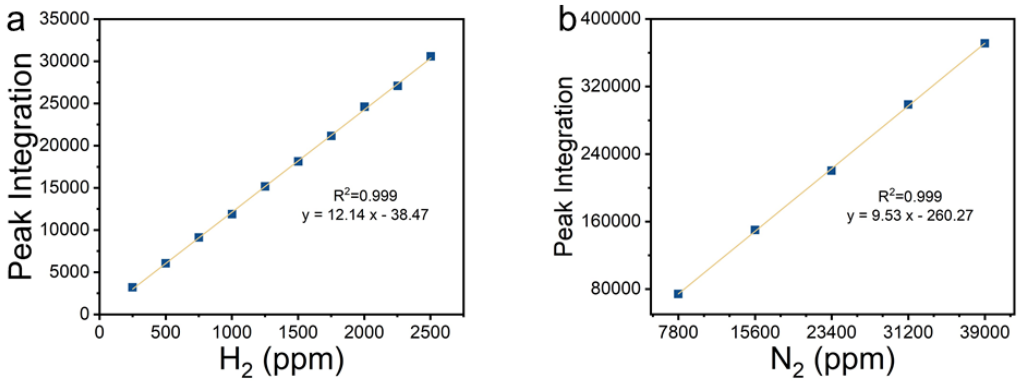

Supplementary Fig. 1 | Gas Chromatography calibration lines for H_2 and N_2.

N_2H_4 test

The N_2H_4 content in the electrolyte was determined by the method of Watt and Chrisp.[3] First, 4 g *p*-$C_9H_{11}NO$ was dissolved in 200 mL 95% ethanol and 20 mL 37% HCl to give a colour reagent. The concentration–absorbance curves were calibrated by measuring a series of standard solutions with different N_2H_4 concentrations

Calibration. For the N_2H_4 standard solutions, 0, 0.2, 0.4, 0.8, 1.6, 2.4, 3.2, 4.0 mL of 1 mg L^{-1} N_2H_4 solutions with the corresponding concentration were transferred to different test tubes. Then 4 mL of colour reagent was added and diluted to 10 mL. The solution was well shaken and allowed to stand for 20 min at room temperature. The contents were analysed with UV–vis spectroscopy using λ = 455.0 nm.

Procedure of quantification of N_2H_4 in CPC experiments: A given volume (0.10 mL or 0.010 mL) of electrolyte taken by high-accuracy syringe was mixed color reagent (2 mL) and then diluted to 10 mL. The resulting solution was shaken and allowed to stand for 20

min at room temperature, and then was analyzed with UV-vis spectroscopy using λ = 455.0 nm. Each sample is measured twice. To ensure the work point is in the calibration range, 0.10 mL or 0.010 mL and even fewer volume of electrolyte is taken.

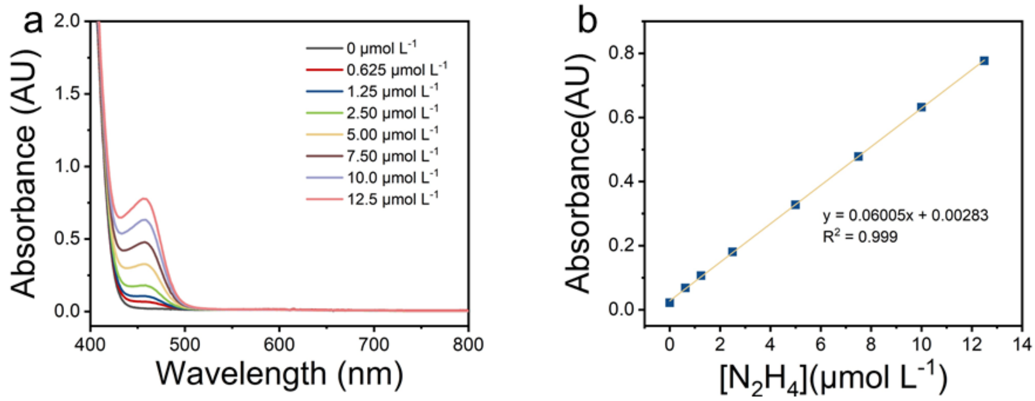

Supplementary Fig. 2 | (a) UV-Vis absorption spectra of standard N_2H_4 solutions (0, 0.2, 0.4, 0.8, 1.6, 2.4, 3.2 and 4.0 mL). (b) Calibration curve used for calculation of N_2H_4 concentrations.

NO_2^- test

Colour reagent **A** (1 mM) was prepared by dissolving sulfanilamide (17.2 mg, 0.1 mmol) in 5 mL of 37% HCl, then diluting the solution to 100 mL with DI water. Colour reagent **B** (1 mM) was prepared by dissolving 1-naphthylamine (14.3 mg, 0.1 mmol) in 5 mL of 37% HCl and then diluting the solution to 100 mL with DI water. Reagents **A** and **B** were stored in the dark at room temperature. Nitrite standard solutions were prepared by dissolving 69.0 mg (1 mmol) of $NaNO_2$ in 100.0 mL of DI water. The resulting 10 mM $NaNO_2$ solution was further diluted with water to give solutions of 20.0 μM.[4]

Calibration. For the nitrite standard curve, 0, 2.0, 4.0, 6.0 and 8.0 mL of as-prepared nitrite standard solutions ($NaNO_2$ aqueous solution) were transferred to different test tubes. Reagent **A** (1 mL) and reagent **B** (1 mL) were added to the test tubes and diluted to 10 mL. The mixtures were well shaken and allowed to stand for 5 min at room temperature before being analysed by UV–vis spectroscopy with a characteristic absorption band at 518.0 nm.

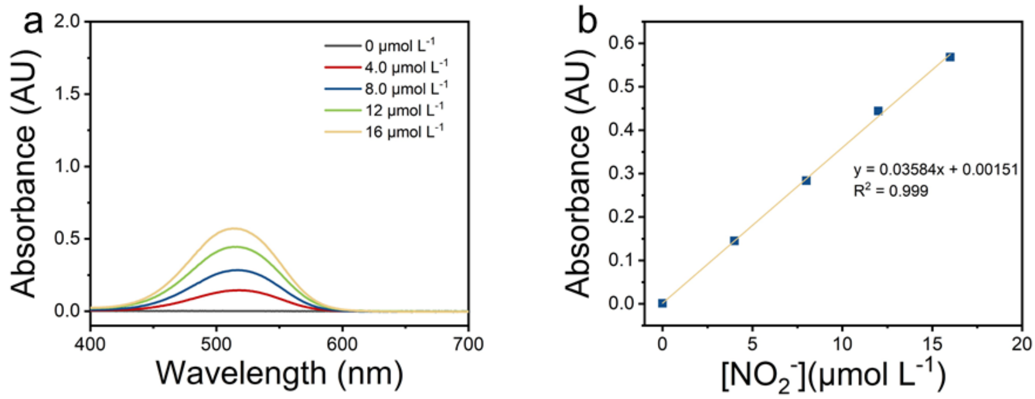

Supplementary Fig. 3 | (a) UV-Vis absorption spectra of standard $NaNO_2$ aqueous solution (0, 2.0, 4.0, 6.0 and 8.0 mL). (b) Calibration curve used for calculation of NO_2^- concentrations.

NO_3^- test

An ammonium sulfamate solution (2 mM) was prepared by dissolving 22.8 mg (0.2 mmol) of ammonium sulfamate in 5 mL of 37% HCl. This was then diluted to 100 mL with DI water. A nitrate standard solution was prepared by dissolving 8.5 mg (0.1 mmol) of Na NO_3 in 100.0 mL of DI water.[5]

Calibration. For the nitrite standard curve, 0, 0.2, 0.4, 0.6, 0.8 and 1 mL of as-prepared nitrate standard solutions ($NaNO_3$ aqueous solutions) were transferred to different test tubes. Ammonium sulfamate solution (1 mL) was added to the test tubes and the solutions were diluted to 10 mL. The mixtures were well shaken and allowed to stand for 5 min at room temperature before being analysed by UV–vis spectroscopy with a characteristic absorption band at 219.0 nm.

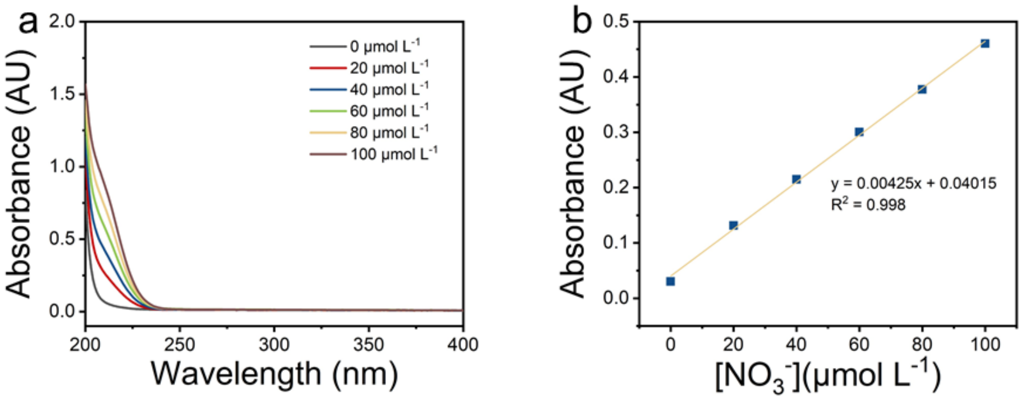

Supplementary Fig. 4 | (a) UV-Vis absorption spectra standard $NaNO_3$ solution (0, 0.2, 0.4, 0.6, 0.8 and 1 mL). (b) Calibration curve used for calculation of NO_3^- concentrations.

NH_3 test

The indophenol blue method was used to quantify the concentration of NH_3. Solution **A** (containing 1 M NaOH, 5 wt% salicylic acid and 5 wt% sodium citrate), solution **B** (containing 0.05 M sodium hypochlorite solution) and solution **C** (1 wt% sodium nitroferricyanide (III) dehydrate) were prepared.[6]

Calibration. For the NH_3 standard solutions, 0, 2.0, 5.0, 10.0, 20.0, 30.0 and 40.0 μL of 1 mg mL^{-1} NH_4Cl solutions with the corresponding concentration were transferred to 10 mL test tubes. Then, 2 mL solution **A**, 1 mL solution **B** and 0.2 mL solution **C** were added and the mixture was diluted to 10 mL. The resulting solution was allowed to stand for 2 h. The contents were analysed with UV–vis spectroscopy using λ = 660.0 nm.

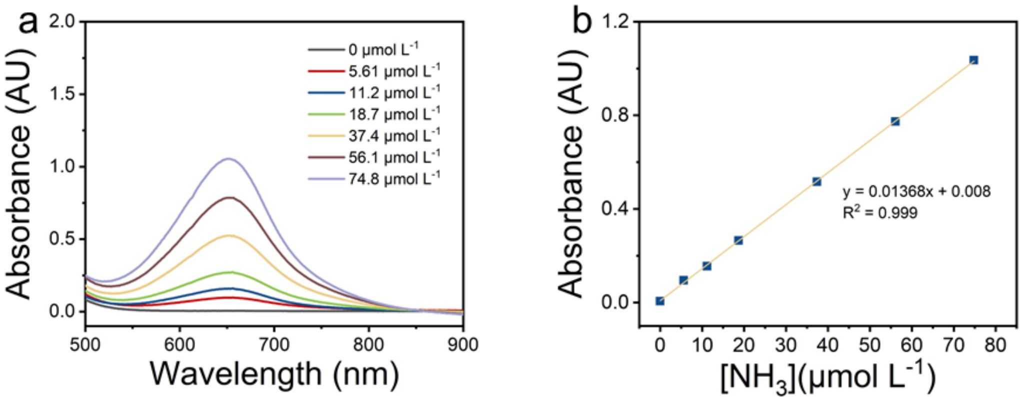

Supplementary Fig. 5 | (a) UV-Vis absorption spectra of standard NH_3 solution (0, 3.0, 6.0, 10.0, 20.0, 30.0 and 40.0 μL). (b) Calibration curve used for calculation of NH_3 concentrations.

DFT calculation

The free energies of the reaction species were calculated using density functional theory with the Gaussian 16 software package[7]. The geometries of the catalyst models were fully optimized with the PBE0 functional[8] including the DFT-D3 dispersion correction with BJ-damping[9] and the def2-SVP basis set. All pertinent spin states including low and high spin were evaluated for the complexes in their reactant, transition and product states. The lowest free energy spin multiplicities were selected to calculate thermodynamics (ΔG) and free energy barriers ($\Delta G^{\ddagger}$) for the studied reactions. Single-point calculations for all stationary points were performed with the same functional and a larger basis set, def2-TZVP basis set[10, 11], to obtain more accurate free energies. The SMD implicit solvation model[12] was used to account for the solvation effect of MeCN (ε = 35.688). The redox potentials were calculated for all individual steps from ΔG of the electron transfer reactions using the standard relationship $E = -\Delta G/(nF)$, where ΔG corresponds to electron transfer free energies,. n is the number of electrons being transferred and F is the Faraday constant.

2. Crystallographic data, ESI-MS, NMR, IR and UV-vis

Crystallographic data

Supplementary Table 1 | Crystallographic data of **1** - **4** and [**2-NH$_3$**]PF_6.

Compound<br>CCDC	**1**<br>2320736	**2**<br>2324232	**3**<br>2321021	**4**<br>2321506	**[2-NH$_3$]PF$_6$**<br>2427138
Empirical formula	$C_{19}H_{21}ClN_2Ru$	$C_{20}H_{23}ClN_2Ru$	$C_{21}H_{27}ClN_2ORu$	$C_{22}H_{27}ClN_2Ru$	$C_{80}H_{104}F_{24}N_{12}P_4Ru_4$
Formula weight	413.90	427.92	459.96	455.97	2217.91
Crystal system	monoclinic	monoclinic	orthorhombic	monoclinic	orthorhombic
Space group	$P2_1/c$	$P2_1/c$	Pbca	$P2_1/c$	$P2_12_12_1$
a/Å	7.9304(8)	16.5485(6)	13.866(4)	17.0430(8)	14.26280(10)
b/Å	12.1713(13)	7.7864(3)	9.926(2)	7.7147(4)	14.3393(2)
c/Å	18.2053(19)	14.8165(6)	29.844(8)	16.6516(8)	42.7074(4)
α/°	90	90	90	90	90
β/°	98.604(6)	104.586(2)	90	113.872(3)	90
γ/°	90	90	90	90	90
V/[Å^3]	1737.5(3)	1847.62(12)	4107.4(18)	2002.08(18)	8734.46(16)
Z	4	4	8	4	4
ρ_{calcd} [g cm^{-3}]	1.582	1.538	1.488	1.513	1.687
μ [mm^{-1}]	1.056	0.996	0.905	0.924	7.090
F(000)	840.0	872.0	1888.0	936.0	4480.0
R_{int}	0.0214	0.0388	0.0495	0.0367	0.0705
[a]GooF	1.033	1.021	1.009	1.023	1.089
[b]R_1, [c]wR_2 [$I>2\sigma(I)$]	0.0237/0.0575	0.0330/0.0615	0.0395/0.0837	0.0337/0.0733	0.0699/0.1823
R_1, wR_2 [all data]	0.0301/0.0607	0.0552/0.0682	0.0750/0.0971	0.0491/0.0797	0.0771/0.1873

[a]GooF = $[\Sigma w(|F_o| - |F_c|)^2/(N_{obs} - N_{param})]^{½}$.

[b]$R_1=\Sigma||F_o|-|F_c||/\Sigma|F_o|$. [c]$wR_2$ $[(\Sigma w|F_o| -|F_c|)^2/\Sigma w^2|F_o|^2]½$.

Supplementary Table 2 | Solid-state structure, bond lengths (Å) and bond angles (°) of **1**.

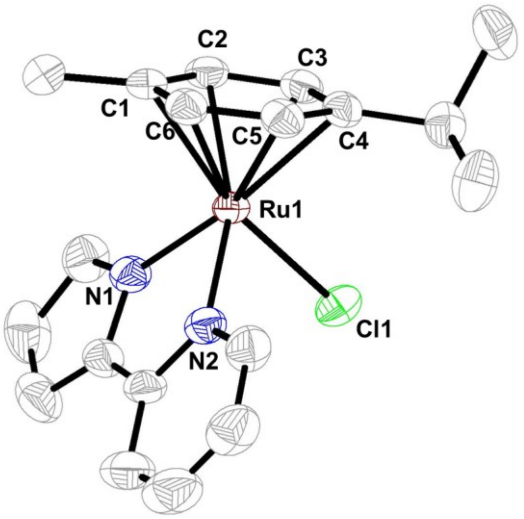

Bond lengths (Å) and angles (°) of **1**:

**Bond Distances(Å)**			
Ru(1)-N(1)	2.0547(17)	Ru(1)-C(4)	2.219(2)
Ru(1)-N(2)	2.1060(17)	Ru(1)-C(5)	2.199(2)
Ru(1)-C(1)	2.188(2)	Ru(1)-C(6)	2.177(2)
Ru(1)-C(2)	2.177(2)	Ru(1)-Cl(1)	2.4166(6)
Ru(1)-C(3)	2.171(2)		
**Bond Angles (º)**			
N(1)-Ru(1)-N(2)	76.86(7)	Cl(1)-Ru(1)-C(4)	91.24(6)
N(1)-Ru(1)-C(1)	92.55(8)	Cl(1)-Ru(1)-C(5)	118.88(6)
N(1)-Ru(1)-C(2)	95.95(8)	Cl(1)-Ru(1)-C(6)	156.64(6)
N(1)-Ru(1)-C(3)	122.90(8)	C(1)-Ru(1)-C(2)	38.09(8)
N(1)-Ru(1)-C(4)	160.86(8)	C(1)-Ru(1)-C(3)	68.57(8)
N(1)-Ru(1)-C(5)	154.34(8)	C(1)-Ru(1)-C(4)	81.74(8)
N(1)-Ru(1)-C(6)	117.17(8)	C(1)-Ru(1)-C(5)	68.27(8)
N(1)-Ru(1)-Cl(1)	85.92(5)	C(1)-Ru(1)-C(6)	37.66(9)
N(2)-Ru(1)-C(1)	120.91(8)	C(2)-Ru(1)-C(3)	37.44(8)
N(2)-Ru(1)-C(2)	158.30(8)	C(2)-Ru(1)-C(4)	68.36(8)
N(2)-Ru(1)-C(3)	159.19(8)	C(2)-Ru(1)-C(5)	79.59(8)
N(2)-Ru(1)-C(4)	121.72(7)	C(2)-Ru(1)-C(6)	67.60(9)
N(2)-Ru(1)-C(5)	97.96(8)	C(3)-Ru(1)-C(4)	38.10(8)
N(2)-Ru(1)-C(6)	97.17(8)	C(3)-Ru(1)-C(5)	67.18(8)
N(2)-Ru(1)-Cl(1)	84.14(5)	C(3)-Ru(1)-C(6)	80.05(8)
Cl(1)-Ru(1)-C(1)	153.90(6)	C(4)-Ru(1)-C(5)	37.06(8)
Cl(1)-Ru(1)-C(2)	116.11(6)	C(4)-Ru(1)-C(6)	68.14(8)
Cl(1)-Ru(1)-C(3)	90.46(6)	C(5)-Ru(1)-C(6)	37.77(8)

Supplementary Table 3 | Solid-state structure, bond lengths (Å) and bond angles (°) of **2**.

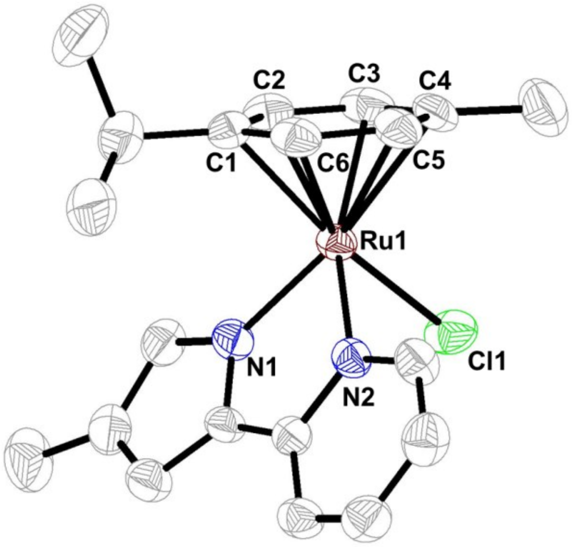

Bond lengths (Å) and angles (°) of **2**:

**Bond Distances(Å)**			
Ru(1)-N(1)	2.058(2)	Ru(1)-C(4)	2.235(3)
Ru(1)-N(2)	2.106(2)	Ru(1)-C(5)	2.191(3)
Ru(1)-C(1)	2.184(3)	Ru(1)-C(6)	2.162(3)
Ru(1)-C(2)	2.173(3)	Ru(1)-Cl(1)	2.4133(8)
Ru(1)-C(3)	2.189(3)		
**Bond Angles (º)**			
N(1)-Ru(1)-N(2)	76.87(9)	Cl(1)-Ru(1)-C(4)	90.88(8)
N(1)-Ru(1)-C(1)	93.08(10)	Cl(1)-Ru(1)-C(5)	116.48(9)
N(1)-Ru(1)-C(2)	96.41(10)	Cl(1)-Ru(1)-C(6)	154.46(8)
N(1)-Ru(1)-C(3)	122.86(11)	C(1)-Ru(1)-C(2)	38.16(10)
N(1)-Ru(1)-C(4)	160.01(11)	C(1)-Ru(1)-C(3)	68.41(11)
N(1)-Ru(1)-C(5)	155.55(11)	C(1)-Ru(1)-C(4)	81.28(11)
N(1)-Ru(1)-C(6)	117.91(10)	C(1)-Ru(1)-C(5)	68.53(11)
N(1)-Ru(1)-Cl(1)	86.87(7)	C(1)-Ru(1)-C(6)	37.74(11)
N(2)-Ru(1)-C(1)	117.64(10)	C(2)-Ru(1)-C(3)	37.11(11)
N(2)-Ru(1)-C(2)	155.28(10)	C(2)-Ru(1)-C(4)	67.38(12)
N(2)-Ru(1)-C(3)	159.96(11)	C(2)-Ru(1)-C(5)	79.24(12)
N(2)-Ru(1)-C(4)	122.74(11)	C(2)-Ru(1)-C(6)	67.57(11)
N(2)-Ru(1)-C(5)	96.97(10)	C(3)-Ru(1)-C(4)	37.33(12)
N(2)-Ru(1)-C(6)	94.25(10)	C(3)-Ru(1)-C(5)	66.57(12)
N(2)-Ru(1)-Cl(1)	85.03(6)	C(3)-Ru(1)-C(6)	79.84(11)
Cl(1)-Ru(1)-C(1)	156.74(8)	C(4)-Ru(1)-C(5)	36.78(11)
Cl(1)-Ru(1)-C(2)	118.70(8)	C(4)-Ru(1)-C(6)	68.04(11)
Cl(1)-Ru(1)-C(3)	92.14(8)	C(5)-Ru(1)-C(6)	38.17(11)

Supplementary Table 4 | Solid-state structure, bond lengths (Å) and bond angles (°) of **3**.

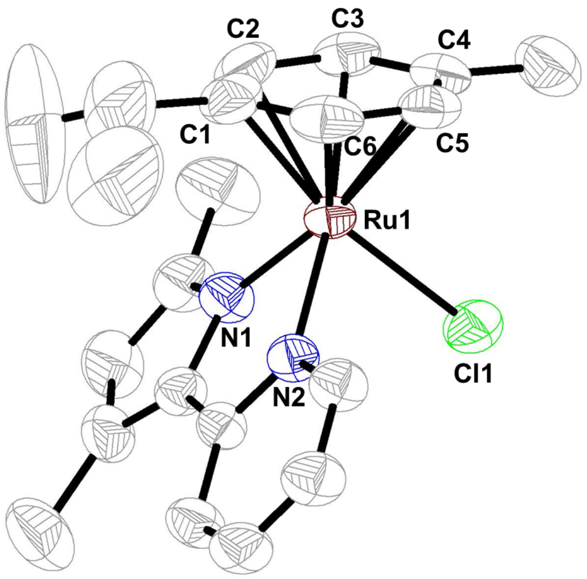

Bond lengths (Å) and angles (°) of **3**:

**Bond Distances(Å)**			
Ru(1)-N(1)	2.074(3)	Ru(1)-C(4)	2.247(4)
Ru(1)-N(2)	2.113(3)	Ru(1)-C(5)	2.223(4)
Ru(1)-C(1)	2.200(4)	Ru(1)-C(6)	2.181(4)
Ru(1)-C(2)	2.158(4)	Ru(1)-Cl(1)	2.4339(11)
Ru(1)-C(3)	2.213(4)		
**Bond Angles (º)**			
N(1)-Ru(1)-N(2)	77.20(11)	Cl(1)-Ru(1)-C(4)	88.60(11)
N(1)-Ru(1)-C(1)	105.85(16)	Cl(1)-Ru(1)-C(5)	99.08(11)
N(1)-Ru(1)-C(2)	91.68(15)	Cl(1)-Ru(1)-C(6)	130.54(13)
N(1)-Ru(1)-C(3)	105.04(14)	C(1)-Ru(1)-C(2)	37.97(16)
N(1)-Ru(1)-C(4)	136.90(13)	C(1)-Ru(1)-C(3)	68.28(16)
N(1)-Ru(1)-C(5)	170.82(12)	C(1)-Ru(1)-C(4)	80.73(16)
N(1)-Ru(1)-C(6)	140.31(15)	C(1)-Ru(1)-C(5)	68.17(17)
N(1)-Ru(1)-Cl(1)	86.73(9)	C(1)-Ru(1)-C(6)	38.15(16)
N(2)-Ru(1)-C(1)	100.74(15)	C(2)-Ru(1)-C(3)	37.45(15)
N(2)-Ru(1)-C(2)	132.60(15)	C(2)-Ru(1)-C(4)	67.21(16)
N(2)-Ru(1)-C(3)	169.01(14)	C(2)-Ru(1)-C(5)	79.37(16)
N(2)-Ru(1)-C(4)	144.66(13)	C(2)-Ru(1)-C(6)	67.74(17)
N(2)-Ru(1)-C(5)	110.34(13)	C(3)-Ru(1)-C(4)	36.81(13)
N(2)-Ru(1)-C(6)	91.98(14)	C(3)-Ru(1)-C(5)	66.55(15)
N(2)-Ru(1)-Cl(1)	83.92(9)	C(3)-Ru(1)-C(6)	79.44(16)
Cl(1)-Ru(1)-C(1)	167.24(13)	C(4)-Ru(1)-C(5)	37.09(14)
Cl(1)-Ru(1)-C(2)	142.05(13)	C(4)-Ru(1)-C(6)	67.25(16)
Cl(1)-Ru(1)-C(3)	106.86(12)	C(5)-Ru(1)-C(6)	37.17(14)

Supplementary Table 5 | Solid-state structure, bond lengths (Å) and bond angles (°) of **4**.

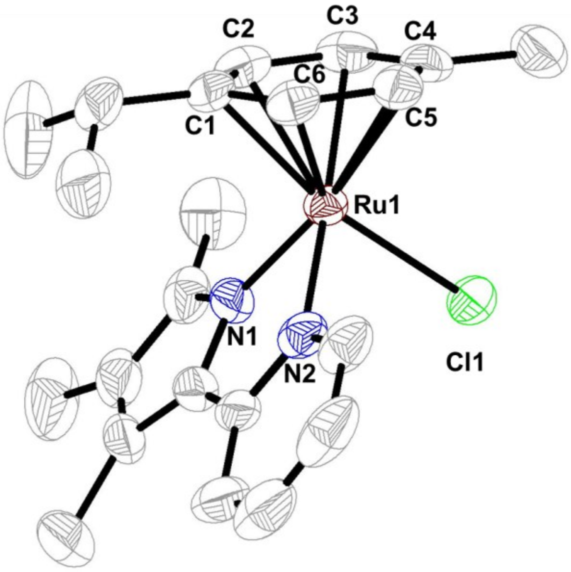

Bond lengths (Å) and angles (°) of **4**:

**Bond Distances(Å)**			
Ru(1)-N(1)	2.073(2)	Ru(1)-C(4)	2.242(3)
Ru(1)-N(2)	2.101(2)	Ru(1)-C(5)	2.215(3)
Ru(1)-C(1)	2.212(3)	Ru(1)-C(6)	2.162(3)
Ru(1)-C(2)	2.163(3)	Ru(1)-Cl(1)	2.4239(7)
Ru(1)-C(3)	2.202(3)		
**Bond Angles (º)**			
N(1)-Ru(1)-N(2)	76.63(10)	Cl(1)-Ru(1)-C(4)	88.01(8)
N(1)-Ru(1)-C(1)	100.66(10)	Cl(1)-Ru(1)-C(5)	104.33(8)
N(1)-Ru(1)-C(2)	92.63(10)	Cl(1)-Ru(1)-C(6)	138.65(8)
N(1)-Ru(1)-C(3)	111.32(11)	C(1)-Ru(1)-C(2)	37.85(10)
N(1)-Ru(1)-C(4)	145.65(11)	C(1)-Ru(1)-C(3)	68.49(11)
N(1)-Ru(1)-C(5)	168.85(10)	C(1)-Ru(1)-C(4)	81.15(11)
N(1)-Ru(1)-C(6)	132.30(11)	C(1)-Ru(1)-C(5)	68.21(11)
N(1)-Ru(1)-Cl(1)	86.76(7)	C(1)-Ru(1)-C(6)	37.75(11)
N(2)-Ru(1)-C(1)	103.77(10)	C(2)-Ru(1)-C(3)	37.67(12)
N(2)-Ru(1)-C(2)	138.15(11)	C(2)-Ru(1)-C(4)	67.66(12)
N(2)-Ru(1)-C(3)	169.43(10)	C(2)-Ru(1)-C(5)	78.89(11)
N(2)-Ru(1)-C(4)	136.76(11)	C(2)-Ru(1)-C(6)	67.00(11)
N(2)-Ru(1)-C(5)	104.83(11)	C(3)-Ru(1)-C(4)	37.14(11)
N(2)-Ru(1)-C(6)	90.49(10)	C(3)-Ru(1)-C(5)	65.98(11)
N(2)-Ru(1)-Cl(1)	85.97(6)	C(3)-Ru(1)-C(6)	79.01(11)
Cl(1)-Ru(1)-C(1)	168.83(8)	C(4)-Ru(1)-C(5)	36.41(11)
Cl(1)-Ru(1)-C(2)	134.37(9)	C(4)-Ru(1)-C(6)	67.08(11)
Cl(1)-Ru(1)-C(3)	101.12(9)	C(5)-Ru(1)-C(6)	37.43(11)

Supplementary Table 6 | Solid-state structure, bond lengths (Å) and bond angles (°) of **[2-NH$_3$]PF$_6$**.

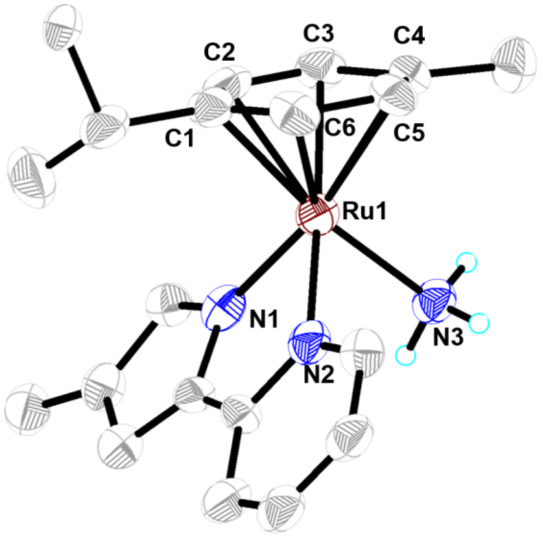

Bond lengths (Å) and angles (°) of **[2-NH$_3$]PF$_6$**:

**Bond Distances(Å)**			
Ru(1)-N(1)	2.042(10)	Ru(1)-C(3)	2.138(12)
Ru(1)-N(2)	2.111(11)	Ru(1)-C(4)	2.210(13)
Ru(1)-N(3)	2.138(12)	Ru(1)-C(5)	2.143(14)
Ru(1)-C(1)	2.237(13)	Ru(1)-C(6)	2.179(12)
Ru(1)-C(2)	2.201(12)		
**Bond Angles (º)**			
N(1)-Ru(1)-N(2)	77.8(4)	N(3)-Ru(1)-C(4)	171.8(5)
N(1)-Ru(1)-N(3)	82.9(4)	N(3)-Ru(1)-C(5)	135.1(5)
N(1)-Ru(1)-C(1)	144.0(5)	N(3)-Ru(1)-C(6)	103.4(5)
N(1)-Ru(1)-C(2)	167.7(5)	C(1)-Ru(1)-C(2)	37.5(5)
N(1)-Ru(1)-C(3)	132.4(5)	C(1)-Ru(1)-C(3)	68.3(5)
N(1)-Ru(1)-C(4)	100.6(5)	C(1)-Ru(1)-C(4)	82.1(5)
N(1)-Ru(1)-C(5)	91.3(5)	C(1)-Ru(1)-C(5)	68.6(5)
N(1)-Ru(1)-C(6)	109.2(5)	C(1)-Ru(1)-C(6)	38.1(5)
N(2)-Ru(1)-N(3)	83.3(4)	C(2)-Ru(1)-C(3)	36.8(5)
N(2)-Ru(1)-C(1)	136.8(5)	C(2)-Ru(1)-C(4)	67.1(5)
N(2)-Ru(1)-C(2)	105.1(4)	C(2)-Ru(1)-C(5)	78.6(5)
N(2)-Ru(1)-C(3)	91.0(5)	C(2)-Ru(1)-C(6)	66.7(5)
N(2)-Ru(1)-C(4)	104.6(5)	C(3)-Ru(1)-C(4)	37.5(5)
N(2)-Ru(1)-C(5)	138.9(5)	C(3)-Ru(1)-C(5)	67.3(5)
N(2)-Ru(1)-C(6)	170.7(5)	C(3)-Ru(1)-C(6)	79.7(5)
N(3)-Ru(1)-C(1)	90.8(5)	C(4)-Ru(1)-C(5)	38.1(5)
N(3)-Ru(1)-C(2)	109.2(5)	C(4)-Ru(1)-C(6)	68.5(6)
N(3)-Ru(1)-C(3)	142.3(5)	C(5)-Ru(1)-C(6)	37.5(5)

^{1}H NMR, IR and ESI-MS

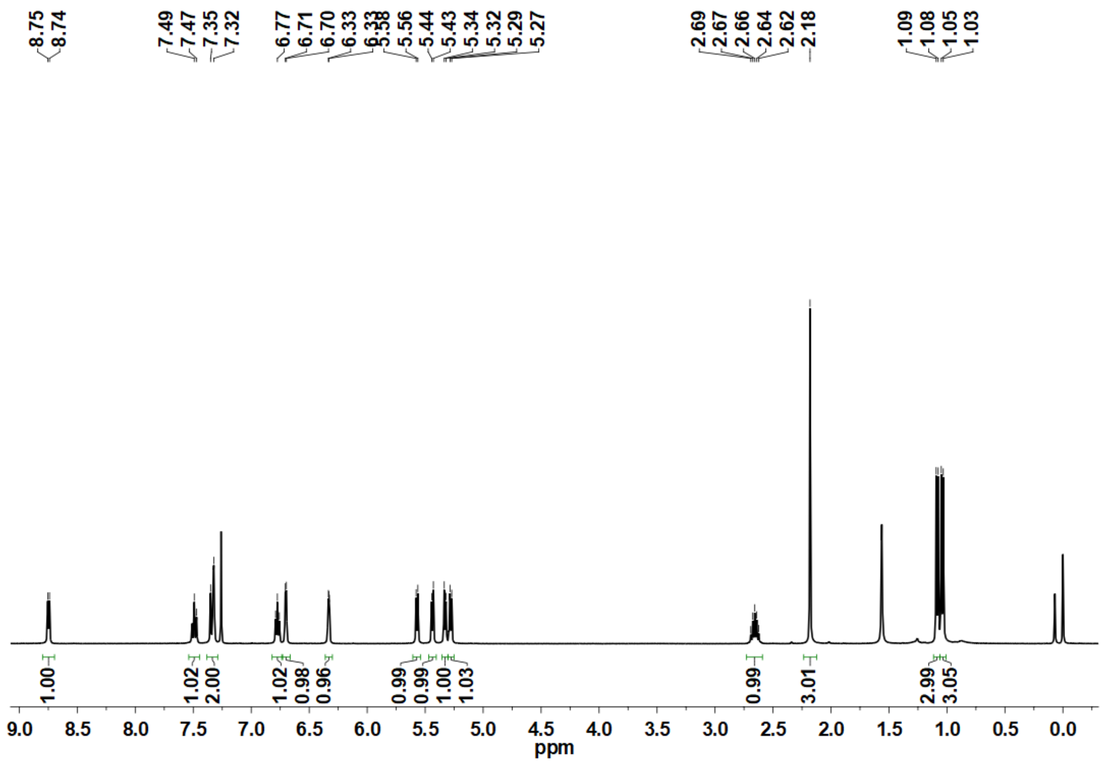

Supplementary Fig. 6 | ^{1}H NMR ($CDCl_3$) spectrum of **1**.

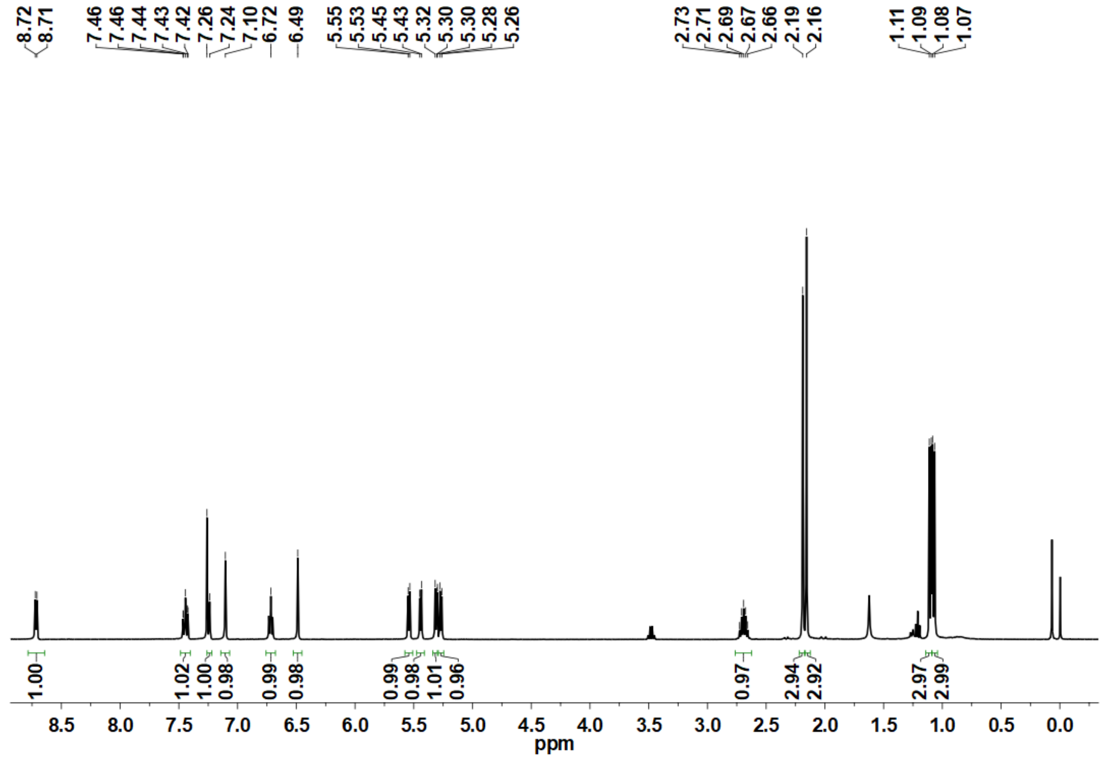

Supplementary Fig. 7 | ^{1}H NMR ($CDCl_3$) spectrum of **2**.

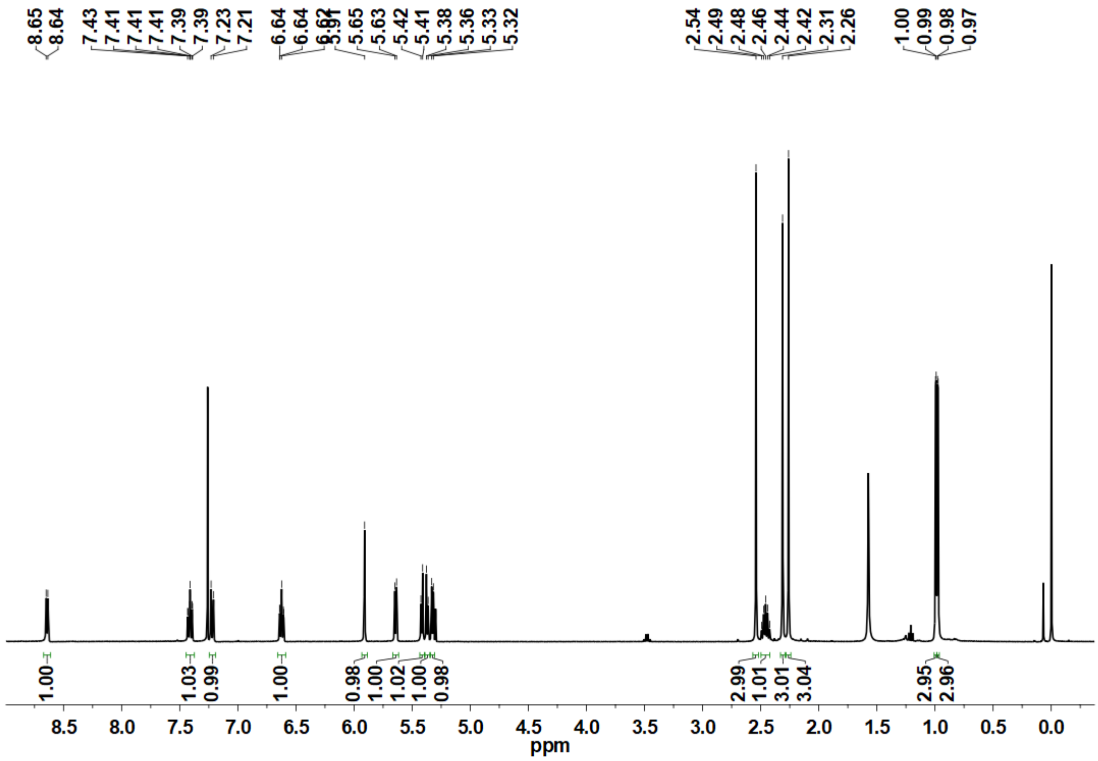

Supplementary Fig. 8 | ^{1}H NMR ($CDCl_3$) spectrum of **3**.

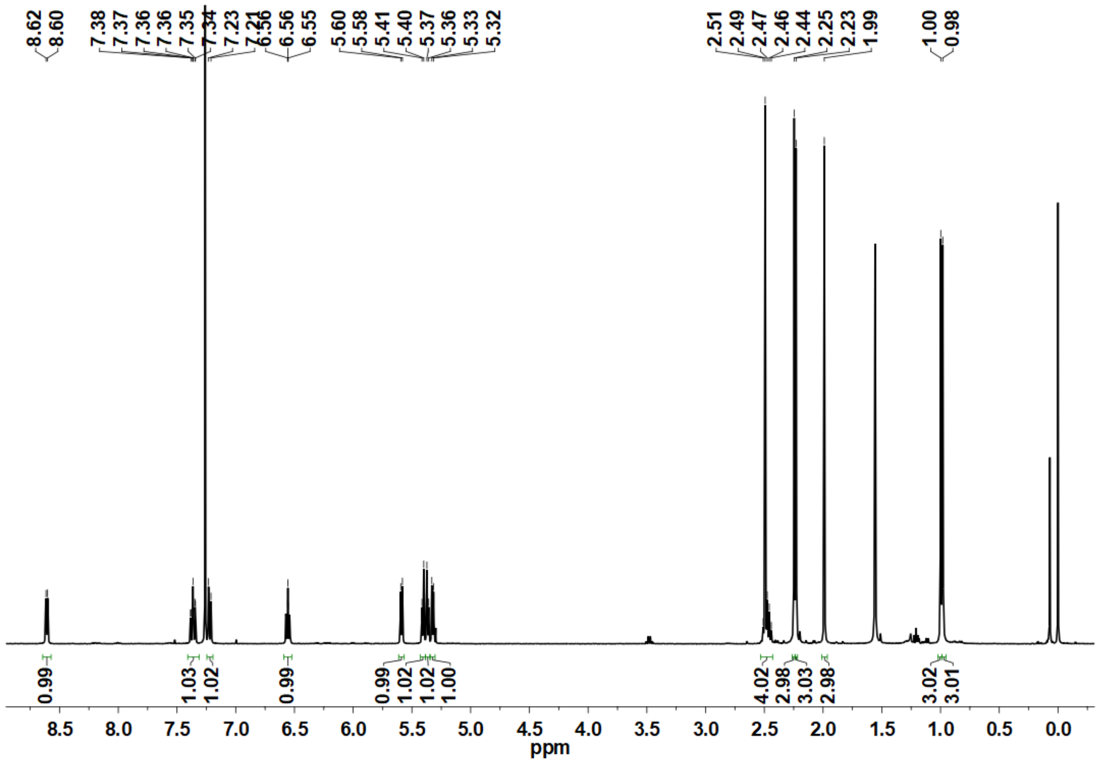

Supplementary Fig. 9 | ^{1}H NMR ($CDCl_3$) spectrum of **4**.

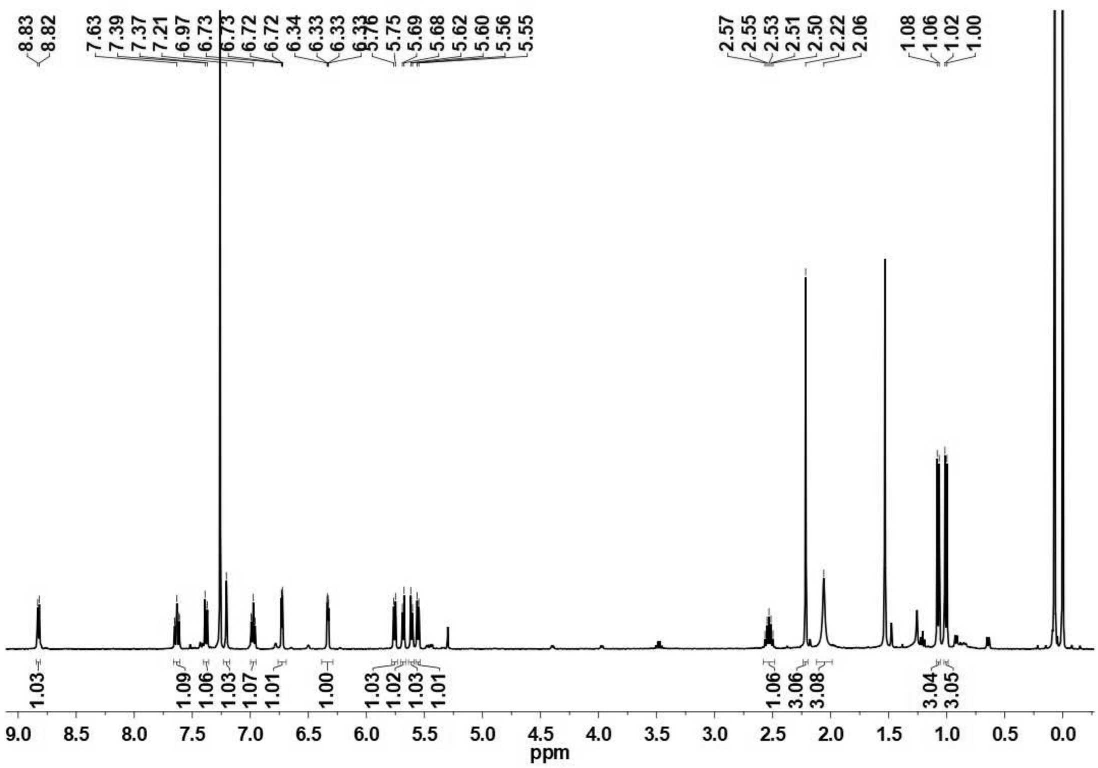

Supplementary Fig. 10 | ^{1}H NMR ($CDCl_3$) spectrum of **[1-NH$_3$]PF$_6$**.

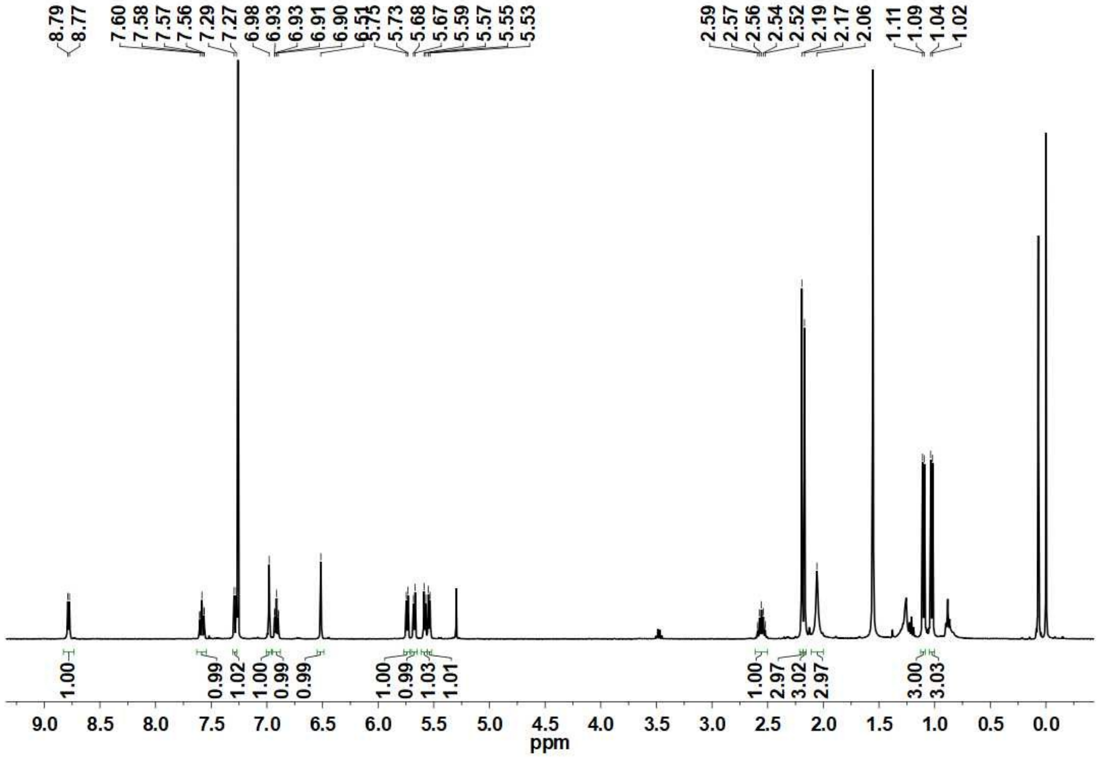

Supplementary Fig. 11 | ^{1}H NMR ($CDCl_3$) spectrum of **[2-NH$_3$]PF$_6$**.

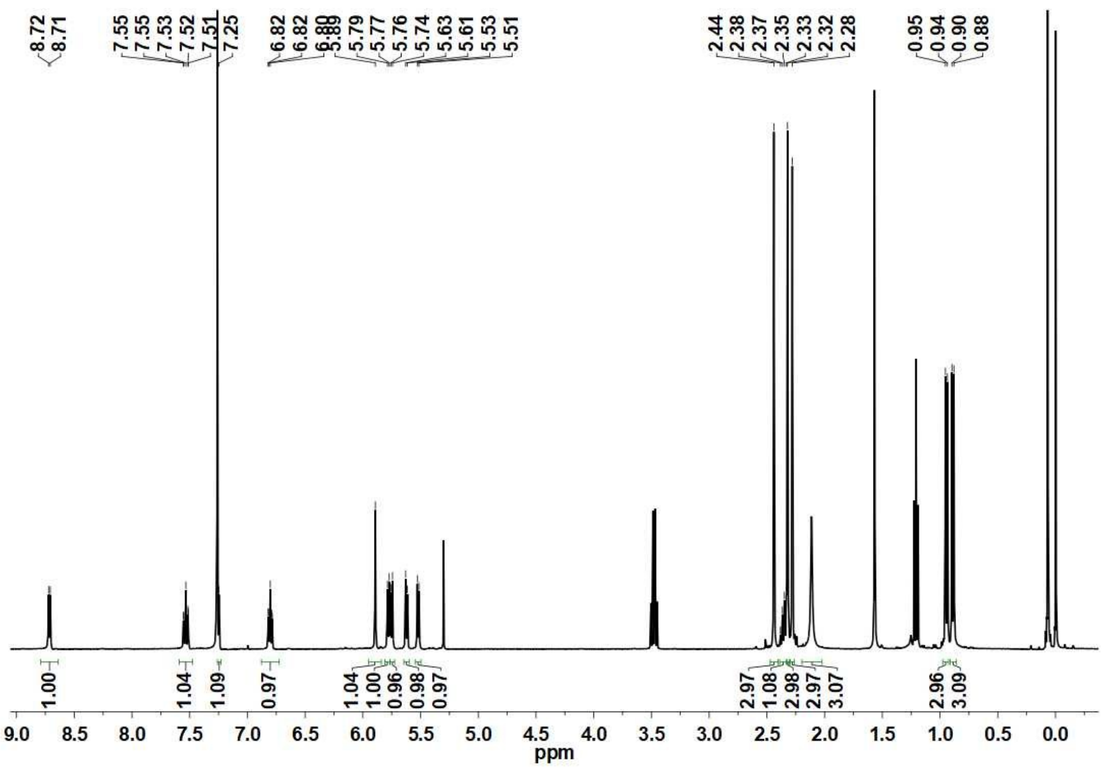

Supplementary Fig. 12 | ^{1}H NMR ($CDCl_3$) spectrum of **[3-NH$_3$]PF$_6$**.

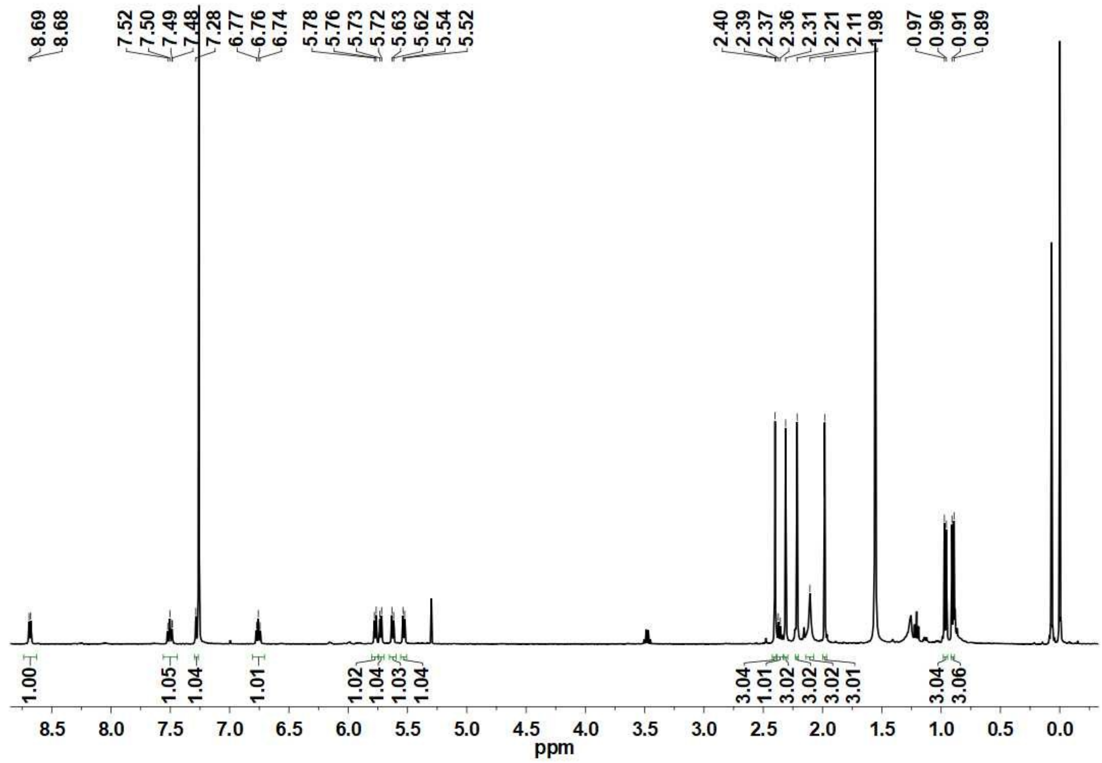

Supplementary Fig. 13 | ^{1}H NMR ($CDCl_3$) spectrum of **[4-NH$_3$]PF$_6$**.

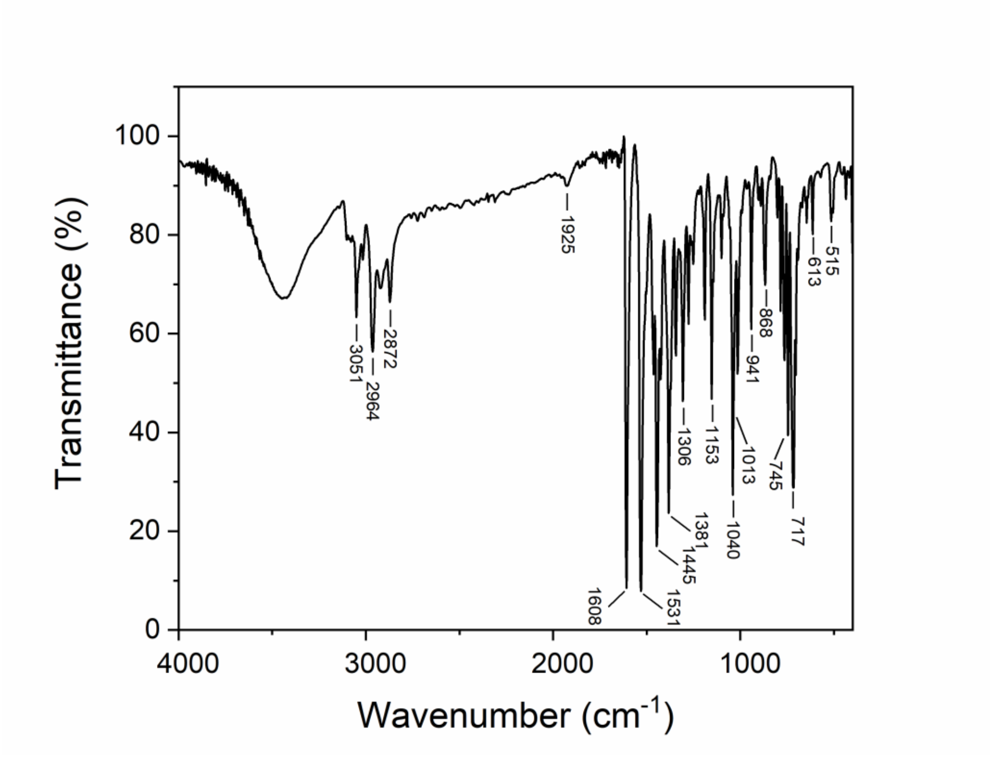

Supplementary Fig. 14 | IR spectrum of **1**.

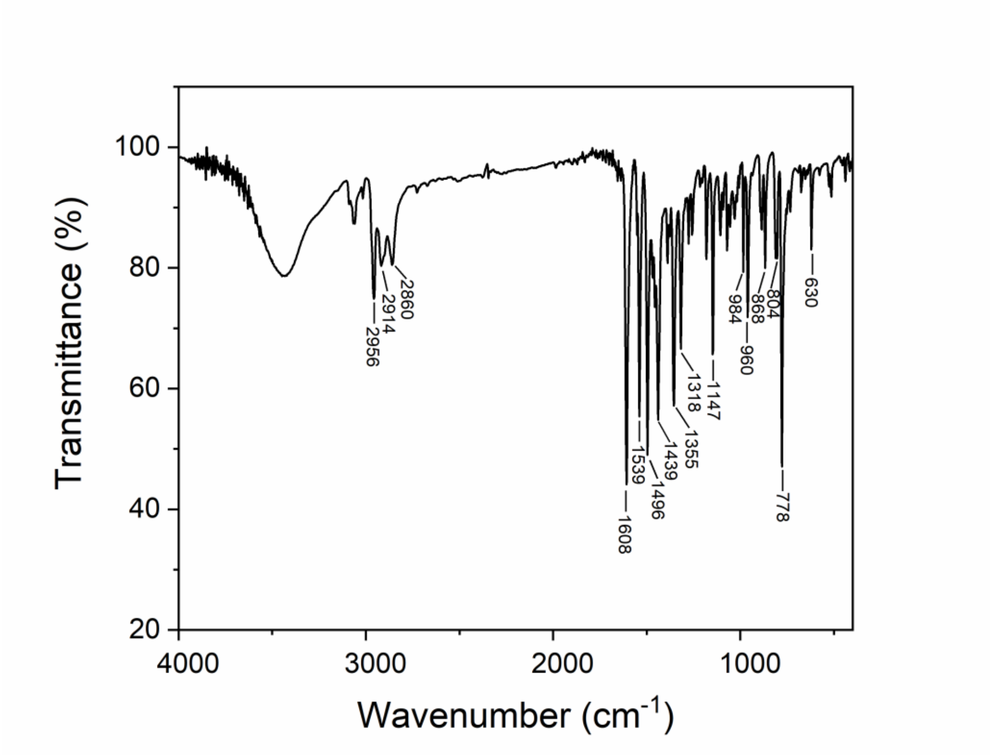

Supplementary Fig. 15 | IR spectrum of **2**.

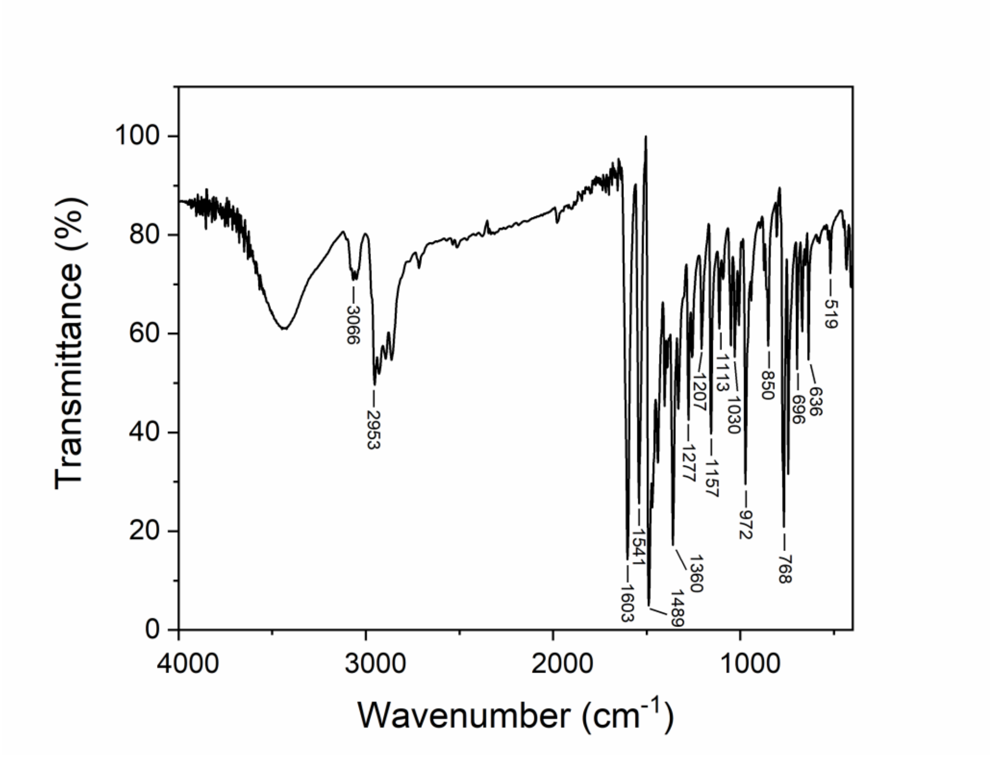

Supplementary Fig. 16 | IR spectrum of **3**.

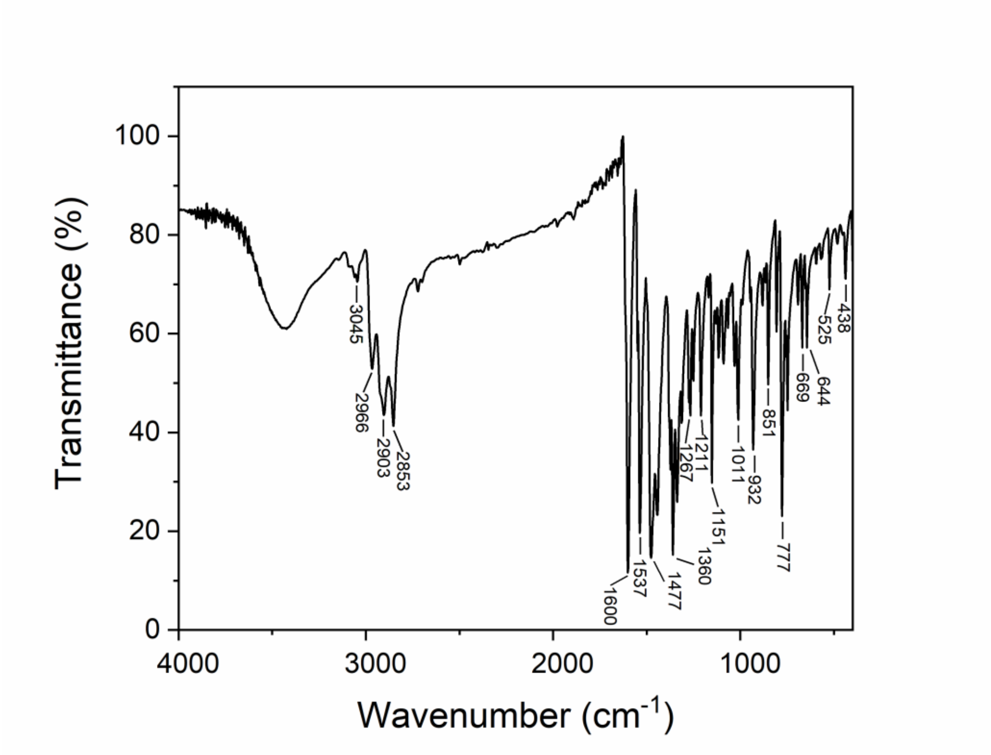

Supplementary Fig. 17 | IR spectrum of **4**.

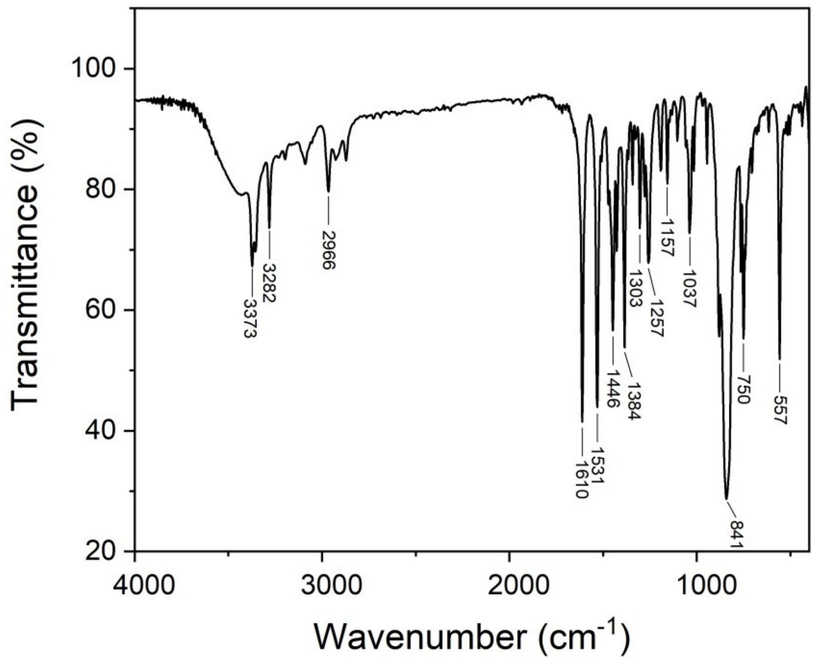

Supplementary Fig. 18 | IR spectrum of **[1-NH$_3$]PF$_6$**.

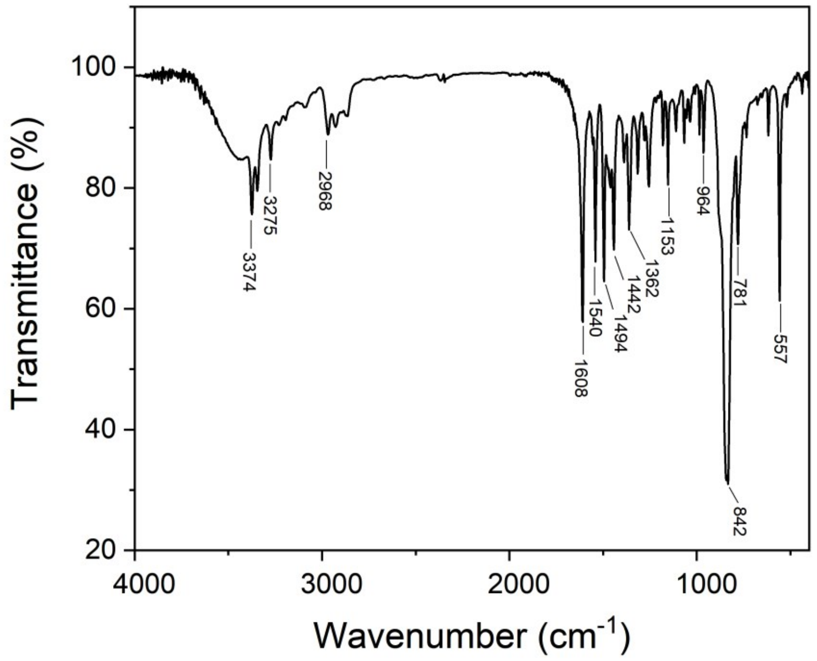

Supplementary Fig. 19 | IR spectrum of **[2-NH$_3$]PF$_6$**.

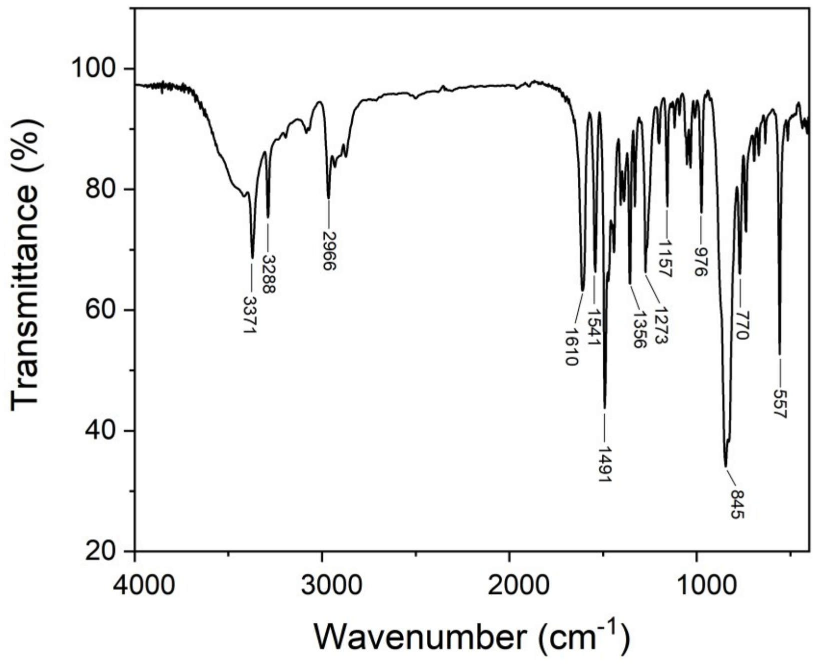

Supplementary Fig. 20 | IR spectrum of **[3-NH_3]PF_6**.

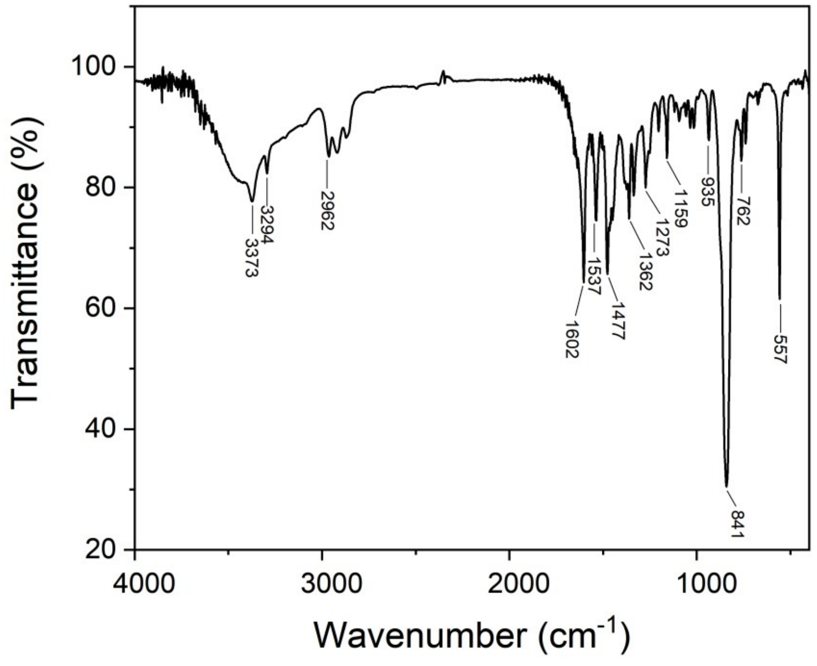

Supplementary Fig. 21 | IR spectrum of **[4-NH_3]PF_6**.

.

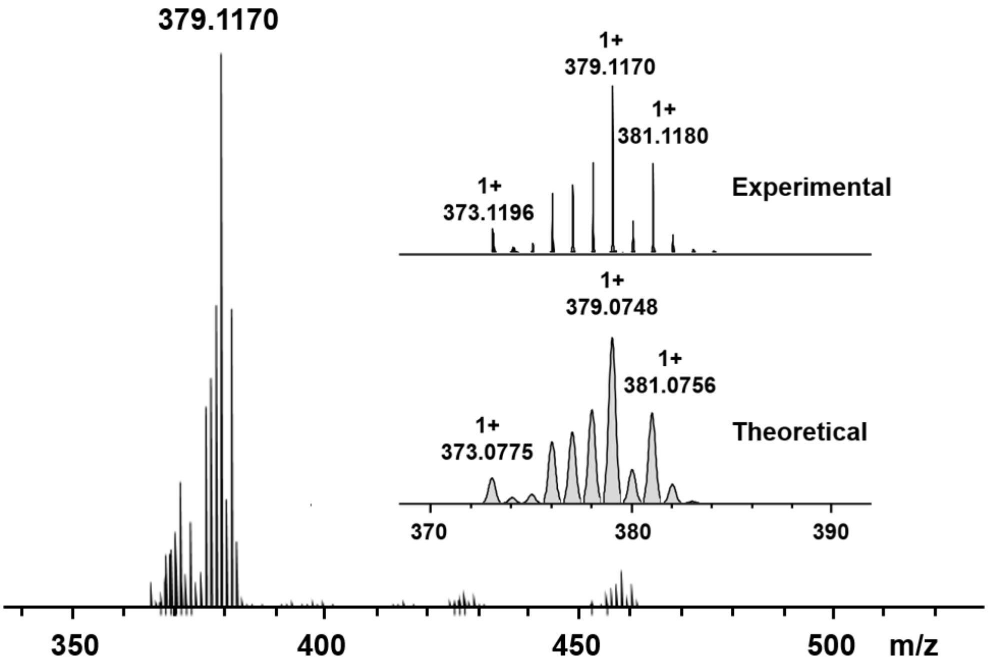

Supplementary Fig. 22 | ESI-MS spectrum of **1**.

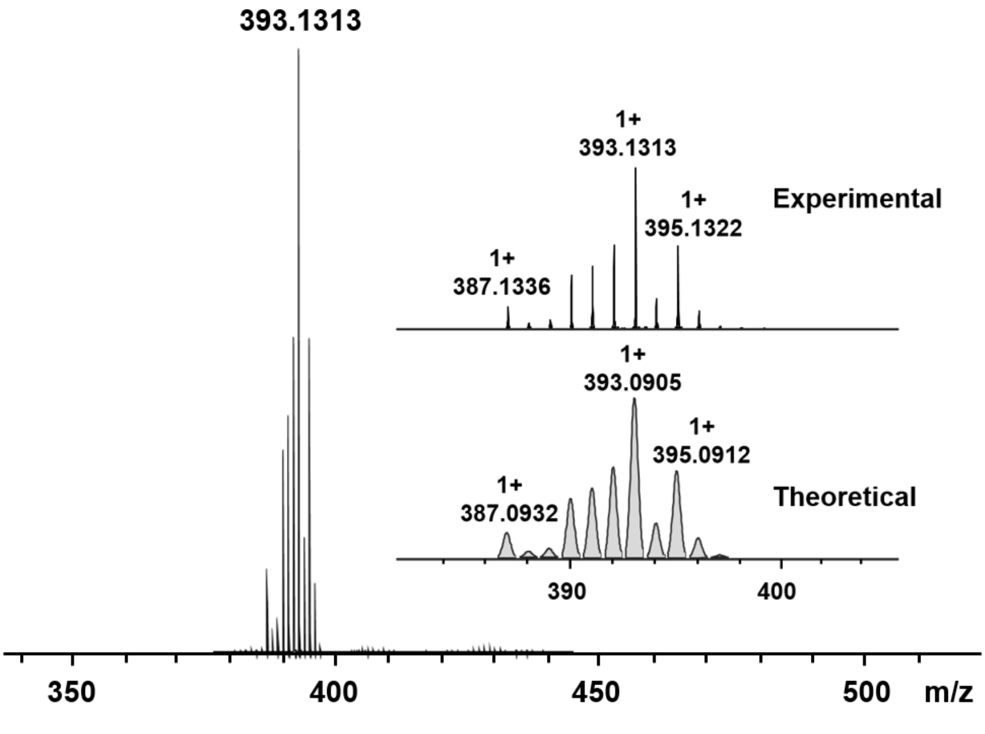

Supplementary Fig. 23 | ESI-MS spectrum of **2**.

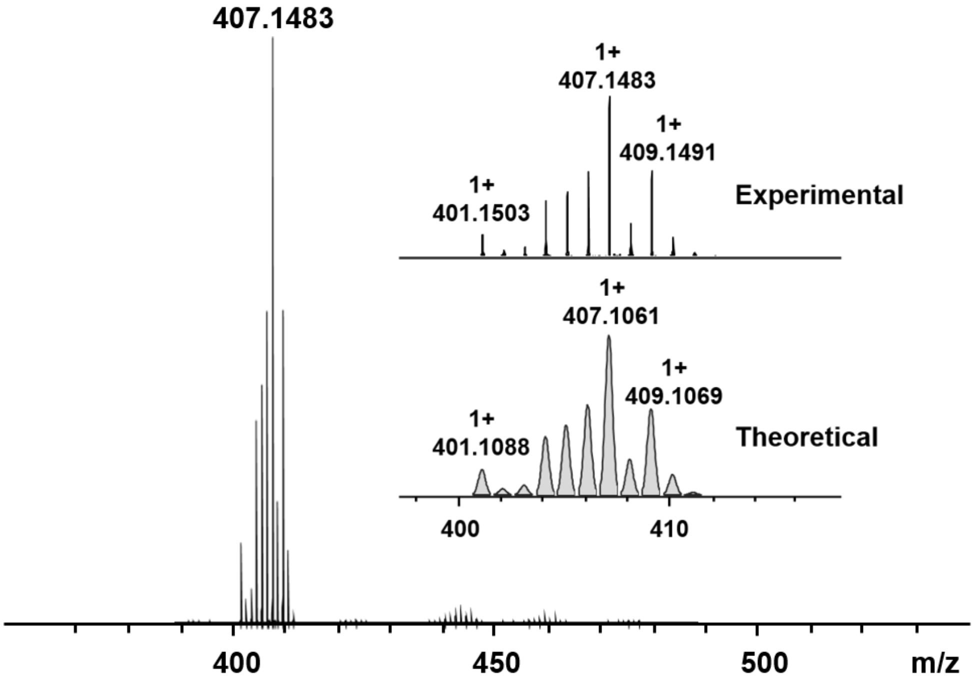

Supplementary Fig. 24 | ESI-MS spectrum of **3**.

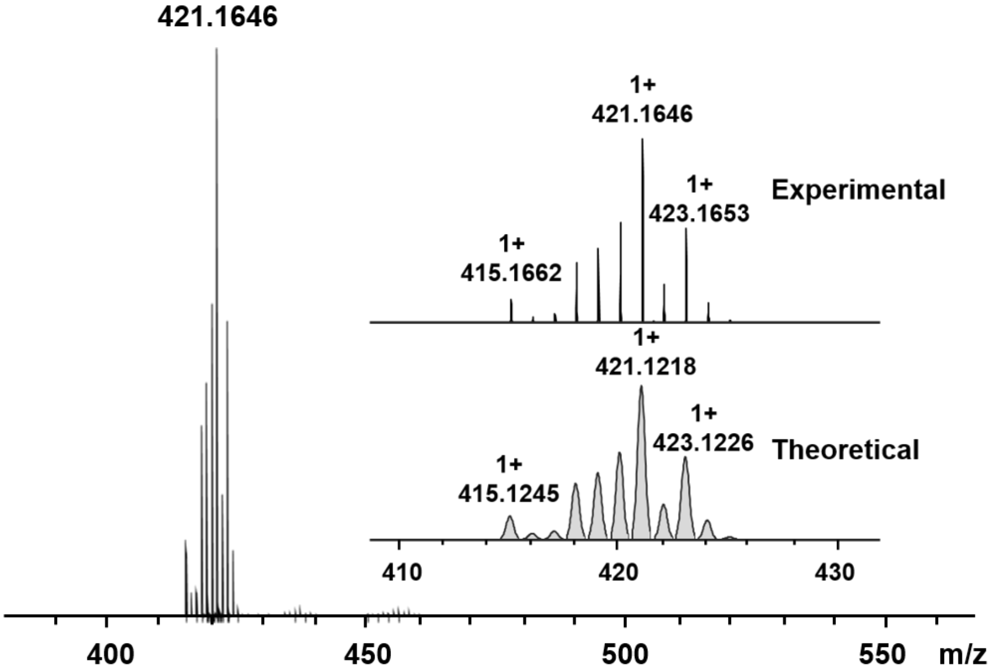

Supplementary Fig. 25 | ESI-MS spectrum of **4**.

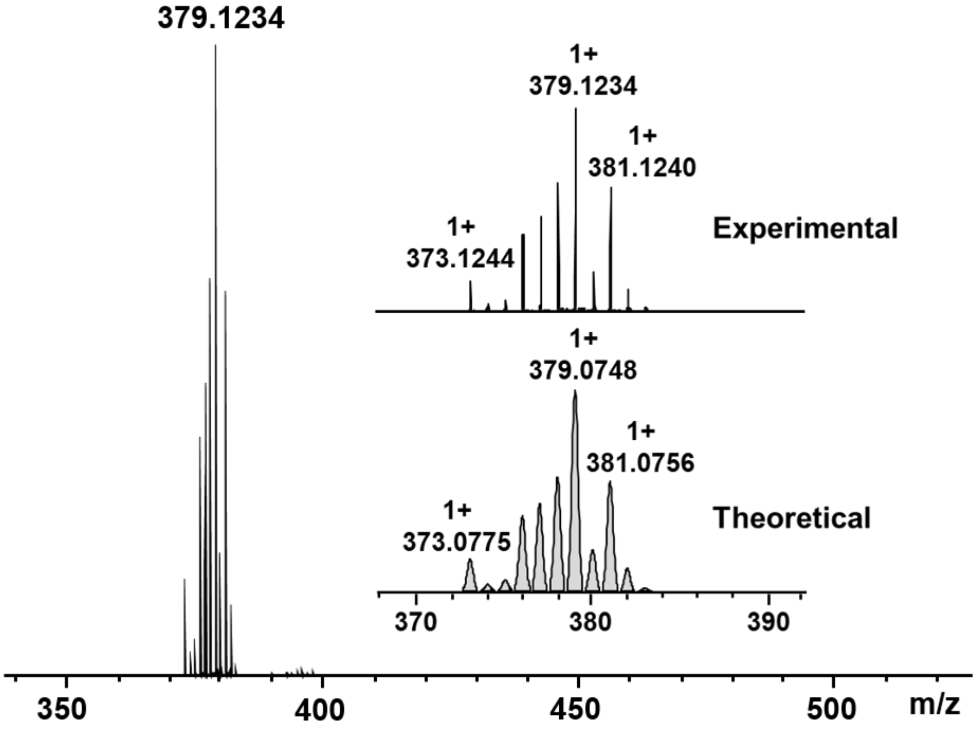

Supplementary Fig. 26 | ESI-MS spectrum of **[1-NH$_3$]$^+$**.

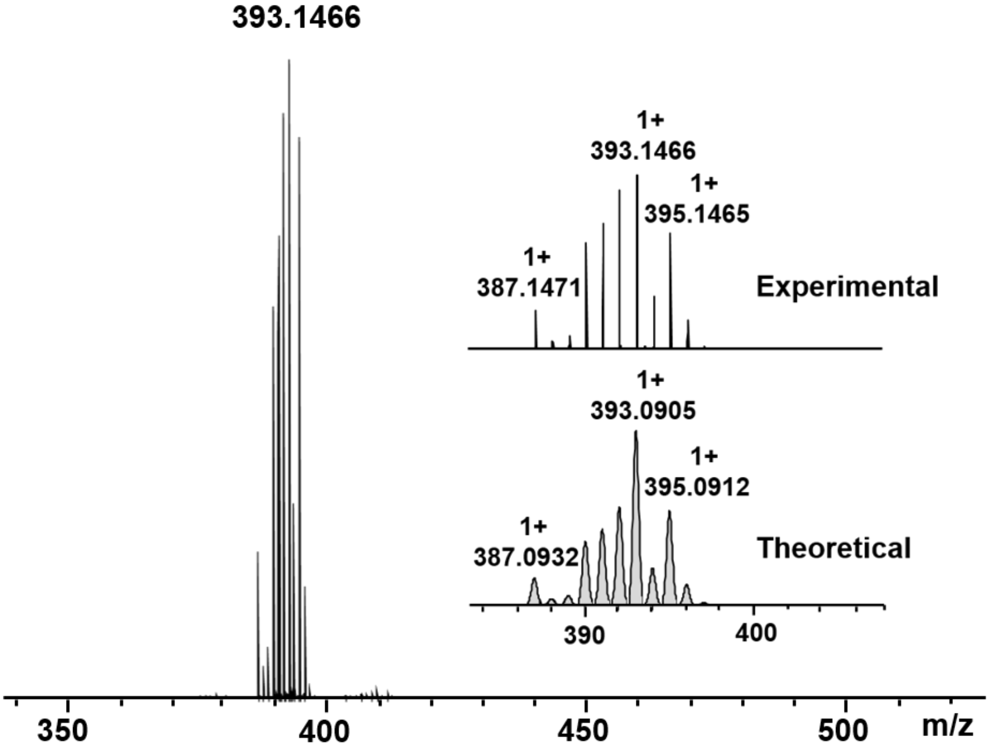

Supplementary Fig. 27 | ESI-MS spectrum of **[2-NH$_3$]$^+$**.

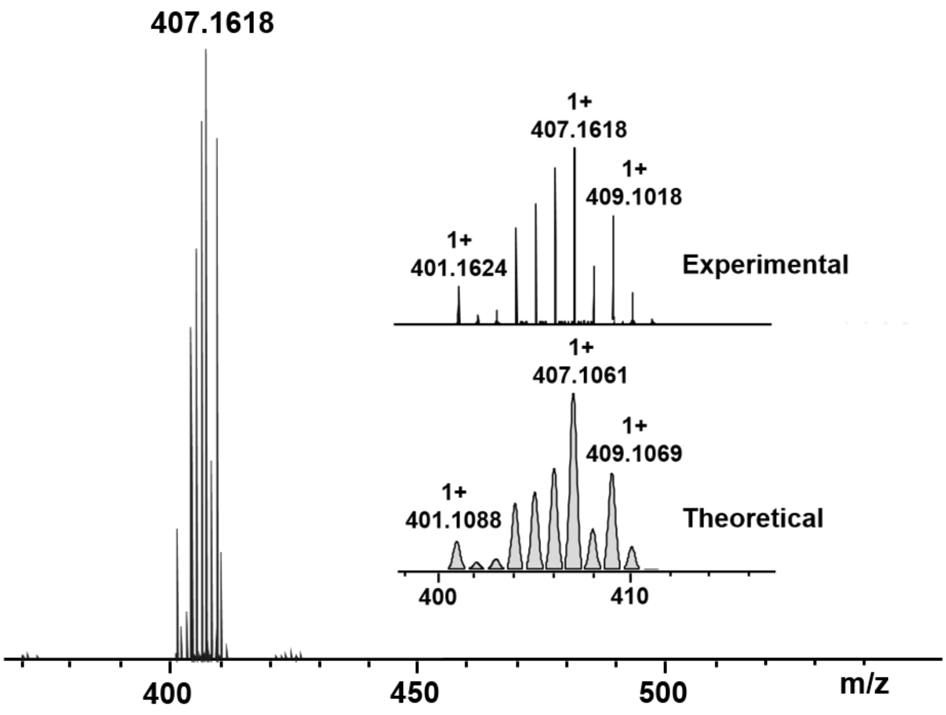

Supplementary Fig. 28 | ESI-MS spectrum of **[3-NH$_3$]$^+$**.

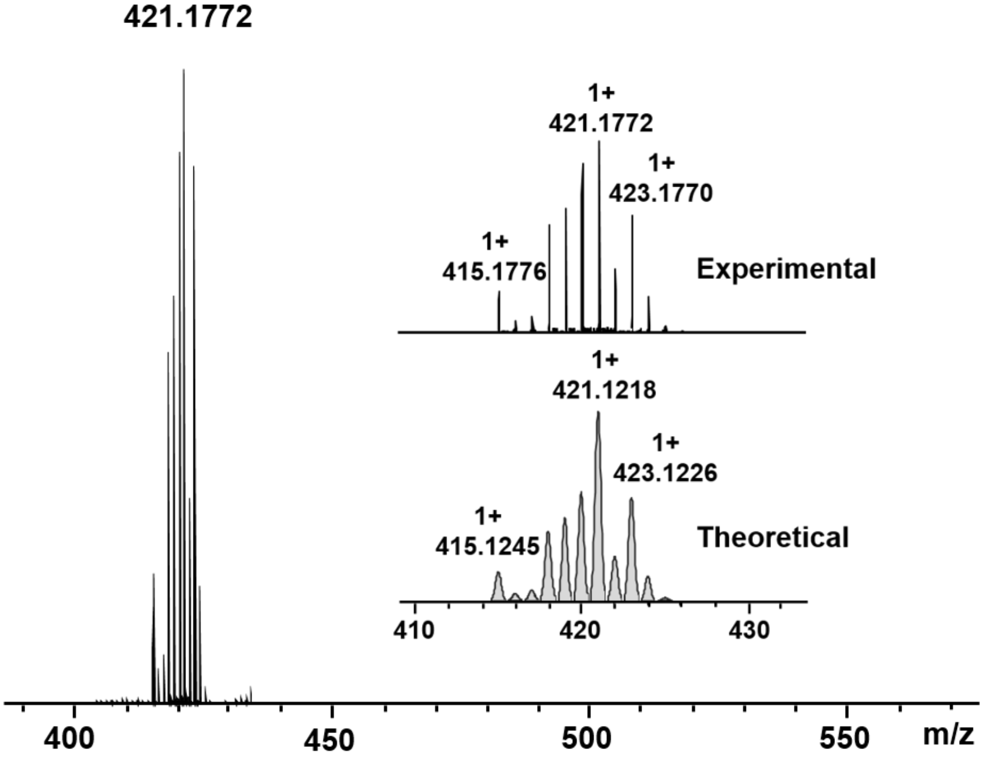

Supplementary Fig. 29 | ESI-MS spectrum of **[4-NH$_3$]$^+$**.

3. Electrochemical studies

CV

The typical sealed three-electrode cell was employed with AgCl/Ag electrode in saturated KCl solution, a Pt wire, and a glassy carbon electrode with a diameter of 5 mm as the reference electrode, counter electrode, and working electrode, respectively. Unless mentioned otherwise, the cyclic voltammetry (CV) and differential pulse voltammetry (DPV) were measured using 1 mM Ru complex in MeCN and 0.1M Bu_4NPF_6 was used as supporting electrolyte. In the CV experiments with added ammonia, the ratio of NH_3/NH_4PF_6 is 1:1.

Conversion for redox potentials measured *vs.* Ag/AgCl electrodes at 25 °C using $Cp_2Fe^{+/0}$ redox couples as an internal standard. Conversion for $E_{\text{measured value}}$ *vs.* Ag/AgCl to E *vs*. $Cp_2Fe^{+/0}$ in MeCN is based on the eqn. S1.

$$\textbf{E (V } \textit{vs.} \textbf{ Cp}_2\textbf{Fe}^{+/0} \textbf{ in CH}_3\textbf{CN) = E}_{\text{measured value}} \textbf{ (V } \textit{vs.} \textbf{ Ag/AgCl) − 0.43} \qquad \textbf{(eqn. S1)}$$

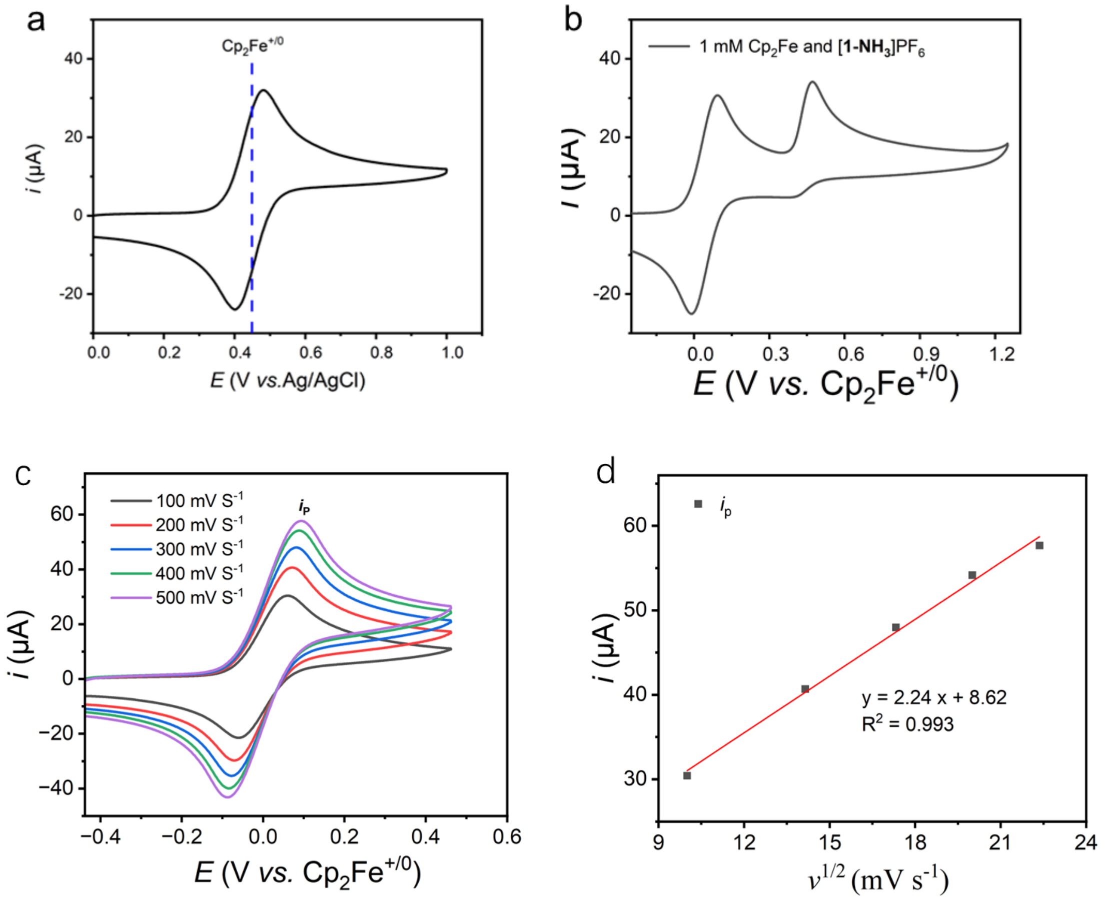

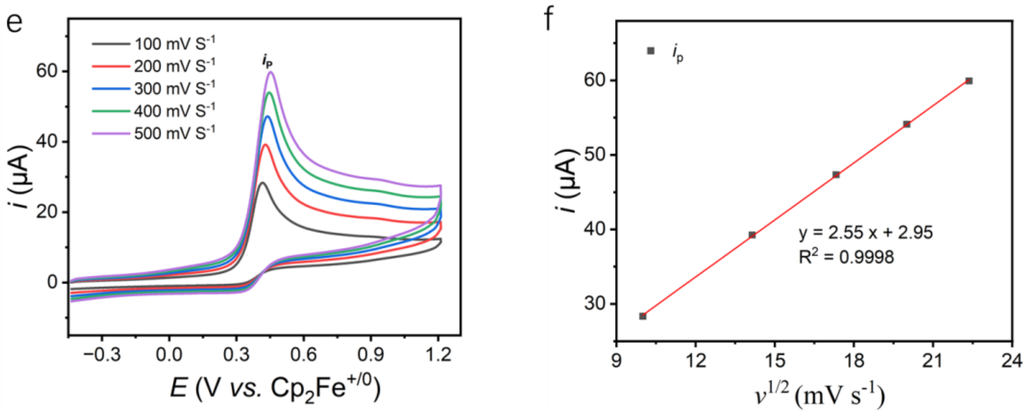

Supplementary Fig. 30 | (a) The CV of ferrocene as internal standard in MeCN solution with scan rate at 100 mV s^{-1}; (b) The CV of ferrocene and [**1-NH$_3$**]PF_6 in MeCN solution with scan rate at 100 mV s^{-1}; (c) CVs of ferrocene (1 mM) solution in MeCN with scan rate at 100, 200, 300, 400, 500 mV s^{-1}; (d) Plot of i_p *vs*. $v^{1/2}$; (e) CVs of [**1-NH$_3$**]PF_6 (1 mM) solution in MeCN with scan rate at 100, 200, 300, 400, 500 mV s^{-1}; (f) Plot of i_p *vs*. $v^{1/2}$;

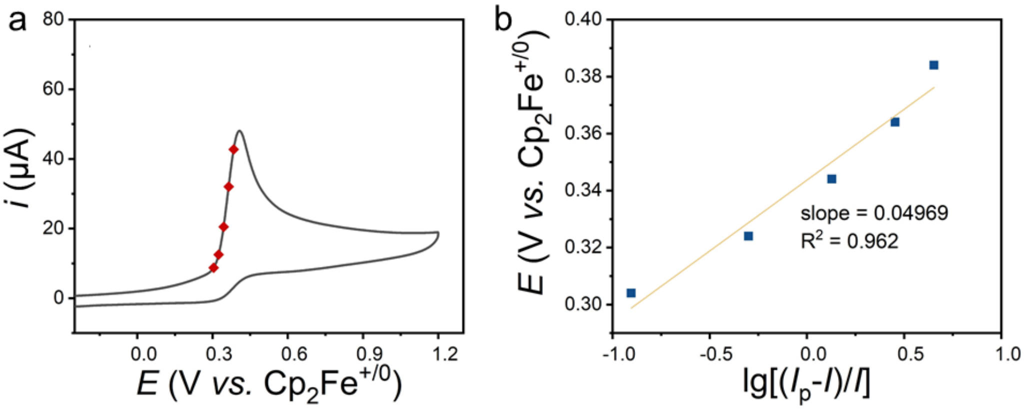

Supplementary Fig. 31 | (a) The CV of [**1-NH$_3$**]PF_6 in MeCN solution with scan rate at 100 mV s^{-1}. (b) Plot of *E vs*. lg[(I_p-I)/I].

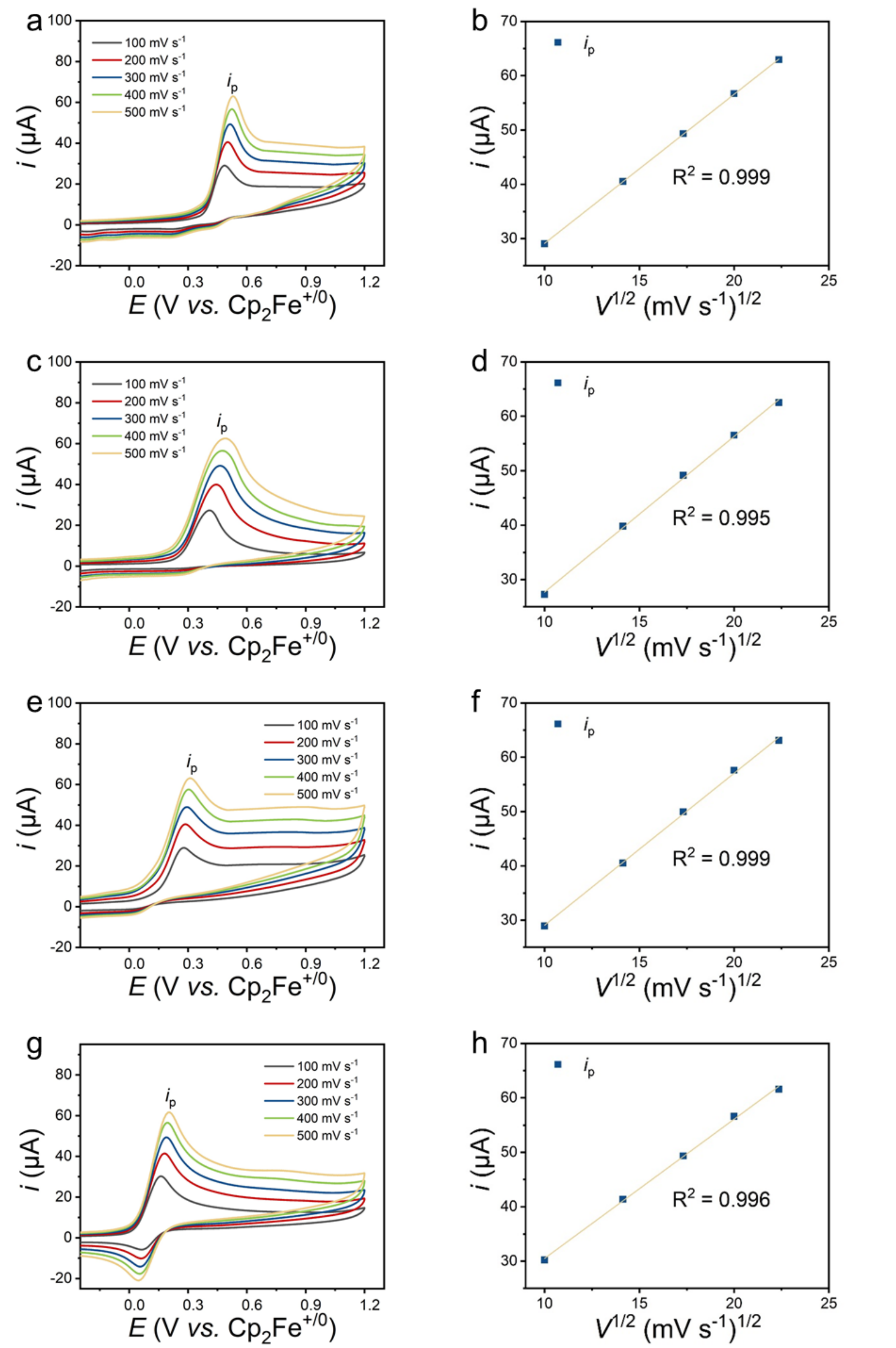

Supplementary Fig. 32 | CVs of (a) **[1-NH_3]PF_6** (1 mM), (c) **[2-NH_3]PF_6** (1 mM), (e) **[3-NH_3]PF_6** (1 mM), (g) **[4-NH_3]PF_6** (1 mM) solution in MeCN with scan rate at 100, 200, 300, 400, 500 mV s^{-1}; (b, d, f) Plot of i_p *vs.* $v^{1/2}$.

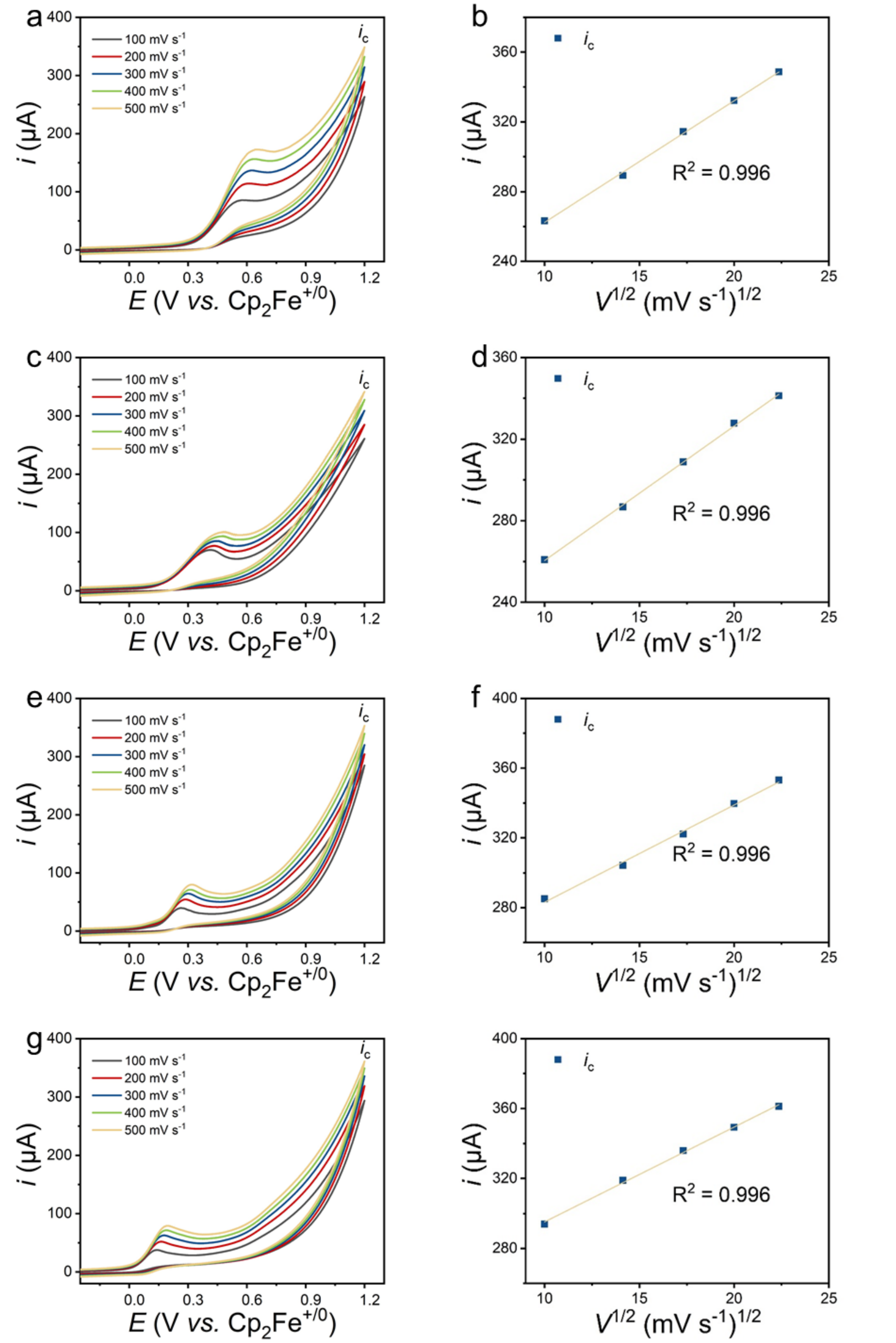

Supplementary Fig. 33 | CVs of complex (a) **[1-NH$_3$]PF$_6$** (1 mM), (c) **[2-NH$_3$]PF$_6$** (1 mM), (e) **[3-NH$_3$]PF$_6$** (1 mM), (g) **[4-NH$_3$]PF$_6$** (1 mM) in presence of 0.05 M NH_3/NH_4PF_6 in MeCN with scan rate at 100, 200, 300, 400, 500 mV s^{-1}; (b, d, f, h) Plot of i_c *vs.* $v^{1/2}$.

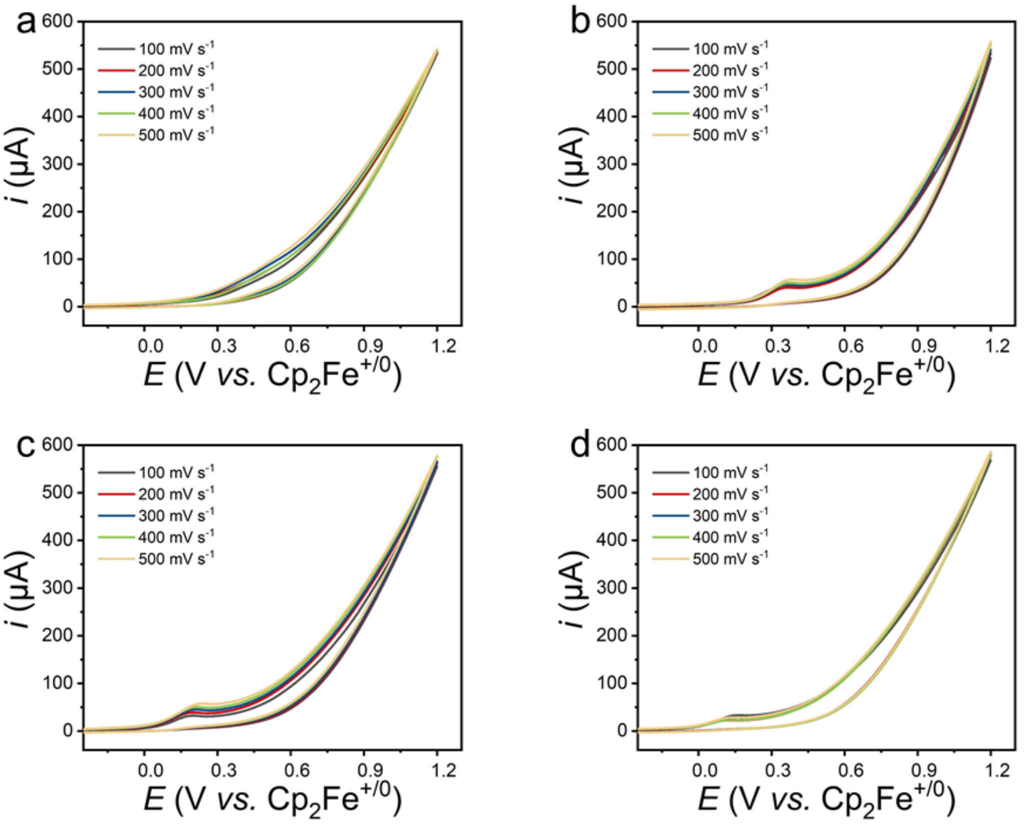

Supplementary Fig. 34 | CVs of complex (a) **[1-NH$_3$]PF$_6$** (1 mM), (b) **[2-NH$_3$]PF$_6$** (1 mM), (c) **[3-NH$_3$]PF$_6$** (1 mM), (d) **[4-NH$_3$]PF$_6$** (1 mM) in presence of 1.0 M NH_3/NH_4PF_6 in MeCN with scan rate at 100, 200, 300, 400, 500 mV s^{-1}.

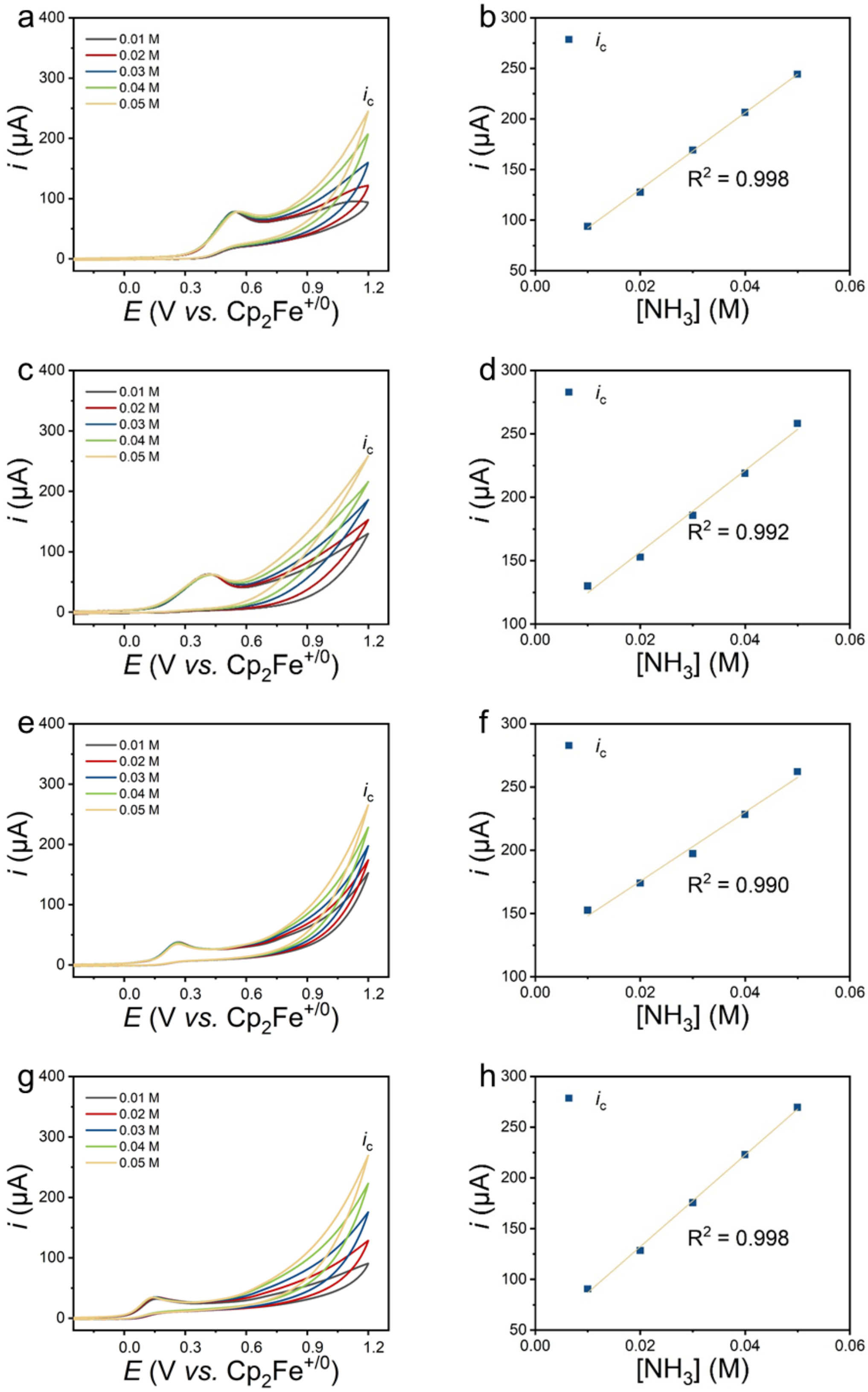

Supplementary Fig. 35 | CVs of complex (a) **[1-NH_3]PF_6** (1 mM), (c) **[2-NH_3]PF_6** (1 mM), (e) **[3-NH_3]PF_6** (1 mM), (g) **[4-NH_3]PF_6** (1 mM) in presence of NH_3/NH_4PF_6 with various concentration (0.01 M, 0.02 M, 0.03 M, 0.04 M, 0.05 M) in MeCN with scan rate at 100 mV s^{-1}; (b, d, f, h) Plot of i_c vs. [NH_3].

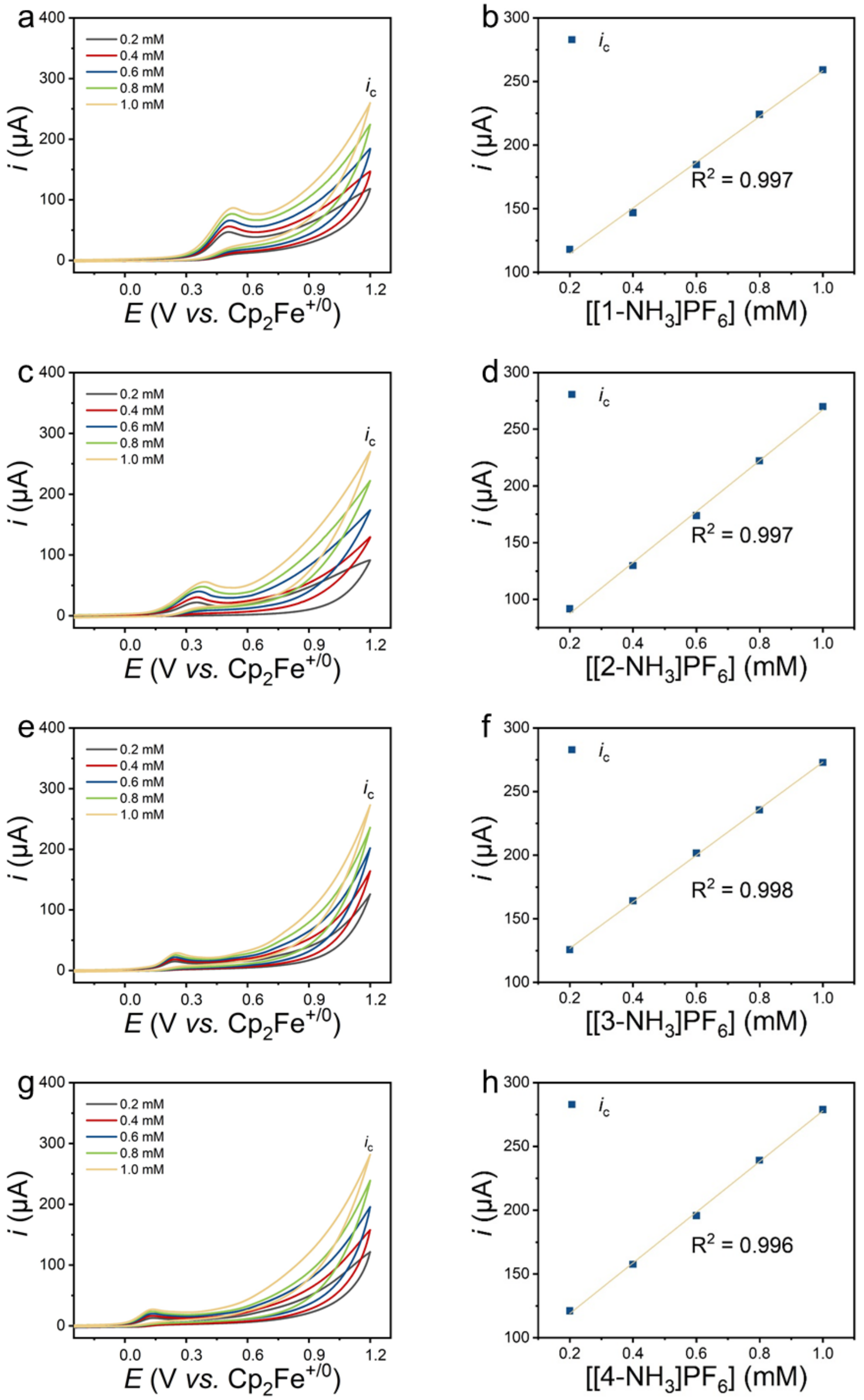

Supplementary Fig. 36 | CVs of complex (a) **[1-NH_3]PF_6**, (c) **[2-NH_3]PF_6**, (e) **[3-NH_3]PF_6**, (g) **[4-NH_3]PF_6** with various concentration (0.2, 0.4, 0.6, 0.8, 1.0 mM) in presence of 0.05 M NH_3/NH_4PF_6 with scan rate at 100 mV s^{-1}; (b, d, f, h) Plot of i_c vs. [[1-NH_3]PF_6], [[2-NH_3]PF_6], [[3-NH_3]PF_6], [[4-NH_3]PF_6].

CPC experiment

Controlled potential coulometry (CPC) measurements.

Carbon cloth (A = 1 cm^2), platinum plate (A = 1 cm^2), AgCl/Ag electrode in saturated KCl solution were used as the working electrode, counter electrode, and reference electrode, respectively. Unless mentioned otherwise, The CPC experiments were performed in a sealed electrolytic cell (270 mL) under an Ar atmosphere at a given potential with catalyst (0.01 mM) and saturated NH_3 solution (2.0 M) in MeCN. The total volume of the solution in the electrolysis experiments was 80 mL, and the headspace volume was 190 mL. The gas sampling volume is 0.1 mL. The setup used for the electrolysis experiments is shown in Figure 37.

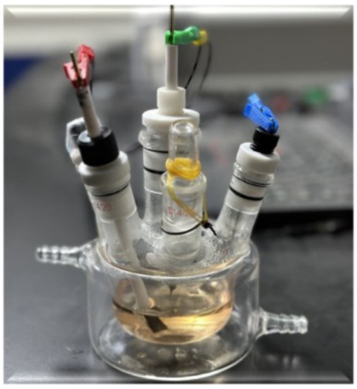

Supplementary Fig. 37 | Electrolysis experimental setup diagram.

The selectivity of N_2H_4 formation can be calculated using the following eqn. S2:

$$\textbf{Selectivity of } N_2H_4 \textbf{ formation} = n_{N2H4}/(n_{N2} + n_{N2H4}) \times 100\% \text{ (eqn. S2)}$$

nN_2H_4 and nN_2 are the mol of N_2H_4 and N_2 (mol) after electrolysis, respectively.

The FE of the catalyst can be calculated using the following eqn. S3[13]:

$$FE = n_{cat}mF/it \times 100\% \text{ (eqn. S3)}$$

where n_{cat} is the number of electrons involved in ammonia oxidation (2 e), m is the mol of H_2 or N_2H_4 (mol), F is the Faraday constant, i is the current (A) and t is the electrolysis time (s).

Supplementary Table 7 | The electrocatalytic performances of background.

Entry	Cat.	E_{app}[c]	Time(h)	n_{H2}(μmol)	n_{N2H4}(μmol)	n_{N2}(μmol)
1[a]	-	0.39	24	trace	trace	trace
2[a]	-	0.8	2	22.8	21.6	0.5
3[b]	**rinsed carbon cloth**	0.8	2	22.2	21.4	0.4

[a] Conditions: a Ag/AgCl electrode in saturated KCl solution as reference electrode, a platinum plate (1 cm^2) as counter electrode, a carbon cloth (1 cm^2) as working electrode, $[NH_3]$ = 2.0 M, [cat.] = 0. [b] using carbon cloth which is rinsed with ultradry acetonitrile after CPC experiments with catalyst **[4-NH$_3$]PF$_6$** as working electrode.

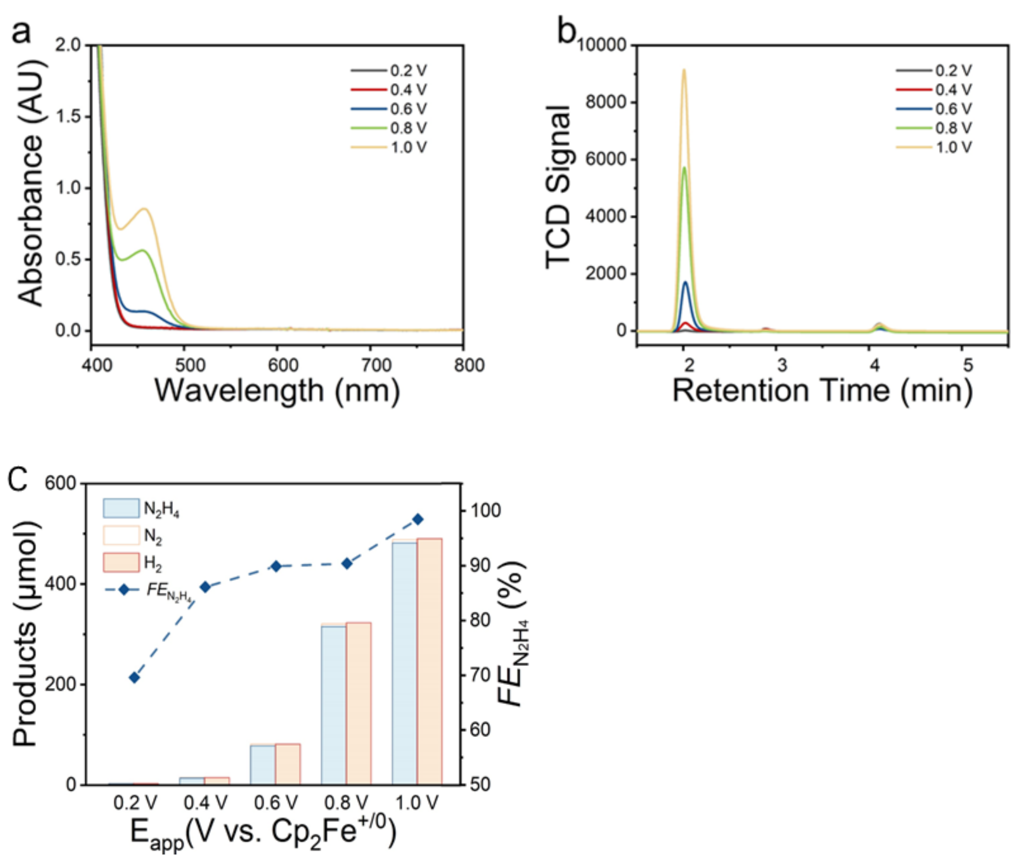

Supplementary Fig. 38 | Determination of (a) N_2H_4 in the electrolyte and (b) GC-TCD trace (c) FE_{N2H4} and molar amount of H_2, O_2 and N_2 after the CPC experiment of NH_3 solution (2.0 M) in MeCN containing **[2-NH$_3$]PF$_6$** (0.01 mM) at an applied potential of 0.2, 0.4, 0.6, 0.8, 1.0 V *vs.* $Cp_2Fe^{+/0}$ for 2 h.

Supplementary Table 8 | The electrocatalytic performances of complex **[2-NH$_3$]PF$_6$** with various potential (0.2, 0.4, 0.6, 0.8, 1.0 V *vs.* $Cp_2Fe^{+/0}$) for 2 h.[a]

Entry	Cat.	E_{app}	Time (h)	TON_{H2}/ **n_{H2}(μmol)**	TOF_{H2} (h^{-1})	TON_{N2H4}/ **n_{N2H4}(μmol)**	TOF_{N2H4} (h^{-1})	TON_{N2}/ **n_{N2}(μmol)**	TOF_{N2} (h^{-1})	FE_{N2H4} (%)	S_{N2H4} (%)
1	**[2-NH$_3$]PF$_6$**	0.2	2	3.5<br>**2.8**	1.8	3.4<br>**2.7**	1.7	Trace	Trace	69.6	100
2	**[2-NH$_3$]PF$_6$**	0.4	2	18.4<br>**14.7**	9.2	17.1<br>**13.7**	8.6	0.5<br>**0.4**	0.3	86.1	97.1
3	**[2-NH$_3$]PF$_6$**	0.6	2	101.8<br>**81.4**	50.9	97.4<br>**77.9**	48.7	1.3<br>**1.1**	0.7	89.9	98.7
4	**[2-NH$_3$]PF$_6$**	0.8	2	403.5<br>**322.8**	201.8	393.9<br>**315.2**	197.0	2.2<br>**1.8**	1.1	90.8	99.4
5	**[2-NH$_3$]PF$_6$**	1.0	2	613.0<br>**490.4**	306.5	602.3<br>**481.8**	301.2	4.9<br>**3.9**	2.5	98.5	99.2

[a] Conditions: a Ag/AgCl electrode in saturated KCl solution as reference electrode, a platinum plate (1 cm^2) as counter electrode, a carbon cloth (1 cm^2) as working electrode, [NH_3] = 2.0 M, [cat.] = 0.01 Mm.

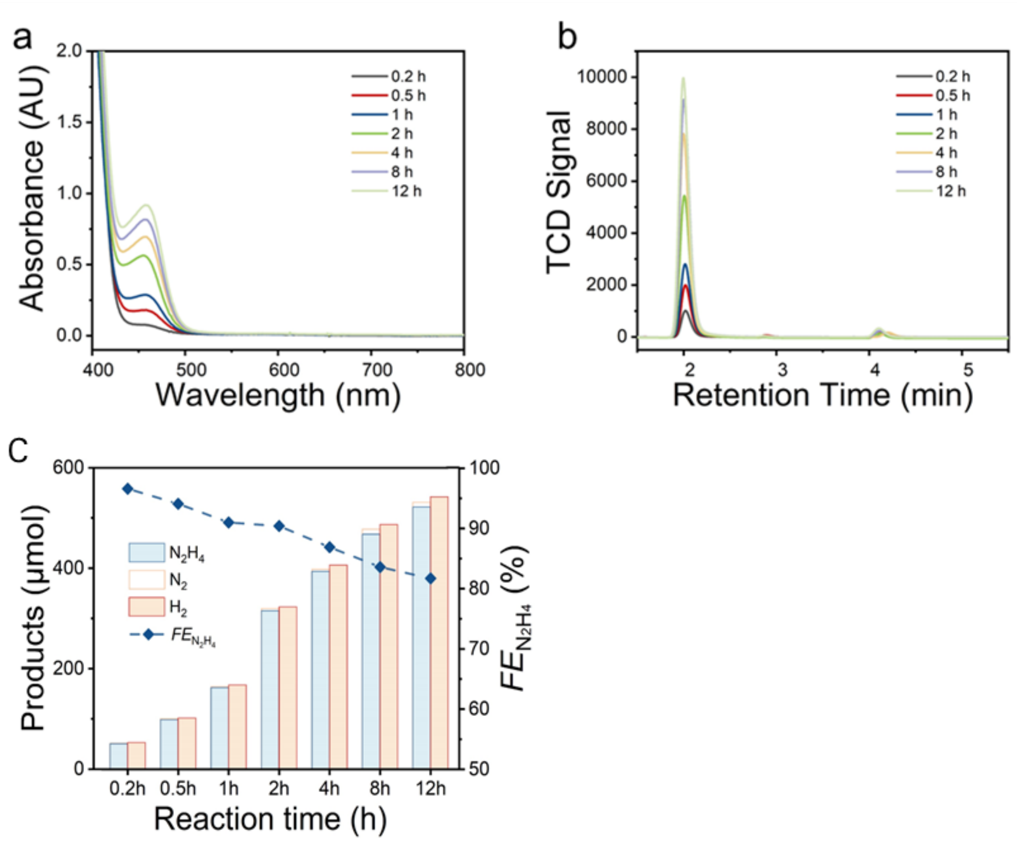

Supplementary Fig. 39 | Determination of (a) N_2H_4 in the electrolyte, (b) GC-TCD trace and (c) FE_{N2H4} and molar amount of H_2, O_2 and N_2 after the CPC experiment of NH_3 solution (2.00 M) in MeCN containing **[2-NH$_3$]PF$_6$** (0.01 mM) at an applied potential of 0.8 V *vs.* $Cp_2Fe^{+/0}$ for 0.2, 0.5, 1, 2, 4, 8, 12 h.

Supplementary Table 9 | The electrocatalytic performances of complex **[2-NH$_3$]PF$_6$** at an applied potential of 0.8 V *vs.* $Cp_2Fe^{+/0}$ for 0.2, 0.5, 1, 2, 4, 8, 12 h.[a]

Entry	Cat.	E_{app}	Time (h)	TON_{H2}/ ***n*$_{H2}$(μmol)**	TOF_{H2} (h^{-1})	TON_{N2H4}/ ***n*$_{N2H4}$(μmol)**	TOF_{N2H4} (h^{-1})	TON_{N2}/ ***n*$_{N2}$(μmol)**	TOF_{N2} (h^{-1})	FE_{N2H4} (%)	S_{N2H4}[c] (%)
1	**[2-NH$_3$]PF$_6$**	0.8	0.2	66.1 **52.9**	330.5	63.1 **50.4**	315.5	1.0 **0.8**	5.0	96.6	98.5
2	**[2-NH$_3$]PF$_6$**	0.8	0.5	127.0 **101.6**	254.0	123.0 **98.4**	246.0	1.3 **1.1**	2.6	94.1	99.0
3	**[2-NH$_3$]PF$_6$**	0.8	1	209.3 **167.5**	209.3	202.5 **162.0**	202.5	2.5 **2.1**	2.5	91.0	98.8
4	**[2-NH$_3$]PF$_6$**	0.8	2	403.5 **322.8**	201.8	393.9 **315.2**	197.0	2.8 **1.8**	1.4	90.8	99.3
5	**[2-NH$_3$]PF$_6$**	0.8	4	507.8 **406.2**	127.0	492.0 **393.6**	123.0	5.2 **4.4**	1.3	86.9	98.9
6	**[2-NH$_3$]PF$_6$**	0.8	8	608.7 **487.0**	76.1	584.4 **467.6**	73.1	10.0 **8.2**	1.2	83.6	98.3
7	**[2-NH$_3$]PF$_6$**	0.8	12	677.6 **542.1**	56.5	652.5 **522.0**	54.4	11.5 **9.2**	1.0	81.7	98.2

[a] Conditions: a Ag/AgCl electrode in saturated KCl solution as reference electrode, a platinum plate as counter electrode, a carbon cloth (1 cm^2) as working electrode, $[NH_3]$ = 2.0 M, [cat.] = 0.01 mM.

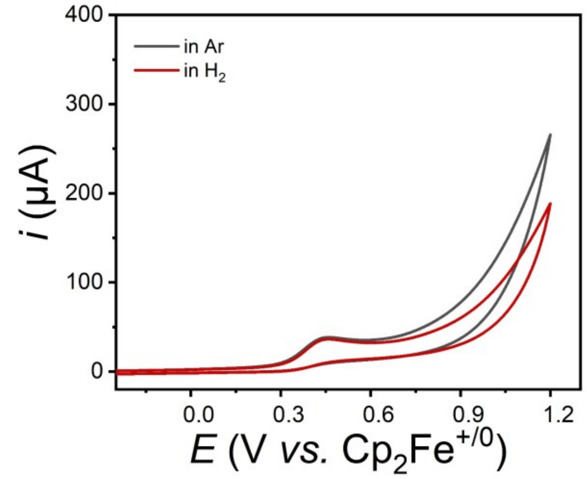

Supplementary Fig. 40 | CV plot of **[2-NH_3]PF_6** in presence of 0.05 M NH_3 in MeCN with scan rate at 100 mV s^{-1} under Ar atmosphere (black line) and H_2 atmosphere (red line).

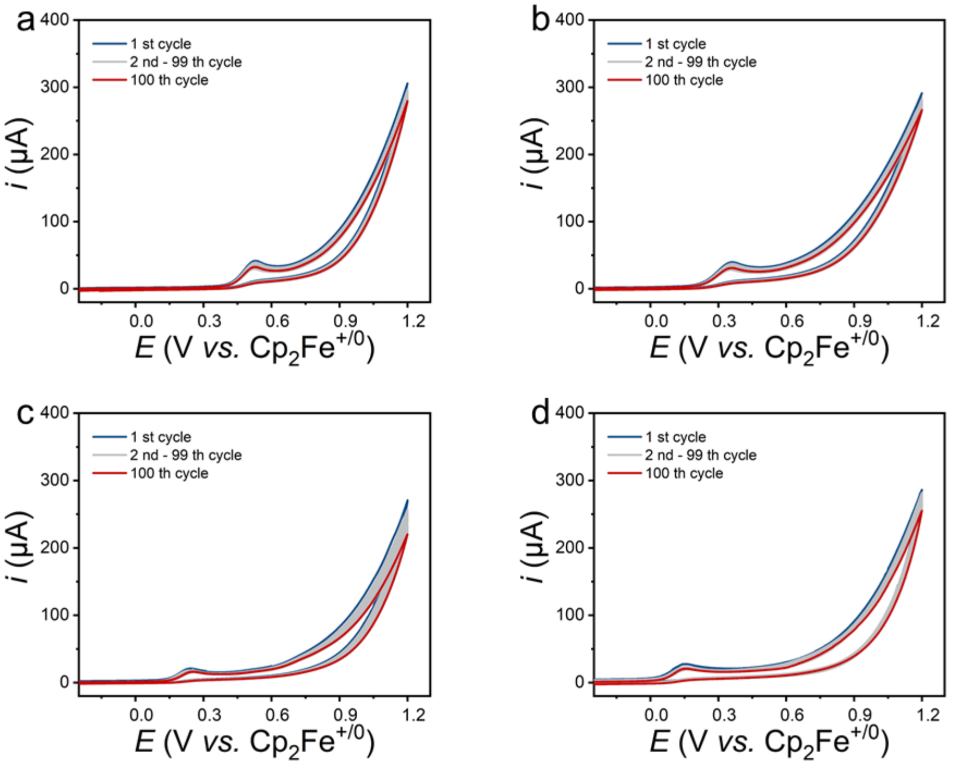

Supplementary Fig. 41 | Multiple repetitive CV scans of complex (a) **[1-NH_3]PF_6**, (b) **[2-NH_3]PF_6**, (c) **[3-NH_3]PF_6**, (d) **[4-NH_3]PF_6** in presence of 0.05 M NH_3 in MeCN with scan rate at 100 mV s^{-1}.

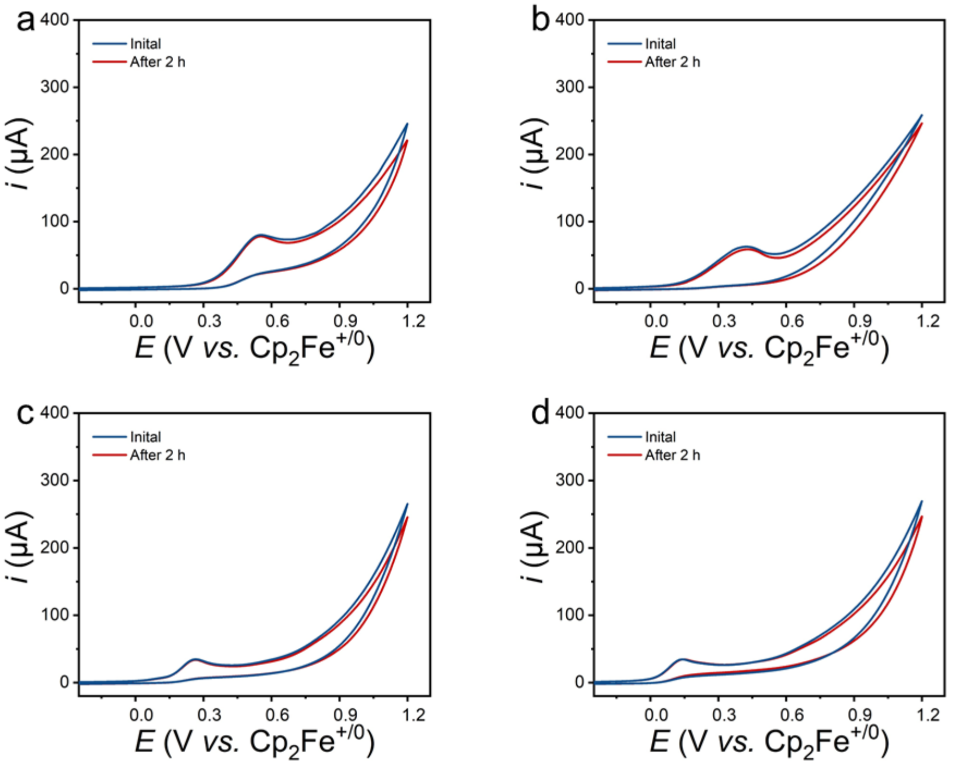

Supplementary Fig. 42 | The CVs before and after electrolysis of complexes (a) **[1-NH_3]PF_6** (1 mM), (b) **[2-NH_3]PF_6** (1 mM), (c) **[3-NH_3]PF_6** (1 mM), (d) **[4-NH_3]PF_6** (1 mM) in presence of 0.05 M NH_3 in MeCN with scan rate at 100 mV s^{-1}.

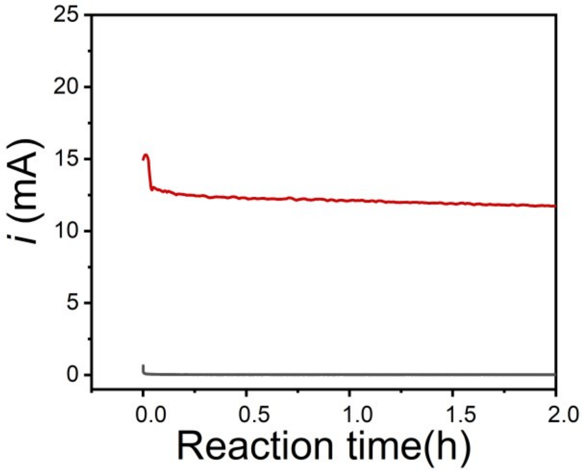

Supplementary Fig. 43 | Comparison of CA response recorded at (red) CPC experiments in the presence of 0.01 mM **[4-NH_3]PF_6** using carbon cloth as working electrode and b) CPC experiments in the absence of **[4-NH_3]PF_6** using carbon cloth which is rinsed with ultradry acetonitrile after CPC experiments with catalyst **[4-NH_3]PF_6**.

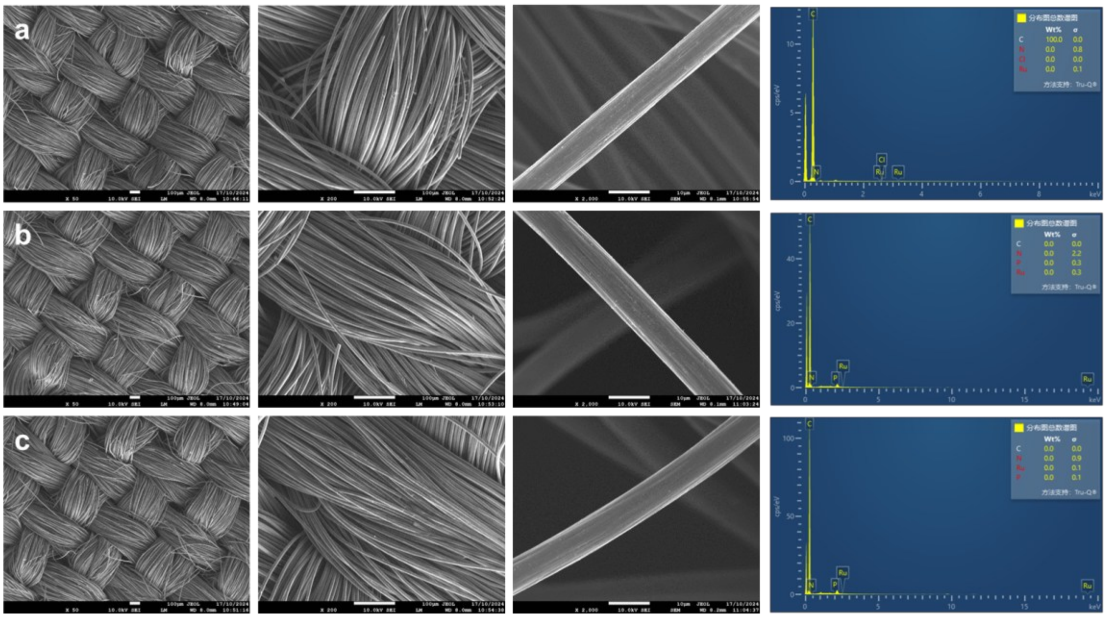

Supplementary Fig. 44 | SEM images of carbon cloth. (a) before; (b) after electrolysis of NH_3 (2.00 M) in presence of **[4-NH_3]PF_6** (0.01 mM) in MeCN solutions under Ar atmosphere at applied potential of 0.8 V *vs.* $Cp_2Fe^{+/0}$ for 2 h; (c) the rinsed carbon cloth after electrolysis.

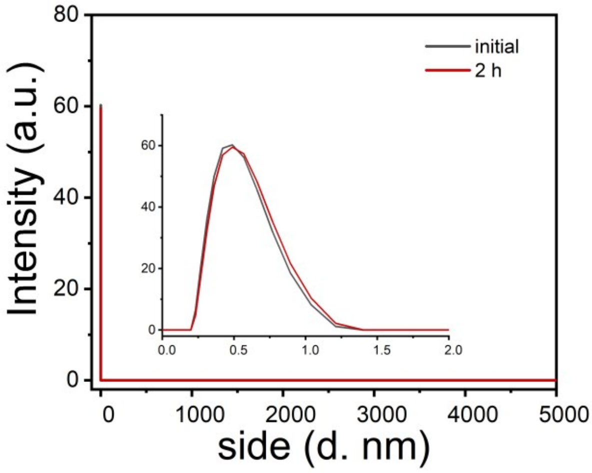

Supplementary Fig. 45 | DLS curves of (black) before and (red) after CPC experiments by **[4-NH_3]PF_6** at E_{app} 0.8 V *vs*. $Cp_2Fe^{+/0}$ for 2 h.

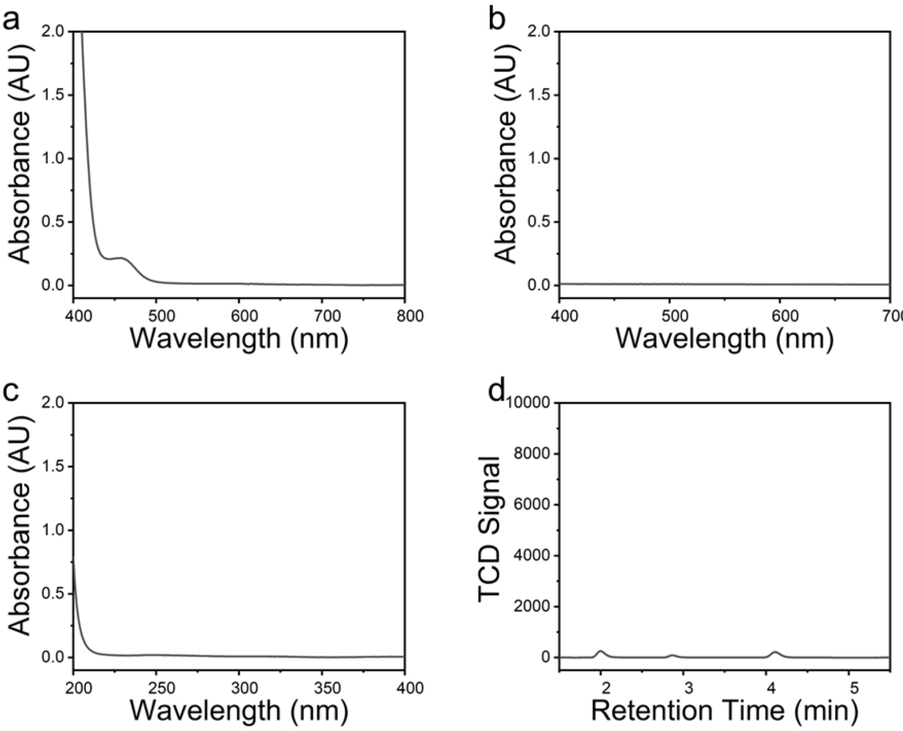

Supplementary Fig. 46 | Determination of (a) N_2H_4, (b) NO_2^-, (c) NO_3^- in the electrolyte and (d) GC-TCD trace of H_2, O_2 and N_2 after the CPC experiment of NH_3 solution (2.00 M) in MeCN containing **[1-NH$_3$]PF$_6$** (0.01 mM) at an applied potential of 0.39 V *vs.* $Cp_2Fe^{+/0}$ for 24 h.

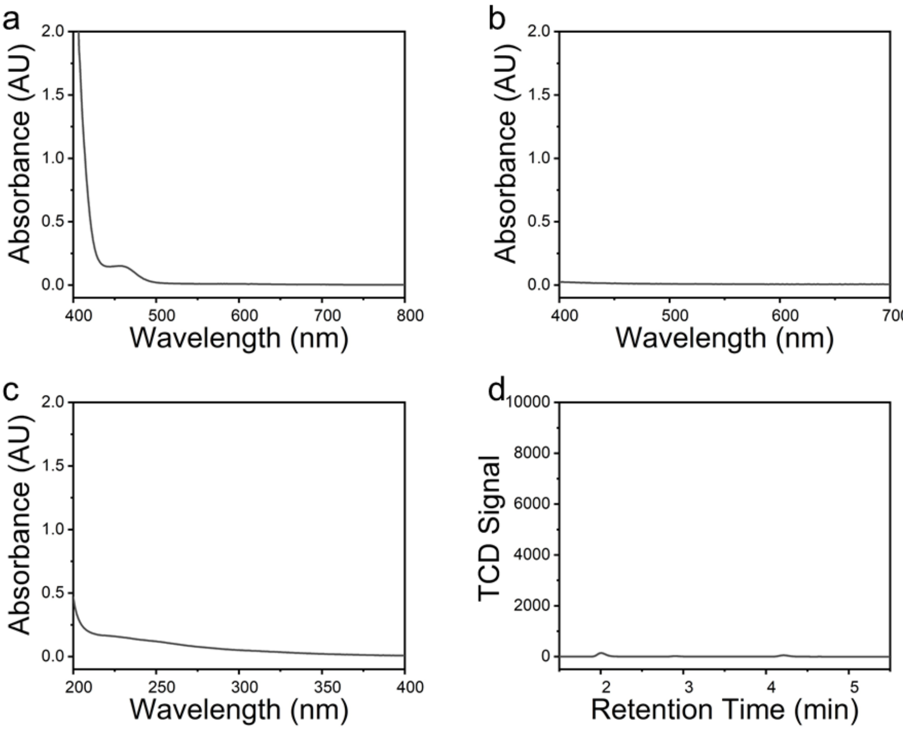

Supplementary Fig. 47 | Determination of (a) N_2H_4, (b) NO_2^-, (c) NO_3^- in the electrolyte and (d) GC-TCD trace of H_2, O_2 and N_2 after the CPC experiment of NH_3 solution (2.00 M) in MeCN containing **[2-NH$_3$]PF$_6$** (0.01 mM) at an applied potential of 0.26 V *vs.* $Cp_2Fe^{+/0}$ for 24 h.

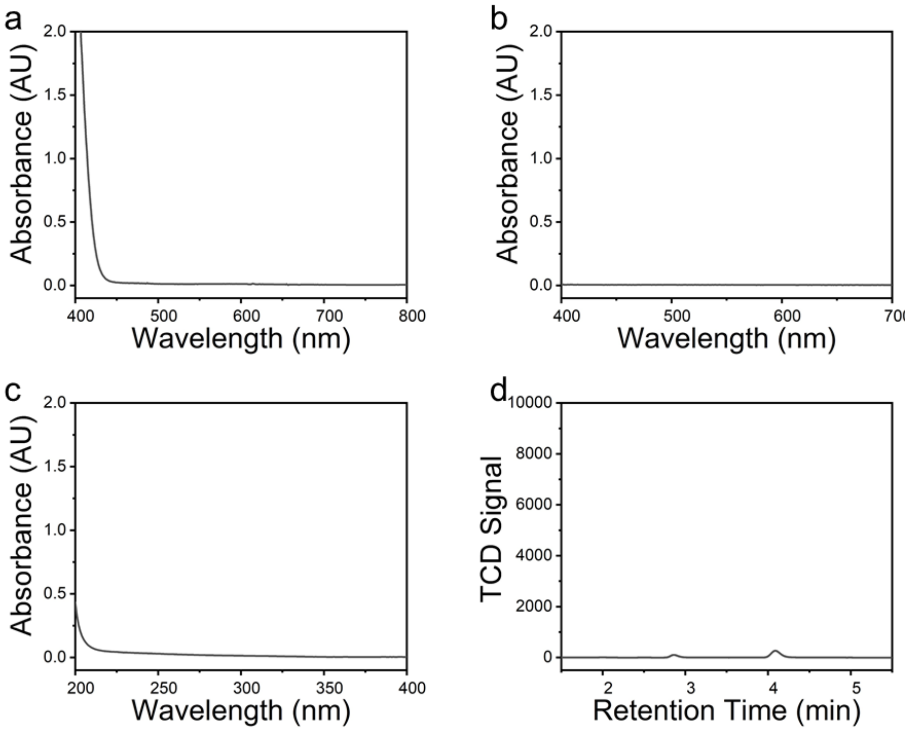

Supplementary Fig. 48 | Determination of (a) N_2H_4, (b) NO_2^-, (c) NO_3^- in the electrolyte and (d) GC-TCD trace of H_2, O_2 and N_2 after the CPC experiment of NH_3 solution (2.00 M) in MeCN containing **[3-NH$_3$]PF$_6$** (0.01 mM) at an applied potential of 0.13 V *vs.* $Cp_2Fe^{+/0}$ for 24 h.

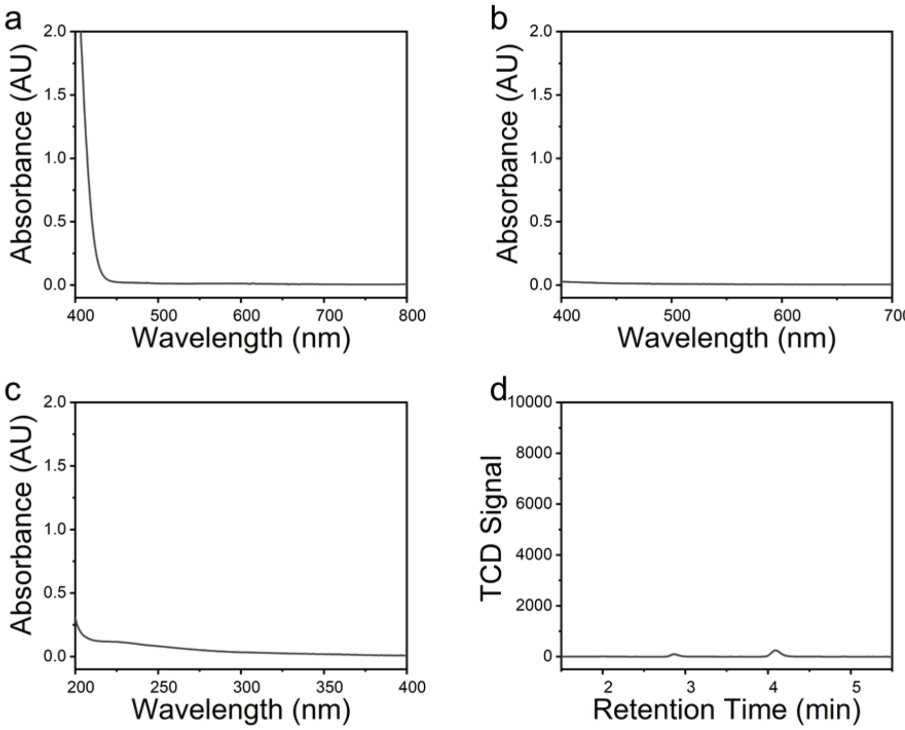

Supplementary Fig. 49 | Determination of (a) N_2H_4, (b) NO_2^-, (c) NO_3^- in the electrolyte and (d) GC-TCD trace of H_2, O_2 and N_2 after the CPC experiment of NH_3 solution (2.00 M) in MeCN containing **[4-NH$_3$]PF$_6$** (0.01 mM) at an applied potential of 0.06 V *vs.* $Cp_2Fe^{+/0}$ for 24 h.

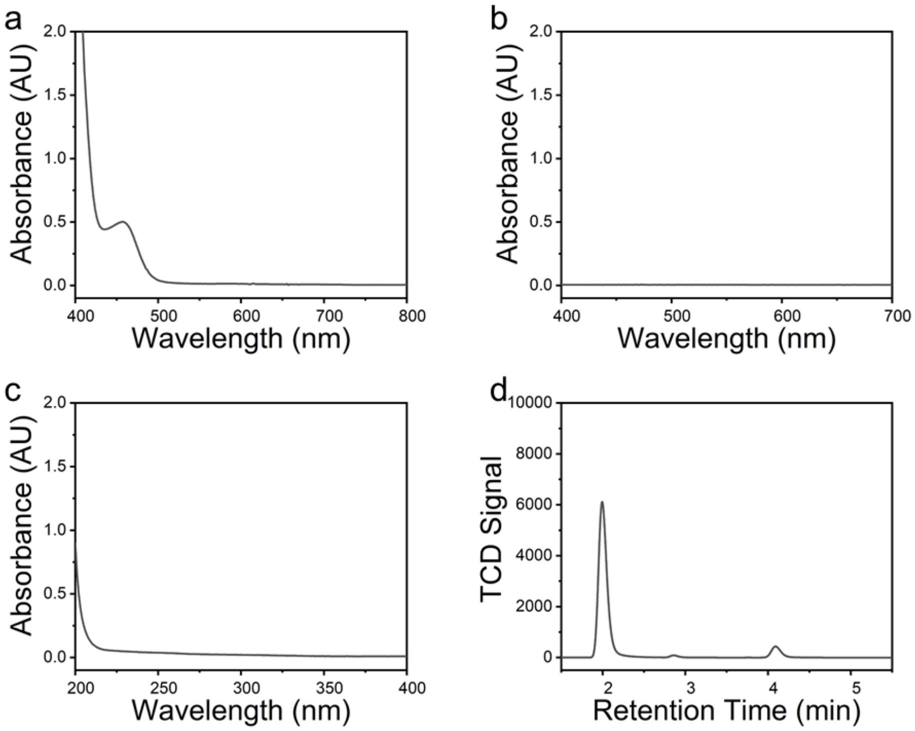

Supplementary Fig. 50 | Determination of (a) N_2H_4, (b) NO_2^-, (c) NO_3^- in the electrolyte and (d) GC-TCD trace of H_2, O_2 and N_2 after the CPC experiment of NH_3 solution (2.00 M) in MeCN containing **[1-NH$_3$]PF$_6$** (0.01 mM) at an applied potential of 0.8 V *vs.* $Cp_2Fe^{+/0}$ for 2 h.

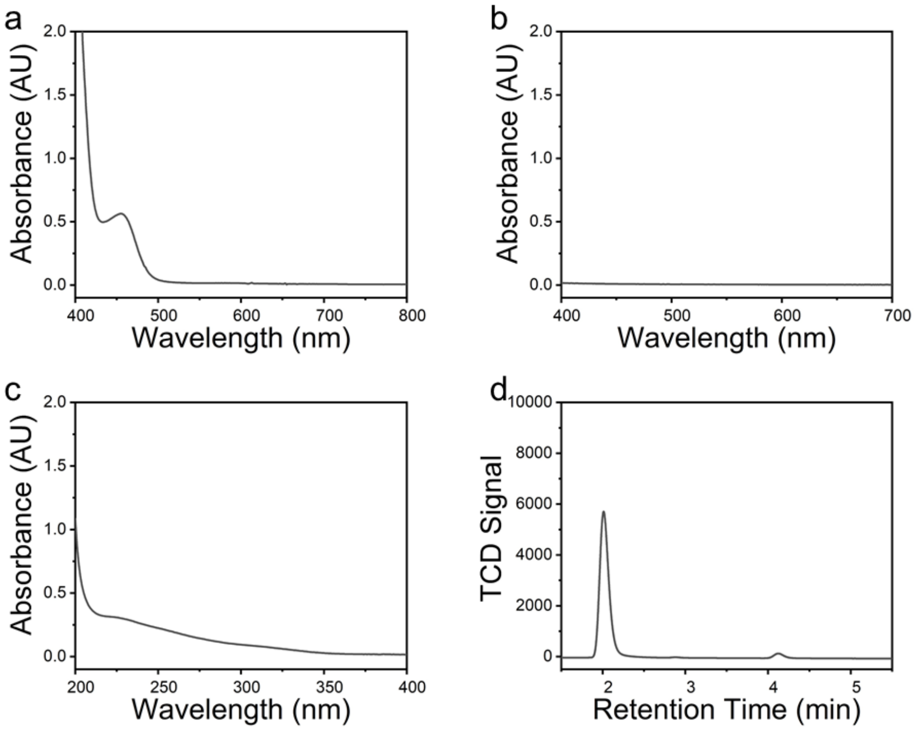

Supplementary Fig. 51 | Determination of (a) N_2H_4, (b) NO_2^-, (c) NO_3^- in the electrolyte and (d) GC-TCD trace of H_2, O_2 and N_2 after the CPC experiment of NH_3 solution (2.00 M) in MeCN containing **[2-NH$_3$]PF$_6$** (0.01 mM) at an applied potential of 0.8 V *vs.* $Cp_2Fe^{+/0}$ for 2 h.

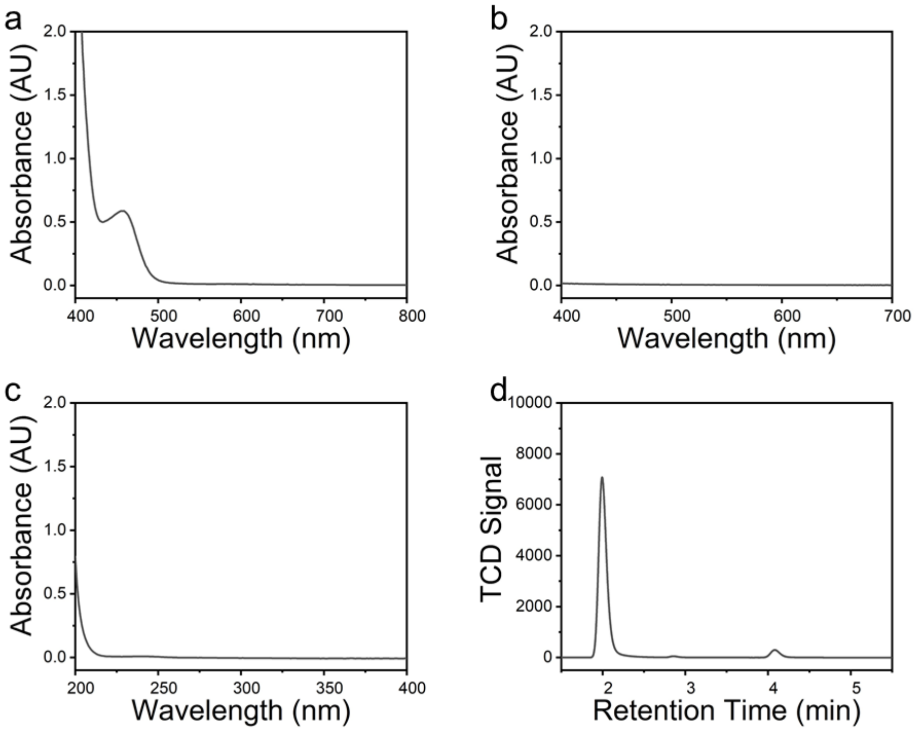

Supplementary Fig. 52 | Determination of (a) N_2H_4, (b) NO_2^-, (c) NO_3^- in the electrolyte and (d) GC-TCD trace of H_2, O_2 and N_2 after the CPC experiment of NH_3 solution (2.00 M) in MeCN containing **[3-NH$_3$]PF$_6$** (0.01 mM) at an applied potential of 0.8 V *vs.* $Cp_2Fe^{+/0}$ for 2 h.

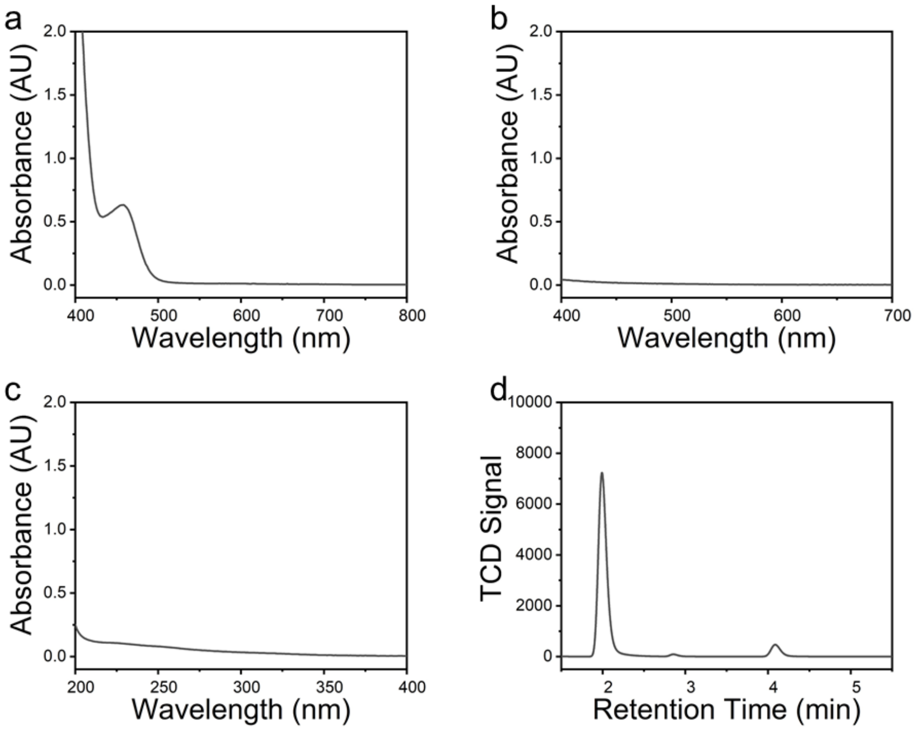

Supplementary Fig. 53 | Determination of (a) N_2H_4, (b) NO_2^-, (c) NO_3^- in the electrolyte and (d) GC-TCD trace of H_2, O_2 and N_2 after the CPC experiment of NH_3 solution (2.00 M) in MeCN containing **[4-NH$_3$]PF$_6$** (0.01 mM) at an applied potential of 0.8 V *vs.* $Cp_2Fe^{+/0}$ for 2 h.

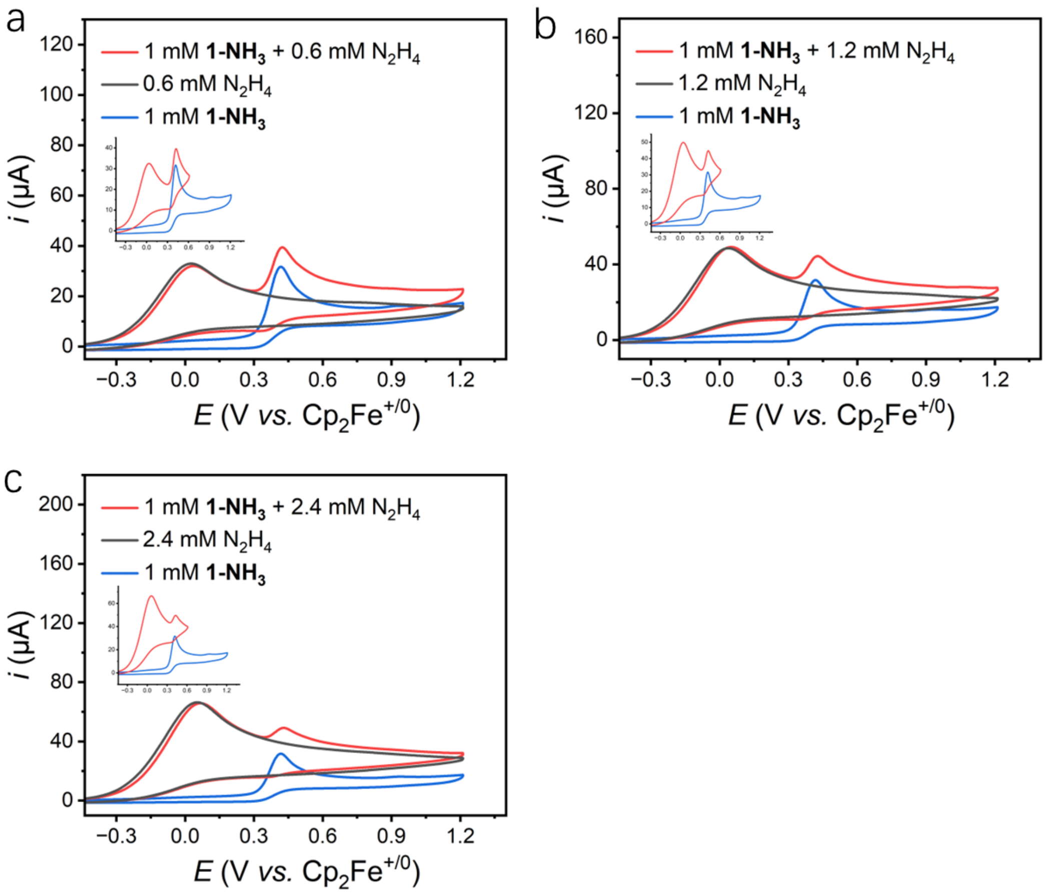

Supplementary Fig. 54 | CVs of 1 mM complex of [**1-NH**$_3$]$^+$ in CH_3CN solution containing 0.6, 1.2 and 2.4 mM N_2H_4 with scan rate at 100 mV s^{-1}. Conditions: 0.1 M Bu_4NPF_6 as supporting electrolytes, glass carbon as working electrode, Ag/AgCl as reference electrode.

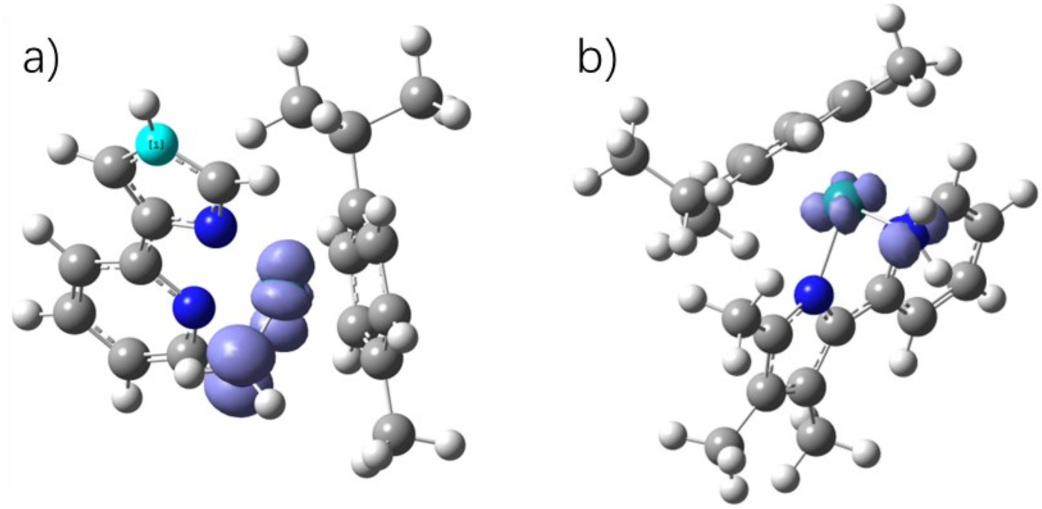

Supplementary Fig. 55 | The spin densities on Ru and N atoms of ^{2}[**1-I**]$^+$ and ^{2}[**4-I**]$^+$.

4. Coordinates from Geometry Optimizations

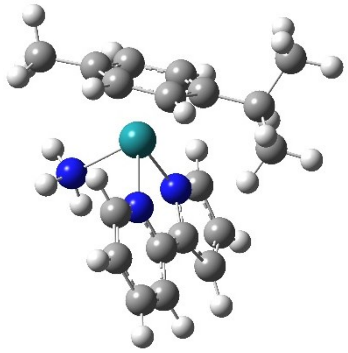

[1][**1-NH$_3$**]$^+$

E = -995.982177 (a.u.)

Charge = 1 Multiplicity = 1

Ru	2.66981	12.2339	16.8348
N	1.26906	11.9545	18.3816
N	3.53892	10.7611	17.986
N	1.62079	10.6282	15.9103
H	2.02546	10.4087	15.1487
H	1.61379	9.92514	16.4559
H	0.78405	10.8796	15.7398
C	1.62455	11.0634	19.3518
C	2.86078	10.3928	19.1186
C	2.01286	14.1717	16.0187
H	1.1702	14.5609	15.9576
C	2.49873	13.3954	14.9553
C	3.80624	12.8448	15.0658
H	4.14938	12.3299	14.3715
C	2.79418	14.3678	17.186
H	2.43729	14.8541	17.8939
C	4.09607	13.8451	17.2998
C	0.7832	10.8818	20.4519
H	1.0287	10.2983	21.1337
C	3.57081	9.41204	19.7964
H	3.32318	8.99339	20.5891
C	4.57724	13.0703	16.2063
H	5.43097	12.7038	16.2536
C	0.08911	12.5877	18.4723
H	-0.15756	13.1684	17.7892
C	4.67479	10.0317	17.9401
H	5.32228	10.0841	17.2749
C	4.72075	9.1801	19.0586
H	5.39644	8.57417	19.2641
C	1.63674	13.0978	13.7623

H	0.74165	12.9072	14.0536
H	1.6271	13.8577	13.1761
H	1.98897	12.337	13.2942
C	-0.76819	12.4163	19.5245
H	-1.57893	12.8694	19.5579
C	-0.40239	11.5605	20.5256
H	-0.96064	11.4397	21.2594
C	4.95712	14.0556	18.5248
H	5.6298	13.3424	18.5272
C	5.69998	15.3592	18.3879
H	5.0703	16.0824	18.3349
H	6.26854	15.4859	19.151
H	6.23412	15.3416	17.5903
C	4.23395	13.9721	19.8188
H	3.72271	13.1604	19.8484
H	4.86648	13.9791	20.5405
H	3.64336	14.7251	19.904

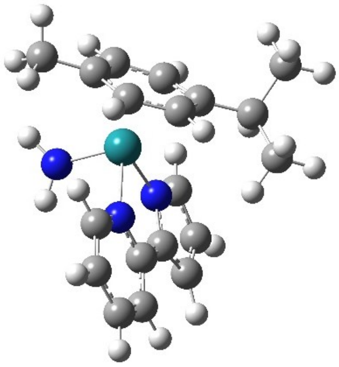

^{2}[**1-I**]$^+$

E = -995.318029 (a.u.)

Charge = 1 Multiplicity = 2

Ru	-0.37311	-0.55155	-0.47675
N	1.52166	-0.79644	0.33089
N	0.57448	1.12915	-1.09079
N	0.28255	-1.44965	-2.03315
H	-0.29132	-2.05241	-2.62263
H	1.23938	-1.36089	-2.37761
C	2.3895	0.23444	0.14128
C	1.86005	1.30598	-0.64867
C	-1.44779	-1.68671	1.10997
H	-1.02437	-2.47411	1.73659
C	-2.06534	-2.03719	-0.11019
C	-2.50931	-0.96581	-0.94303
H	-2.90566	-1.18276	-1.93753

C	-1.39119	-0.33166	1.56811
H	-0.90895	-0.12415	2.52412
C	-1.86341	0.71421	0.77177
C	3.68224	0.17887	0.686
H	4.36031	1.01916	0.52972
C	2.35377	2.5235	-1.13376
H	3.34513	2.93796	-0.95391
C	-2.37025	0.37259	-0.53074
H	-2.66568	1.17372	-1.2116
C	1.90875	-1.8873	1.00848
H	1.17588	-2.69045	1.09768
C	0.23473	2.19103	-1.84759
H	-0.74619	2.26504	-2.31638
C	1.31441	3.08524	-1.89882
H	1.33423	4.03267	-2.43719
C	-2.18329	-3.45572	-0.5525
H	-1.45404	-4.0953	-0.03791
H	-3.19397	-3.82597	-0.31488
H	-2.04484	-3.54614	-1.63902
C	3.1714	-1.99996	1.56414
H	3.44203	-2.90426	2.11068
C	4.07025	-0.93901	1.403
H	5.07323	-0.99421	1.83273
C	-1.79993	2.1651	1.1713
H	-1.55374	2.72031	0.25074
C	-3.18971	2.61143	1.63099
H	-3.49091	2.07509	2.54501
H	-3.18587	3.68965	1.85204
H	-3.95217	2.42577	0.85894
C	-0.73312	2.47223	2.21029
H	0.2573	2.11428	1.88932
H	-0.66692	3.55956	2.36375
H	-0.96738	2.01668	3.18546

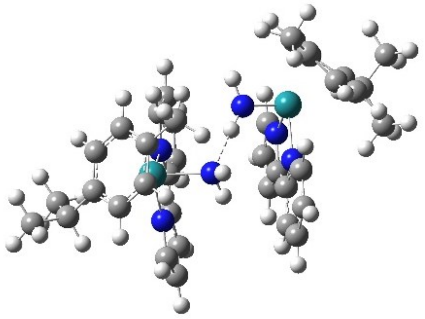

[1][**1-III**]$^{2+}$

E = -1990.661375 (a.u.)

Charge = 2 Multiplicity = 1

Ru	-0.3592	-0.54983	-0.47819
N	1.53361	-0.77934	0.34525
N	0.58468	1.1425	-1.08525
N	0.32618	-1.45611	-2.02956
H	-0.23015	-2.08249	-2.61036
H	1.28987	-1.372	-2.35377
C	2.39717	0.25484	0.15673
C	1.86664	1.324	-0.63588
C	-1.45088	-1.69853	1.10315
H	-1.0261	-2.4794	1.73686
C	-2.04776	-2.05844	-0.12499
C	-2.49692	-0.99367	-0.96485
H	-2.87915	-1.21776	-1.96322
C	-1.41693	-0.34165	1.55906
H	-0.95139	-0.12668	2.52169
C	-1.89584	0.69713	0.7556
C	3.68878	0.20441	0.70485
H	4.36411	1.04705	0.54934
C	2.35707	2.54513	-1.11563
H	3.34577	2.96349	-0.93022
C	-2.38514	0.34713	-0.55159
H	-2.68858	1.14299	-1.23483
C	1.92346	-1.86928	1.02228
H	1.19391	-2.67594	1.10742
C	0.24321	2.20427	-1.84092
H	-0.73632	2.27535	-2.31315
C	1.31896	3.10393	-1.88473
H	1.33676	4.05284	-2.42065
C	-2.14135	-3.47811	-0.56897
H	-1.42001	-4.11099	-0.03537
H	-3.15485	-3.85695	-0.35861
H	-1.97318	-3.56632	-1.65168

C	3.18504	-1.97669	1.58173
H	3.45818	-2.88019	2.12837
C	4.07976	-0.91181	1.42292
H	5.08205	-0.96295	1.85469
C	-1.8645	2.14837	1.15688
H	-1.6347	2.71089	0.23644
C	-3.2636	2.55994	1.62137
H	-3.54843	2.01514	2.53564
H	-3.28537	3.63763	1.84394
H	-4.02342	2.35654	0.85123
C	-0.8028	2.47933	2.19386
H	0.19611	2.15028	1.86823
H	-0.76602	3.56732	2.35223
H	-1.02161	2.01344	3.16767
Ru	3.3228	-2.48163	-4.33697
N	1.5065	-1.78689	-5.06626
N	3.89362	-0.92008	-5.50278
N	3.07544	-1.26171	-2.87121
H	3.38384	-1.45249	-1.91849
H	2.61002	-0.35817	-2.95999
C	1.5724	-0.75484	-5.9502
C	2.9007	-0.27653	-6.19435
C	2.69852	-4.62203	-4.13483
H	1.67702	-4.9817	-3.99716
C	3.48544	-4.33442	-2.99809
C	4.77739	-3.77021	-3.2291
H	5.38406	-3.44469	-2.38113
C	3.21273	-4.48888	-5.46449
H	2.56036	-4.73617	-6.30286
C	4.49375	-3.977	-5.69016
C	0.39822	-0.23965	-6.52214
H	0.47229	0.58299	-7.23494
C	3.46344	0.74616	-6.96848
H	2.9272	1.43257	-7.62286
C	5.24906	-3.56017	-4.53846
H	6.21848	-3.07967	-4.68559
C	0.31771	-2.29248	-4.70646
H	0.33384	-3.09536	-3.968
C	5.07033	-0.33787	-5.80511
H	6.00545	-0.68146	-5.36349
C	4.84786	0.70473	-6.71726
H	5.61175	1.35613	-7.14167
C	2.97197	-4.53552	-1.61344
H	1.87498	-4.58005	-1.59768

H	3.36186	-5.4875	-1.21764
H	3.31423	-3.73376	-0.94384
C	-0.87263	-1.82266	-5.23417
H	-1.8168	-2.26668	-4.91617
C	-0.82511	-0.7794	-6.1664
H	-1.74593	-0.38745	-6.60471
C	5.08756	-3.78122	-7.06025
H	5.67159	-2.84724	-7.00518
C	6.06157	-4.92777	-7.34193
H	5.52814	-5.89032	-7.39486
H	6.56886	-4.76385	-8.30484
H	6.83291	-5.00666	-6.56051
C	4.05397	-3.63113	-8.16534
H	3.33627	-2.8271	-7.94045
H	4.5567	-3.38145	-9.11143
H	3.49086	-4.56346	-8.32935

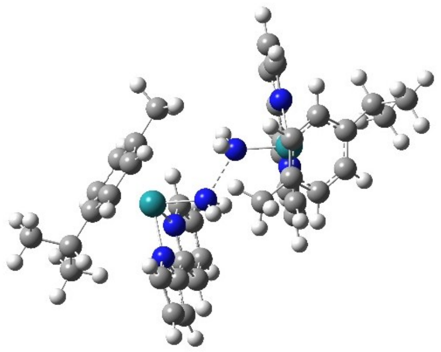

^{1}[**1-IV**]$^{2+}$

E =-1990.642689 (a.u.)

Charge = 2 Multiplicity = 1

Ru	2.80592	-0.66009	-0.29541
N	2.20314	1.33187	-0.24802
N	3.21542	-0.12334	1.62807
N	1.05137	-1.09198	0.44361
H	0.79392	-2.08115	0.45718
H	0.82946	-0.63843	1.33264
C	2.30155	1.9578	0.95722
C	2.84801	1.14277	2.00057
C	3.05336	-0.92757	-2.4949
H	2.48779	-0.43554	-3.28828
C	2.5824	-2.15119	-1.9652
C	3.31738	-2.7408	-0.88527
H	2.95057	-3.65631	-0.41711
C	4.2297	-0.3034	-1.97805
H	4.51315	0.67289	-2.37465

C	4.94972	-0.86635	-0.90795
C	1.89063	3.29358	1.09654
H	1.97801	3.77513	2.07161
C	3.11584	1.32276	3.36543
H	2.94031	2.2278	3.94588
C	4.4551	-2.10128	-0.36251
H	4.95058	-2.52601	0.51314
C	1.71208	1.99325	-1.30963
H	1.65456	1.43595	-2.24615
C	3.70129	-0.75994	2.71152
H	4.04991	-1.79111	2.65681
C	3.66015	0.10644	3.81588
H	3.99603	-0.1286	4.82575
C	1.34591	-2.80461	-2.48597
H	0.71742	-2.09325	-3.03911
H	1.63045	-3.61749	-3.17389
H	0.75959	-3.25649	-1.67258
C	1.29923	3.31046	-1.23301
H	0.90854	3.80604	-2.12191
C	1.3921	3.97028	-0.00098
H	1.07241	5.0099	0.09395
C	6.16839	-0.21586	-0.30121
H	6.17748	-0.5211	0.75871
C	7.42441	-0.78022	-0.96609
H	7.4557	-0.5101	-2.03401
H	8.32723	-0.37405	-0.48467
H	7.46311	-1.87799	-0.89091
C	6.13276	1.30481	-0.35315
H	5.20461	1.69943	0.08823
H	6.98156	1.7169	0.21262
H	6.21371	1.68321	-1.38444
Ru	-2.62233	-0.45554	0.40336
N	-2.55691	1.60763	0.04692
N	-3.16251	-0.33654	-1.56871
N	-0.82671	-0.42781	-0.44108
H	-0.72427	-1.00339	-1.28038
H	-0.4559	0.50622	-0.62939
C	-2.91347	2.00092	-1.20669
C	-3.22743	0.92388	-2.10186
C	-3.01745	-0.31733	2.58811
H	-2.77286	0.513	3.25368
C	-2.08398	-1.356	2.41324
C	-2.40621	-2.38502	1.46013
H	-1.67131	-3.16281	1.2416

C	-4.25841	-0.29397	1.87516
H	-4.9211	0.56266	2.00099
C	-4.58807	-1.3194	0.96746
C	-2.9509	3.3656	-1.53554
H	-3.24291	3.65998	-2.54467
C	-3.58197	0.83826	-3.45574
H	-3.71632	1.6723	-4.14381
C	-3.62788	-2.3735	0.76713
H	-3.82637	-3.14167	0.01708
C	-2.21055	2.5233	0.96377
H	-1.90044	2.1437	1.93839
C	-3.45478	-1.22316	-2.53901
H	-3.46367	-2.29588	-2.34551
C	-3.72451	-0.53414	-3.7327
H	-3.99839	-0.98731	-4.68545
C	-0.79977	-1.38544	3.17811
H	-0.3863	-0.37559	3.3115
H	-0.98689	-1.80347	4.18081
H	-0.05351	-2.0267	2.69289
C	-2.2415	3.88215	0.70136
H	-1.95902	4.58946	1.48184
C	-2.62605	4.30713	-0.57507
H	-2.65835	5.37201	-0.81714
C	-5.87607	-1.3314	0.18206
H	-5.64096	-1.81135	-0.78248
C	-6.89152	-2.21036	0.91351
H	-7.15176	-1.77596	1.89221
H	-7.81608	-2.29721	0.32226
H	-6.5008	-3.22548	1.08407
C	-6.43235	0.05521	-0.10561
H	-5.68161	0.6961	-0.59289
H	-7.29951	-0.02405	-0.77811
H	-6.77504	0.55851	0.81228

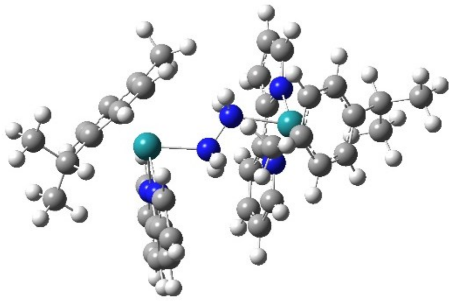

[1][**1-V**]$^{2+}$
E = -1990.726675 (a.u.)
Charge = 2 Multiplicity = 1

Ru	-2.58323	-0.65488	0.31656
N	-2.07444	1.37523	0.30021
N	-2.94953	-0.12817	-1.61822
N	-0.6238	-0.89903	-0.48275
H	-0.45053	-1.88353	-0.70586
H	-0.59677	-0.40043	-1.37768
C	-2.1804	2.00334	-0.90385
C	-2.64412	1.16224	-1.96924
C	-2.82733	-0.9752	2.49748
H	-2.32287	-0.45412	3.31347
C	-2.24596	-2.13593	1.95268
C	-2.89989	-2.75971	0.83546
H	-2.43841	-3.62463	0.35449
C	-4.01413	-0.4174	1.93045
H	-4.37156	0.54039	2.31238
C	-4.67333	-1.02579	0.83993
C	-1.84839	3.36187	-1.02801
H	-1.93575	3.84201	-2.00379
C	-2.86619	1.33809	-3.34231
H	-2.71948	2.25643	-3.90974
C	-4.08485	-2.22135	0.30699
H	-4.52007	-2.67209	-0.58786
C	-1.66032	2.06343	1.37834
H	-1.59429	1.50542	2.31412
C	-3.35111	-0.78114	-2.728
H	-3.6409	-1.83131	-2.69544
C	-3.31698	0.09508	-3.82349
H	-3.59497	-0.15042	-4.84831
C	-0.96932	-2.69455	2.49016
H	-0.4067	-1.9407	3.05835
H	-1.19531	-3.53203	3.17007
H	-0.33675	-3.09443	1.68378
C	-1.32431	3.40423	1.31745

H	-0.99104	3.91961	2.21886
C	-1.41907	4.06203	0.08502
H	-1.15645	5.11898	0.0011
C	-5.91453	-0.45457	0.19873
H	-5.85995	-0.73361	-0.86687
C	-7.14871	-1.12665	0.80032
H	-7.24147	-0.88643	1.87192
H	-8.06133	-0.77801	0.29274
H	-7.10155	-2.22214	0.70039
C	-5.99966	1.0627	0.28222
H	-5.08748	1.53865	-0.10946
H	-6.85289	1.42198	-0.31239
H	-6.15332	1.40976	1.31624
Ru	2.4594	-0.41517	-0.47941
N	2.33851	1.62501	-0.003
N	2.82009	-0.40733	1.52732
N	0.47663	-0.43792	0.29151
H	0.49659	-0.98762	1.1559
H	0.24609	0.51564	0.5864
C	2.57236	1.94522	1.29965
C	2.82186	0.81547	2.14832
C	3.0028	-0.1416	-2.60991
H	2.81381	0.72822	-3.24235
C	2.05506	-1.17797	-2.56976
C	2.29601	-2.25934	-1.65315
H	1.53839	-3.03829	-1.53543
C	4.17499	-0.16989	-1.79325
H	4.84528	0.69027	-1.80305
C	4.43179	-1.25117	-0.92526
C	2.539	3.28499	1.71835
H	2.73125	3.51827	2.76663
C	3.04308	0.64153	3.52097
H	3.09978	1.42817	4.27258
C	3.46054	-2.30792	-0.8701
H	3.59289	-3.12003	-0.15206
C	2.04927	2.59247	-0.88755
H	1.84142	2.27166	-1.9092
C	3.02425	-1.35557	2.46489
H	3.05992	-2.41329	2.20414
C	3.1701	-0.74588	3.72045
H	3.3524	-1.25937	4.66435
C	0.85169	-1.16035	-3.45711
H	0.45412	-0.14302	-3.58141
H	1.1385	-1.52866	-4.45559

H	0.05641	-1.82138	-3.08731
C	2.00744	3.93016	-0.53478
H	1.77121	4.68059	-1.28998
C	2.26316	4.27906	0.79621
H	2.23647	5.32561	1.10844
C	5.65019	-1.31517	-0.03814
H	5.33648	-1.84852	0.87391
C	6.72283	-2.15632	-0.73131
H	7.0636	-1.66967	-1.65938
H	7.59511	-2.28035	-0.07125
H	6.3477	-3.15851	-0.99028
C	6.18062	0.05002	0.37353
H	5.39012	0.66723	0.82686
H	6.98497	-0.07075	1.11454
H	6.6033	0.60149	-0.48106

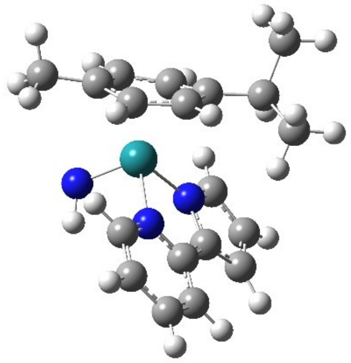

^{1}[**1-II**]$^{+}$

E = -994.661214 (a.u.)

Charge = 1 Multiplicity = 1

Ru	-0.28415	-0.65106	-0.55754
N	1.59297	-0.77671	0.23567
N	0.4988	1.0878	-1.13719
N	0.20433	-1.83245	-1.79552
H	1.23594	-1.95121	-1.84138
C	2.37029	0.33352	0.10388
C	1.76024	1.3754	-0.66591
C	-1.34131	-1.6404	1.13508
H	-0.87196	-2.4002	1.76364
C	-2.08827	-2.04983	-0.00341
C	-2.56316	-1.02505	-0.85393
H	-3.04301	-1.27449	-1.80229
C	-1.31536	-0.27735	1.57586
H	-0.81572	-0.04568	2.51735
C	-1.84262	0.7261	0.77432

C	3.64714	0.37125	0.68078
H	4.25023	1.27402	0.57341
C	2.14754	2.63003	-1.13436
H	3.09582	3.12855	-0.93791
C	-2.33564	0.32499	-0.51679
H	-2.64806	1.10059	-1.21973
C	2.05583	-1.8582	0.88274
H	1.38835	-2.72109	0.92523
C	0.07929	2.12511	-1.90016
H	-0.89087	2.11334	-2.39441
C	1.07456	3.10353	-1.92061
H	1.02472	4.051	-2.45639
C	-2.27202	-3.48787	-0.33645
H	-1.48275	-4.10525	0.11246
H	-3.23975	-3.82073	0.07392
H	-2.29407	-3.64656	-1.4225
C	3.31187	-1.87884	1.46338
H	3.65128	-2.77655	1.98143
C	4.11553	-0.73738	1.36486
H	5.10964	-0.72165	1.8175
C	-1.82838	2.18713	1.13174
H	-1.60557	2.72577	0.19516
C	-3.23297	2.59657	1.58369
H	-3.51289	2.07343	2.51189
H	-3.2651	3.67959	1.77684
H	-3.9897	2.36422	0.8188
C	-0.77061	2.55739	2.15926
H	0.22916	2.21839	1.84687
H	-0.7363	3.6502	2.28001
H	-0.99125	2.12379	3.14749

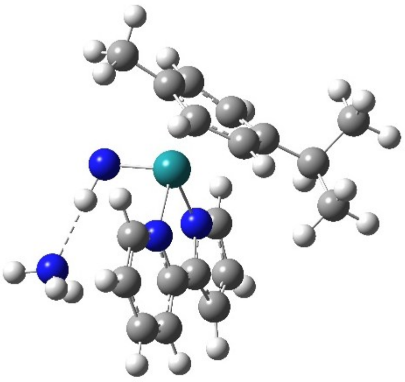

[1]**[1-VI]**$^+$

E = -1051.123066 (a.u.)

Charge = 1 Multiplicity = 1

Ru	-0.26681	-0.63259	-0.54805
N	1.60589	-0.75366	0.2776
N	0.52868	1.11536	-1.1079
N	0.2769	-1.77736	-1.81809
H	1.31183	-1.8571	-1.86138
C	2.39776	0.34266	0.12511
C	1.79722	1.38607	-0.64959
C	-1.38764	-1.66729	1.10836
H	-0.93097	-2.42633	1.74676
C	-2.06878	-2.07735	-0.07003
C	-2.52778	-1.05128	-0.92901
H	-2.96117	-1.29958	-1.89977
C	-1.40527	-0.31051	1.56238
H	-0.95365	-0.07869	2.52788
C	-1.91331	0.69373	0.74695
C	3.67968	0.36957	0.69224
H	4.29644	1.26031	0.5641
C	2.1955	2.63656	-1.12145
H	3.15194	3.12296	-0.93442
C	-2.35491	0.29918	-0.5638
H	-2.65936	1.07539	-1.26953
C	2.05384	-1.82868	0.94536
H	1.37368	-2.67995	1.00856
C	0.11312	2.15769	-1.86489
H	-0.86433	2.16063	-2.34442
C	1.12024	3.12433	-1.89538
H	1.07496	4.07396	-2.42779
C	-2.20812	-3.51407	-0.42917
H	-1.44968	-4.12811	0.07394
H	-3.20169	-3.86135	-0.1006
H	-2.14321	-3.66101	-1.51543

C	3.31199	-1.85849	1.52099
H	3.63863	-2.75098	2.05603
C	4.1349	-0.73357	1.39344
H	5.13276	-0.72647	1.83791
C	-1.93597	2.15059	1.12291
H	-1.74111	2.70763	0.19118
C	-3.34486	2.51481	1.59963
H	-3.59548	1.97443	2.52637
H	-3.40466	3.59444	1.80494
H	-4.10571	2.26881	0.8432
C	-0.87454	2.53741	2.14117
H	0.13133	2.23897	1.80736
H	-0.8758	3.62838	2.28216
H	-1.06397	2.07905	3.12457
N	3.32272	-2.01202	-1.94549
H	3.63789	-2.96079	-1.92273
H	3.61906	-1.5423	-1.1139
H	3.70951	-1.55137	-2.74437

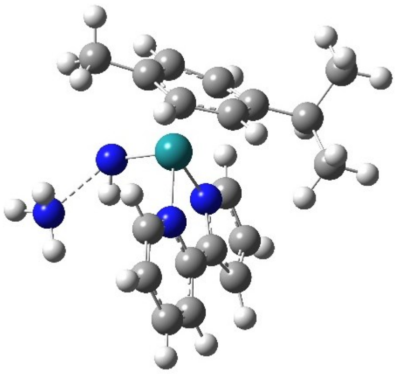

^{1}[**1-VII**]$^{+}$

E = -1051.114842 (a.u.)

Charge = 1 Multiplicity = 1

Ru	0.20568	-0.56952	0.40091
N	-1.49982	-0.06416	-0.62695
N	-0.21426	1.24634	1.16537
N	-0.67127	-1.33231	1.80131
H	-1.38333	-0.70127	2.20725
C	-2.03454	1.15559	-0.33939
C	-1.30664	1.89249	0.64626
C	1.10968	-1.85975	-1.25056
H	0.54208	-2.4058	-2.00705
C	1.42871	-2.51229	-0.03507
C	2.09606	-1.73311	0.94464
H	2.2869	-2.15373	1.93448

C	1.60793	-0.5589	-1.5726
H	1.3855	-0.14373	-2.5569
C	2.25671	0.20821	-0.61089
C	-3.21285	1.57617	-0.97637
H	-3.62304	2.55754	-0.73369
C	-1.43655	3.14597	1.25863
H	-2.20647	3.88902	1.05432
C	2.41145	-0.38092	0.69518
H	2.85815	0.217	1.4924
C	-2.11284	-0.88239	-1.49739
H	-1.64894	-1.85588	-1.65996
C	0.34962	2.04066	2.09636
H	1.23233	1.72658	2.65283
C	-0.37884	3.23735	2.18146
H	-0.15837	4.0723	2.84635
C	1.02824	-3.92127	0.23474
H	0.25007	-4.25358	-0.46513
H	1.90223	-4.58232	0.11825
H	0.66243	-4.0328	1.26554
C	-3.27817	-0.52227	-2.14896
H	-3.74219	-1.2184	-2.84863
C	-3.83319	0.73607	-1.88305
H	-4.75305	1.05049	-2.38169
C	2.74573	1.61321	-0.84762
H	2.6436	2.13545	0.11876
C	4.23298	1.57325	-1.2056
H	4.38902	1.05144	-2.16345
H	4.62691	2.59628	-1.30502
H	4.82209	1.05443	-0.43384
C	1.93288	2.37259	-1.88599
H	0.85983	2.36629	-1.63888
H	2.26628	3.42028	-1.92627
H	2.05828	1.95015	-2.8954
N	-2.83959	-2.30416	1.26938
H	-3.63309	-1.78285	0.89652
H	-2.60103	-3.0223	0.58711
H	-3.1497	-2.77956	2.11592

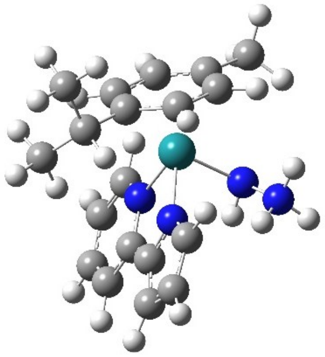

[1][**1-VIII**]$^+$

E = -1051.172142 (a.u.)

Charge = 1 Multiplicity = 1

Ru	-0.26681	-0.63259	-0.54805
N	1.60589	-0.75366	0.2776
N	0.52868	1.11536	-1.1079
N	0.2769	-1.77736	-1.81809
H	1.31183	-1.8571	-1.86138
C	2.39776	0.34266	0.12511
C	1.79722	1.38607	-0.64959
C	-1.38764	-1.66729	1.10836
H	-0.93097	-2.42633	1.74676
C	-2.06878	-2.07735	-0.07003
C	-2.52778	-1.05128	-0.92901
H	-2.96117	-1.29958	-1.89977
C	-1.40527	-0.31051	1.56238
H	-0.95365	-0.07869	2.52788
C	-1.91331	0.69373	0.74695
C	3.67968	0.36957	0.69224
H	4.29644	1.26031	0.5641
C	2.1955	2.63656	-1.12145
H	3.15194	3.12296	-0.93442
C	-2.35491	0.29918	-0.5638
H	-2.65936	1.07539	-1.26953
C	2.05384	-1.82868	0.94536
H	1.37368	-2.67995	1.00856
C	0.11312	2.15769	-1.86489
H	-0.86433	2.16063	-2.34442
C	1.12024	3.12433	-1.89538
H	1.07496	4.07396	-2.42779
C	-2.20812	-3.51407	-0.42917
H	-1.44968	-4.12811	0.07394
H	-3.20169	-3.86135	-0.1006
H	-2.14321	-3.66101	-1.51543

C	3.31199	-1.85849	1.52099
H	3.63863	-2.75098	2.05603
C	4.1349	-0.73357	1.39344
H	5.13276	-0.72647	1.83791
C	-1.93597	2.15059	1.12291
H	-1.74111	2.70763	0.19118
C	-3.34486	2.51481	1.59963
H	-3.59548	1.97443	2.52637
H	-3.40466	3.59444	1.80494
H	-4.10571	2.26881	0.8432
C	-0.87454	2.53741	2.14117
H	0.13133	2.23897	1.80736
H	-0.8758	3.62838	2.28216
H	-1.06397	2.07905	3.12457
N	-0.52935	-2.71782	-3.1261
H	-1.49809	-2.51017	-3.26186
H	-0.42775	-3.67734	-2.86342
H	-0.03021	-2.55032	-3.97628

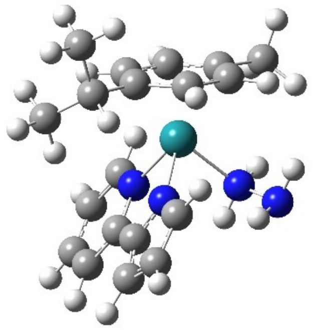

^{1}[**1-IX**]$^{+}$
E = -1051.195408 (a.u.)
Charge = 1 Multiplicity = 1

Ru	0.45849	-0.48189	0.17384
N	-1.40538	-0.64687	-0.76232
N	-0.66638	0.88506	1.19487
N	-0.37014	-1.86837	1.49664
H	-1.30069	-1.90437	1.13225
C	-2.3865	0.16156	-0.27528
C	-1.9735	1.00962	0.80757
C	1.75318	-1.38465	-1.39929
H	1.47236	-2.12594	-2.15025
C	2.22926	-1.81777	-0.14761
C	2.5222	-0.81507	0.84068
H	2.84556	-1.11892	1.83886
C	1.55454	0.00567	-1.67687

H	1.10308	0.28641	-2.62991
C	1.84961	0.99898	-0.72116
C	-3.67845	0.12293	-0.82368
H	-4.44717	0.78231	-0.41747
C	-2.61	1.97564	1.60088
H	-3.64967	2.29308	1.52686
C	2.33728	0.55048	0.55451
H	2.50319	1.28668	1.34465
C	-1.6814	-1.50024	-1.75958
H	-0.85975	-2.13632	-2.09301
C	-0.44911	1.74435	2.21478
H	0.53242	1.83399	2.68164
C	-1.63409	2.44228	2.49987
H	-1.76019	3.20322	3.27016
C	2.38163	-3.26461	0.17856
H	1.83077	-3.89356	-0.53403
H	3.44617	-3.54726	0.14006
H	2.01492	-3.47561	1.19354
C	-2.93499	-1.58269	-2.34292
H	-3.10807	-2.28984	-3.15529
C	-3.95158	-0.74914	-1.86309
H	-4.95262	-0.7881	-2.29916
C	1.64878	2.47454	-0.96444
H	1.39782	2.9118	0.01696
C	2.964	3.0973	-1.43324
H	3.26443	2.68596	-2.4107
H	2.85882	4.18812	-1.54092
H	3.77945	2.90387	-0.71896
C	0.50573	2.78453	-1.92058
H	-0.42935	2.30032	-1.59853
H	0.33116	3.87043	-1.95745
H	0.72845	2.45495	-2.9478
N	-0.15043	-1.49802	2.85744
H	0.84473	-1.60795	3.08421
H	-0.39901	-0.51561	3.07319
H	0.40648	-2.09185	0.90764

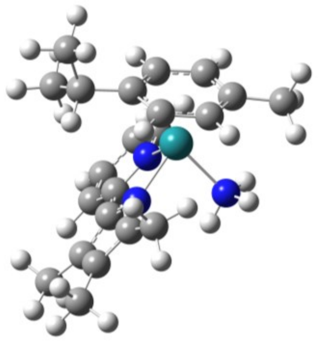

[1][**4-NH$_3$**]$^+$

E = -1113.716352 (a.u.)

Charge = 1 Multiplicity = 1

Ru	-0.53737	-0.45139	-0.36084
N	1.31889	-0.86045	0.50899
N	0.68705	0.88698	-1.29297
N	0.17244	-1.79061	-1.84551
H	-0.53444	-2.03236	-2.5419
H	0.96201	-1.38803	-2.35555
H	0.49886	-2.66948	-1.43802
C	2.35944	-0.09708	0.07612
C	2.00548	0.87488	-0.91773
C	-1.91285	-1.44578	1.0581
H	-1.73239	-2.34978	1.64269
C	-2.45918	-1.55148	-0.23739
C	-2.62548	-0.35106	-1.0071
H	-2.99816	-0.41163	-2.03129
C	-1.48747	-0.18003	1.56224
H	-0.96279	-0.1539	2.51915
C	-1.65095	1.01545	0.82422
C	3.64946	-0.29683	0.59395
H	4.46834	0.32766	0.23336
C	2.70636	1.85461	-1.63534
C	-2.23099	0.89256	-0.47938
H	-2.28615	1.77864	-1.1165
C	1.52864	-1.82063	1.42208
H	0.65705	-2.408	1.71719
C	0.52794	1.84093	-2.23194
C	1.75944	2.46953	-2.4747
C	-2.82453	-2.87755	-0.81837
H	-2.25582	-3.69148	-0.34851
H	-3.89684	-3.06589	-0.64576
H	-2.65878	-2.89881	-1.90482
C	2.77688	-2.06359	1.96974
H	2.89753	-2.85273	2.71305

C	3.85638	-1.27986	1.5454
H	4.85512	-1.4438	1.95709
C	-1.20895	2.36831	1.32745
H	-0.91774	2.94444	0.43292
C	-2.39425	3.07858	1.98105
H	-2.731	2.53214	2.87713
H	-2.11164	4.09693	2.29001
H	-3.24847	3.15729	1.29079
C	-0.00655	2.30777	2.25899
H	0.83171	1.76198	1.79971
H	0.33827	3.32641	2.49201
H	-0.25234	1.81995	3.2156
C	4.20725	2.17863	-1.51732
H	4.43153	2.46883	-0.51214
H	4.78043	1.31257	-1.77484
H	4.45264	2.97962	-2.18298
C	2.00926	3.61584	-3.47227
H	2.37747	4.47208	-2.94675
H	2.73105	3.30556	-4.19866
H	1.09283	3.86635	-3.96452
C	-0.84504	2.11247	-2.87445
H	-1.19407	1.22448	-3.35876
H	-1.54229	2.40298	-2.11659
H	-0.75269	2.89861	-3.59441

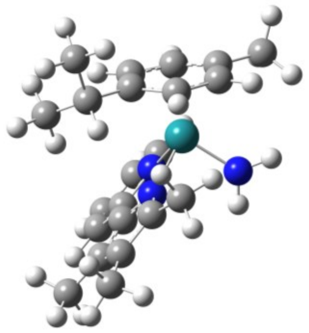

^{2}[**4-I**]$^{+}$

E = -1113.055249 (a.u.)

Charge = 1 Multiplicity = 2

Ru	-0.53737	-0.45139	-0.36084
N	1.31889	-0.86045	0.50899
N	0.68705	0.88698	-1.29297
N	0.17244	-1.79061	-1.84551
H	-0.53444	-2.03236	-2.5419
H	0.96201	-1.38803	-2.35555
C	2.35944	-0.09708	0.07612

C	2.00548	0.87488	-0.91773
C	-1.91285	-1.44578	1.0581
H	-1.73239	-2.34978	1.64269
C	-2.45918	-1.55148	-0.23739
C	-2.62548	-0.35106	-1.0071
H	-2.99816	-0.41163	-2.03129
C	-1.48747	-0.18003	1.56224
H	-0.96279	-0.1539	2.51915
C	-1.65095	1.01545	0.82422
C	3.64946	-0.29683	0.59395
H	4.46834	0.32766	0.23336
C	2.70636	1.85461	-1.63534
C	-2.23099	0.89256	-0.47938
H	-2.28615	1.77864	-1.1165
C	1.52864	-1.82063	1.42208
H	0.65705	-2.408	1.71719
C	0.52794	1.84093	-2.23194
C	1.75944	2.46953	-2.4747
C	-2.82453	-2.87755	-0.81837
H	-2.25582	-3.69148	-0.34851
H	-3.89684	-3.06589	-0.64576
H	-2.65878	-2.89881	-1.90482
C	2.77688	-2.06359	1.96974
H	2.89753	-2.85273	2.71305
C	3.85638	-1.27986	1.5454
H	4.85512	-1.4438	1.95709
C	-1.20895	2.36831	1.32745
H	-0.91774	2.94444	0.43292
C	-2.39425	3.07858	1.98105
H	-2.731	2.53214	2.87713
H	-2.11164	4.09693	2.29001
H	-3.24847	3.15729	1.29079
C	-0.00655	2.30777	2.25899
H	0.83171	1.76198	1.79971
H	0.33827	3.32641	2.49201
H	-0.25234	1.81995	3.2156
C	4.20725	2.17863	-1.51732
H	4.43153	2.46883	-0.51214
H	4.78043	1.31257	-1.77484
H	4.45264	2.97962	-2.18298
C	2.00926	3.61584	-3.47227
H	2.37747	4.47208	-2.94675
H	2.73105	3.30556	-4.19866
H	1.09283	3.86635	-3.96452

C	-0.84504	2.11247	-2.87445
H	-1.19407	1.22448	-3.35876
H	-1.54229	2.40298	-2.11659
H	-0.75269	2.89861	-3.59441

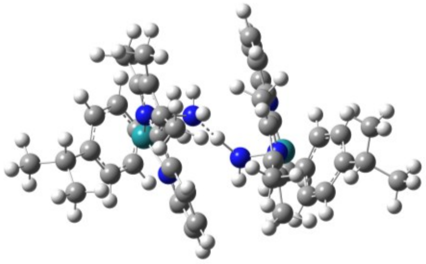

[1][**4-III**]$^{2+}$

E = -2226.140941 (a.u.)

Charge = 2 Multiplicity = 1

Ru	-2.88833	-0.99129	-0.07222
N	-2.07348	-0.52694	1.75133
N	-2.3637	0.90842	-0.36213
N	-1.35285	-1.87215	-0.36921
H	-1.46676	-2.51392	-1.17201
H	0.14651	-1.22415	0.5226
C	-1.74596	0.78255	1.93304
C	-1.9138	1.58219	0.76447
C	-4.49722	-2.29062	0.78737
H	-4.37487	-2.82896	1.72933
C	-4.12793	-2.93269	-0.42156
C	-4.18224	-2.14719	-1.60065
H	-3.8269	-2.55213	-2.55049
C	-5.13542	-1.00898	0.81022
H	-5.46993	-0.61234	1.76948
C	-5.24369	-0.26268	-0.35635
C	-1.22967	1.19885	3.17474
H	-0.96761	2.24481	3.32443
C	-1.59785	2.90169	0.42111
C	-4.65389	-0.82421	-1.53993
H	-4.65845	-0.2339	-2.45515
C	-1.87767	-1.43032	2.7213
H	-2.13077	-2.46444	2.4821
C	-2.32035	1.76358	-1.4202
C	-1.86065	3.01282	-0.96908
C	-3.63914	-4.33945	-0.4398
H	-3.23445	-4.63756	0.53614
H	-4.49314	-4.99772	-0.66985
H	-2.88112	-4.49273	-1.21972

C	-1.36393	-1.07608	3.95651
H	-1.21668	-1.83948	4.72116
C	-1.04329	0.26847	4.18147
H	-0.63688	0.5844	5.14511
C	-5.85151	1.11188	-0.43012
H	-5.23581	1.67381	-1.1524
C	-7.2635	0.99683	-1.0112
H	-7.92138	0.43333	-0.33066
H	-7.69334	1.99993	-1.15279
H	-7.26145	0.48606	-1.98631
C	-5.8401	1.8586	0.89412
H	-4.82493	1.91205	1.31667
H	-6.19993	2.88708	0.74318
H	-6.50097	1.3844	1.63667
Ru	2.75409	-1.01505	0.01705
N	1.83073	0.09084	-1.48445
N	3.05853	0.91624	0.64581
N	0.93095	-0.73471	1.00315
H	0.9485	-1.05338	1.97248
H	0.70498	0.26098	1.02595
C	1.96305	1.4461	-1.3982
C	2.61364	1.90049	-0.21078
C	2.81011	-3.00477	-0.9256
H	2.06605	-3.44422	-1.59205
C	2.68481	-3.17205	0.47114
C	3.64672	-2.53653	1.32283
H	3.54953	-2.61837	2.40685
C	3.83498	-2.16895	-1.46128
H	3.82778	-1.96691	-2.53416
C	4.79402	-1.53054	-0.64023
C	1.45589	2.26053	-2.43274
H	1.58157	3.34038	-2.37581
C	2.81066	3.1681	0.37957
C	4.66329	-1.73276	0.76837
H	5.33153	-1.18944	1.43895
C	1.17572	-0.4613	-2.51637
H	1.07937	-1.54856	-2.50168
C	3.50582	1.50677	1.77225
C	3.37811	2.91175	1.64665
C	1.55776	-3.95563	1.06059
H	0.71541	-4.03478	0.36009
H	1.90532	-4.97429	1.29775
H	1.20475	-3.50087	1.99734
C	0.65138	0.28951	-3.55337

H	0.13244	-0.20538	-4.37497
C	0.81259	1.68049	-3.50942
H	0.42653	2.3078	-4.31636
C	5.88769	-0.6364	-1.17485
H	6.03947	0.14647	-0.41241
C	7.18672	-1.43328	-1.29524
H	7.08327	-2.23859	-2.04076
H	8.0136	-0.77952	-1.61362
H	7.46828	-1.894	-0.33566
C	5.52985	0.04884	-2.48645
H	4.58031	0.60098	-2.41241
H	6.31796	0.76638	-2.76016
H	5.44406	-0.67273	-3.31448
C	-2.67935	1.38882	-2.81275
H	-2.50073	0.32096	-2.9985
H	-3.73525	1.609	-3.04381
H	-2.07246	1.96264	-3.52682
C	-1.67396	4.23428	-1.80681
H	-0.75246	4.7739	-1.53859
H	-1.61394	3.98458	-2.87606
H	-2.50865	4.94717	-1.68877
C	-1.06059	3.97247	1.30968
H	-1.70456	4.13884	2.18815
H	-0.05492	3.72397	1.68889
H	-0.98183	4.92687	0.77169
C	4.02181	0.75796	2.95102
H	3.55349	-0.23374	3.0292
H	5.11503	0.60912	2.91313
H	3.80982	1.30956	3.87925
C	3.79492	3.90962	2.67828
H	3.52419	3.57968	3.6945
H	4.88605	4.08133	2.68299
H	3.31712	4.88689	2.51392
C	2.47216	4.50259	-0.19874
H	3.05638	4.72244	-1.10822
H	1.40833	4.57772	-0.47718
H	2.67768	5.30734	0.52146

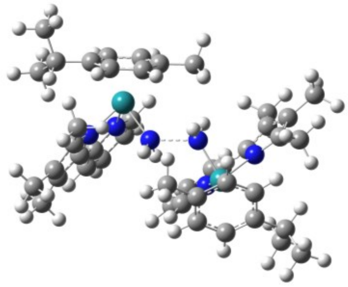

[1][**4-IV**]$^{2+}$
E = -2226.113470 (a.u.)
Charge = 2 Multiplicity = 1

Ru	-2.65556	-0.85051	-0.49802
N	-2.05787	-0.34251	1.41844
N	-3.50126	1.01304	-0.24892
N	-1.11131	0.16223	-1.1406
H	-0.80344	0.04733	-2.10849
H	-1.01526	1.13917	-0.85196
C	-2.33569	0.9322	1.81584
C	-3.06793	1.69963	0.86291
C	-2.41617	-3.06767	-0.39915
H	-1.67836	-3.62352	0.18259
C	-2.07441	-2.66237	-1.70875
C	-3.02778	-1.88625	-2.43558
H	-2.77936	-1.50108	-3.42669
C	-3.67905	-2.7445	0.17808
H	-3.86042	-3.04647	1.211
C	-4.61488	-1.96482	-0.51966
C	-1.91053	1.37483	3.0845
H	-2.14395	2.39034	3.40014
C	-3.45328	3.05161	0.77348
C	-4.24139	-1.51155	-1.83873
H	-4.88675	-0.81326	-2.36716
C	-1.36993	-1.16915	2.22545
H	-1.17113	-2.17045	1.84061
C	-4.22694	1.86484	-1.00968
C	-4.17457	3.15553	-0.43297
C	-0.75235	-3.00155	-2.3113
H	-0.01752	-3.27155	-1.54031
H	-0.87421	-3.86485	-2.98562
H	-0.36179	-2.16869	-2.91387
C	-0.93751	-0.78918	3.48114
H	-0.38787	-1.49668	4.10221
C	-1.22393	0.51224	3.91572
H	-0.90513	0.84607	4.90476

C	-5.94511	-1.57215	0.07491
H	-6.21256	-0.6083	-0.38195
C	-7.00281	-2.59492	-0.34034
H	-6.77699	-3.58566	0.08614
H	-7.99561	-2.28596	0.0214
H	-7.05615	-2.69898	-1.43503
C	-5.90222	-1.37667	1.58381
H	-5.11385	-0.66451	1.87418
H	-6.86617	-0.97851	1.93445
H	-5.72798	-2.32348	2.11857
Ru	2.47762	0.80881	-0.52695
N	2.47925	0.7124	1.55356
N	3.27812	-1.06223	-0.19233
N	0.80257	-0.28433	-0.37509
H	0.80093	-1.18486	-0.85654
H	0.37888	-0.40381	0.54731
C	2.97429	-0.43882	2.09215
C	3.37407	-1.42223	1.13463
C	2.52258	3.02946	-0.68987
H	2.15061	3.75742	0.0341
C	1.66129	2.56449	-1.69715
C	2.15091	1.54317	-2.57947
H	1.4806	1.10334	-3.32075
C	3.85754	2.53401	-0.55749
H	4.46362	2.87937	0.28092
C	4.35525	1.56224	-1.44399
C	3.0579	-0.56998	3.49359
H	3.45661	-1.48784	3.92207
C	3.8483	-2.74566	1.23484
C	3.46519	1.06843	-2.45978
H	3.79909	0.26122	-3.10669
C	2.05024	1.70249	2.34909
H	1.63397	2.57884	1.85123
C	3.66619	-2.11535	-0.94331
C	4.02496	-3.18645	-0.09309
C	0.27722	3.09712	-1.84048
H	-0.18552	3.29495	-0.86331
H	0.29876	4.05009	-2.39368
H	-0.33119	2.38974	-2.41269
C	2.13444	1.63757	3.72812
H	1.78823	2.47677	4.33207
C	2.65336	0.473	4.30579
H	2.73041	0.38123	5.39182
C	5.74801	0.9918	-1.3333

H	5.67758	-0.05494	-1.66853
C	6.67024	1.73465	-2.30043
H	6.76577	2.79472	-2.01519
H	7.67575	1.28644	-2.28973
H	6.29144	1.69265	-3.33348
C	6.29498	0.99256	0.08629
H	5.60445	0.49131	0.78209
H	7.25596	0.45747	0.11593
H	6.47718	2.01412	0.45543
C	3.7062	-2.13302	-2.43853
H	4.43073	-1.42026	-2.85211
H	4.00997	-3.12611	-2.78013
H	2.72248	-1.91106	-2.86877
C	4.51633	-4.52922	-0.54849
H	3.78193	-5.02898	-1.1914
H	5.44937	-4.44645	-1.11882
H	4.70754	-5.18405	0.30669
C	4.10411	-3.52604	2.4877
H	4.8652	-3.0452	3.1137
H	3.19502	-3.62507	3.09282
H	4.45859	-4.53286	2.2494
C	-5.09869	1.47845	-2.1575
H	-5.93454	0.85369	-1.79073
H	-5.55785	2.38102	-2.57
H	-4.56648	0.96729	-2.96459
C	-4.85399	4.3741	-0.98581
H	-4.47863	4.62384	-1.98516
H	-5.93761	4.22753	-1.06916
H	-4.68574	5.24198	-0.34139
C	-3.16543	4.14421	1.75656
H	-3.60748	3.93118	2.73684
H	-2.08676	4.27682	1.90315
H	-3.57563	5.09553	1.40656

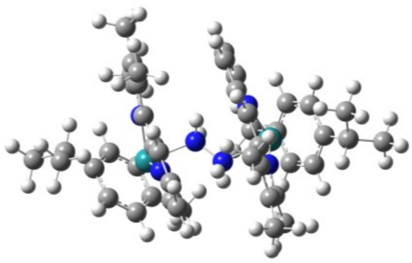

^{1}[**4-V**]$^{2+}$

E = -2226.207593 (a.u.)

Charge = 2 Multiplicity = 1

Ru	0.95785	0.10939	-0.51601
N	0.0354	1.85836	0.07545
N	-1.01379	-0.36444	-0.68126
N	0.94035	0.79609	-2.2985
H	1.67747	0.61992	-2.98052
H	0.19226	1.38113	-2.67165
C	-1.31493	1.78922	0.26143
C	-1.89332	0.55071	-0.14083
C	2.73943	0.44271	0.76411
H	3.11234	1.41361	1.09575
C	3.22426	-0.10058	-0.44547
C	2.63811	-1.32485	-0.88505
H	2.91885	-1.74362	-1.85403
C	1.79734	-0.25344	1.58727
H	1.46558	0.21856	2.5127
C	1.24275	-1.4645	1.16924
C	-1.99928	2.90894	0.77748
H	-3.07348	2.84888	0.94524
C	-3.20837	0.05042	-0.23107
C	1.64835	-1.96284	-0.11698
H	1.18945	-2.87606	-0.49564
C	0.70137	2.99617	0.31966
H	1.77157	2.98702	0.10742
C	-1.72047	-1.41815	-1.13645
C	-3.09314	-1.20473	-0.86681
C	4.25141	0.60155	-1.2665
H	4.28623	1.67292	-1.0284
H	5.24293	0.16955	-1.05376
H	4.05774	0.473	-2.34069
C	0.07581	4.12876	0.80893
H	0.65726	5.03258	0.99444
C	-1.30248	4.07022	1.05353
H	-1.82971	4.94048	1.45195
C	0.20208	-2.22647	1.94872
H	-0.50348	-2.62084	1.19825
C	0.86912	-3.41954	2.63624
H	1.59972	-3.08045	3.38801
H	0.11154	-4.03268	3.1476
H	1.39533	-4.06259	1.91416
C	-0.58006	-1.37123	2.93311
H	-1.02842	-0.49579	2.43883
H	-1.39446	-1.96453	3.37471
H	0.05368	-1.01565	3.76107
C	-4.46275	0.71605	0.23026

H	-4.43334	0.9528	1.30668
H	-4.6488	1.66498	-0.30042
H	-5.33683	0.07136	0.06187
C	-4.1889	-2.15933	-1.21385
H	-4.16662	-3.06703	-0.58579
H	-5.18018	-1.70078	-1.08638
H	-4.11702	-2.49941	-2.26039
C	-1.12361	-2.58631	-1.83878
H	-0.16913	-2.32022	-2.31411
H	-0.93964	-3.43704	-1.15987
H	-1.8062	-2.94999	-2.62191
Ru	1.61752	3.80832	-3.94789
N	-0.42474	3.76257	-3.65345
N	1.25525	5.64234	-3.14523
N	1.8072	3.07947	-2.19234
H	2.54137	2.42328	-1.92787
H	1.18315	3.30346	-1.41674
C	-1.0051	4.94667	-3.30156
C	-0.07463	5.99308	-3.03792
C	1.54483	2.44168	-5.69457
H	0.77529	1.69088	-5.8838
C	2.69351	2.07524	-4.96001
C	3.625	3.11253	-4.65671
H	4.48744	2.89856	-4.02148
C	1.37074	3.75902	-6.22754
H	0.4626	3.97733	-6.79049
C	2.29552	4.76797	-5.95232
C	-2.40923	5.02352	-3.19711
H	-2.87519	5.97238	-2.93594
C	-0.17594	7.31839	-2.56771
C	3.40748	4.4264	-5.10729
H	4.11475	5.20714	-4.82773
C	-1.17112	2.66415	-3.83836
H	-0.6322	1.74586	-4.07658
C	2.00585	6.68469	-2.73653
C	1.15288	7.75493	-2.37666
C	2.89702	0.68539	-4.46097
H	1.94614	0.13879	-4.40819
H	3.56568	0.14637	-5.15187
H	3.37354	0.68428	-3.47059
C	-2.55007	2.68025	-3.7302
H	-3.11772	1.76291	-3.89034
C	-3.17525	3.89413	-3.41621
H	-4.26351	3.94947	-3.33452

C	2.15224	6.19184	-6.42482
H	2.49547	6.82058	-5.58612
C	3.10117	6.42386	-7.60243
H	2.81237	5.8042	-8.46644
H	3.06769	7.47951	-7.91203
H	4.14201	6.17999	-7.33974
C	0.72383	6.58881	-6.76285
H	0.04021	6.38701	-5.92399
H	0.68027	7.66572	-6.98338
H	0.34959	6.05604	-7.6515
C	-1.42589	8.09231	-2.30689
H	-2.05855	8.17123	-3.20639
H	-2.04515	7.62855	-1.52065
H	-1.19553	9.11584	-1.97933
C	1.61717	9.08688	-1.88395
H	2.09444	9.68381	-2.68067
H	0.78377	9.6839	-1.48622
H	2.36249	8.98604	-1.07748
C	3.49146	6.66575	-2.65468
H	3.87126	5.64041	-2.5442
H	3.9681	7.11059	-3.54543
H	3.83316	7.25242	-1.78834

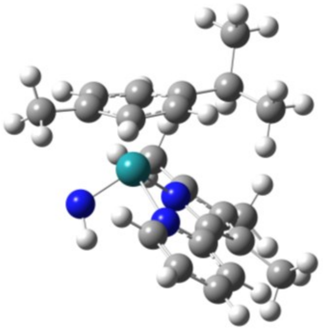

[1][**4-II**]$^{+}$

E = -1112.401107 (a.u.)

Charge = 1 Multiplicity = 1

Ru	0.95785	0.10939	-0.51601
N	0.0354	1.85836	0.07545
N	-1.01379	-0.36444	-0.68126
N	0.94035	0.79609	-2.2985
H	0.19226	1.38113	-2.67165
C	-1.31493	1.78922	0.26143
C	-1.89332	0.55071	-0.14083
C	2.73943	0.44271	0.76411
H	3.11234	1.41361	1.09575

C	3.22426	-0.10058	-0.44547
C	2.63811	-1.32485	-0.88505
H	2.91885	-1.74362	-1.85403
C	1.79734	-0.25344	1.58727
H	1.46558	0.21856	2.5127
C	1.24275	-1.4645	1.16924
C	-1.99928	2.90894	0.77748
H	-3.07348	2.84888	0.94524
C	-3.20837	0.05042	-0.23107
C	1.64835	-1.96284	-0.11698
H	1.18945	-2.87606	-0.49564
C	0.70137	2.99617	0.31966
H	1.77157	2.98702	0.10742
C	-1.72047	-1.41815	-1.13645
C	-3.09314	-1.20473	-0.86681
C	4.25141	0.60155	-1.2665
H	4.28623	1.67292	-1.0284
H	5.24293	0.16955	-1.05376
H	4.05774	0.473	-2.34069
C	0.07581	4.12876	0.80893
H	0.65726	5.03258	0.99444
C	-1.30248	4.07022	1.05353
H	-1.82971	4.94048	1.45195
C	0.20208	-2.22647	1.94872
H	-0.50348	-2.62084	1.19825
C	0.86912	-3.41954	2.63624
H	1.59972	-3.08045	3.38801
H	0.11154	-4.03268	3.1476
H	1.39533	-4.06259	1.91416
C	-0.58006	-1.37123	2.93311
H	-1.02842	-0.49579	2.43883
H	-1.39446	-1.96453	3.37471
H	0.05368	-1.01565	3.76107
C	-4.46275	0.71605	0.23026
H	-4.43334	0.9528	1.30668
H	-4.6488	1.66498	-0.30042
H	-5.33683	0.07136	0.06187
C	-4.1889	-2.15933	-1.21385
H	-4.16662	-3.06703	-0.58579
H	-5.18018	-1.70078	-1.08638
H	-4.11702	-2.49941	-2.26039
C	-1.12361	-2.58631	-1.83878
H	-0.16913	-2.32022	-2.31411
H	-0.93964	-3.43704	-1.15987

H	-1.8062	-2.94999	-2.62191

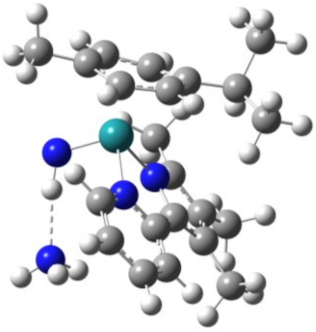

[1][**4-VI**]$^+$

E = -1168.861770 (a.u.)

Charge = 1 Multiplicity = 1

Ru	0.96388	0.16705	-0.60282
N	0.10926	1.90498	0.00948
N	-0.97241	-0.28124	-0.77499
N	1.42621	0.97024	-2.11975
H	0.91671	1.86513	-2.26004
C	-1.23859	1.85431	0.2222
C	-1.83835	0.62927	-0.19096
C	2.65459	0.27279	0.85437
H	3.05739	1.22489	1.20596
C	3.24297	-0.3462	-0.28067
C	2.60922	-1.51019	-0.77156
H	2.94881	-1.97528	-1.69893
C	1.64752	-0.37512	1.64058
H	1.30969	0.11154	2.55612
C	1.06298	-1.55108	1.18879
C	-1.89094	2.97272	0.77389
H	-2.96313	2.92943	0.95931
C	-3.14846	0.13478	-0.26035
C	1.48905	-2.02902	-0.09655
H	0.98385	-2.89796	-0.51858
C	0.80757	3.02103	0.27049
H	1.87594	2.98843	0.04766
C	-1.69846	-1.32827	-1.24143
C	-3.05399	-1.11077	-0.9317
C	4.41338	0.2548	-0.97493
H	4.48359	1.33299	-0.77999
H	5.33055	-0.22173	-0.59108
H	4.36888	0.07637	-2.05725
C	0.20992	4.15156	0.79714
H	0.8115	5.03917	0.99573

C	-1.1647	4.1141	1.06212
H	-1.66739	4.98566	1.48798
C	-0.04969	-2.27332	1.90111
H	-0.76472	-2.56898	1.11441
C	0.50502	-3.55266	2.53278
H	1.23589	-3.31298	3.32141
H	-0.31206	-4.13308	2.98737
H	1.00268	-4.19209	1.78758
C	-0.7869	-1.41411	2.91592
H	-1.15613	-0.48223	2.46091
H	-1.65327	-1.96523	3.31048
H	-0.14508	-1.15109	3.77182
C	-4.38845	0.79131	0.24618
H	-4.31703	1.02571	1.32071
H	-4.5953	1.7405	-0.2761
H	-5.26411	0.14234	0.10831
C	-4.16747	-2.04336	-1.27869
H	-4.18277	-2.93242	-0.62446
H	-5.14872	-1.55579	-1.18821
H	-4.07572	-2.41373	-2.31266
C	-1.12841	-2.469	-2.00517
H	-0.14988	-2.21227	-2.43359
H	-1.00797	-3.37271	-1.38329
H	-1.80116	-2.74391	-2.83221
N	1.98521	2.00689	-4.09252
H	2.14997	1.29406	-4.77423
H	2.86017	2.36349	-3.76497
H	1.45703	2.7483	-4.50646

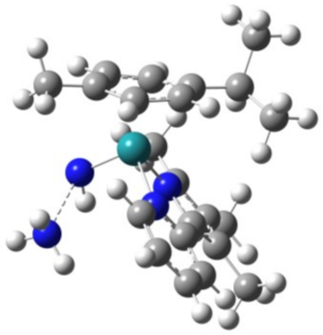

^{1}[**4-VII**]$^{+}$

E = -1168.852392 (a.u.)

Charge = 1 Multiplicity = 1

Ru	0.20568	0.56952	-0.40091
N	-1.49982	0.06416	0.62695
N	-0.21426	-1.24634	-1.16537

N	-0.67127	1.33231	-1.80131
H	-1.38333	0.70127	-2.20725
C	-2.03454	-1.15559	0.33939
C	-1.30664	-1.89249	-0.64626
C	1.10968	1.85975	1.25056
H	0.54208	2.4058	2.00705
C	1.42871	2.51229	0.03507
C	2.09606	1.73311	-0.94464
H	2.2869	2.15373	-1.93448
C	1.60793	0.5589	1.5726
H	1.3855	0.14373	2.5569
C	2.25671	-0.20821	0.61089
C	-3.21285	-1.57617	0.97637
H	-3.62304	-2.55754	0.73369
C	-1.43655	-3.14597	-1.25863
C	2.41145	0.38092	-0.69518
H	2.85815	-0.217	-1.4924
C	-2.11284	0.88239	1.49739
H	-1.64894	1.85588	1.65996
C	0.34962	-2.04066	-2.09636
C	-0.37884	-3.23735	-2.18146
C	1.02824	3.92127	-0.23474
H	0.25007	4.25358	0.46513
H	1.90223	4.58232	-0.11825
H	0.66243	4.0328	-1.26554
C	-3.27817	0.52227	2.14896
H	-3.74219	1.2184	2.84863
C	-3.83319	-0.73607	1.88305
H	-4.75305	-1.05049	2.38169
C	2.74573	-1.61321	0.84762
H	2.6436	-2.13545	-0.11876
C	4.23298	-1.57325	1.2056
H	4.38902	-1.05144	2.16345
H	4.62691	-2.59628	1.30502
H	4.82209	-1.05443	0.43384
C	1.93288	-2.37259	1.88599
H	0.85983	-2.36629	1.63888
H	2.26628	-3.42028	1.92627
H	2.05828	-1.95015	2.8954
N	-2.83959	2.30416	-1.26938
H	-3.63309	1.78285	-0.89652
H	-2.60103	3.0223	-0.58711
H	-3.1497	2.77956	-2.11592
C	1.59708	-1.5968	-2.88277

H	1.36427	-0.72568	-3.45884
H	2.3883	-1.37109	-2.19873
H	1.90539	-2.38523	-3.53715
C	-0.06731	-4.41714	-3.12095
H	-0.11193	-4.08522	-4.13719
H	0.91281	-4.79135	-2.91067
H	-0.78637	-5.19456	-2.96776
C	-2.525	-4.19643	-0.9698
H	-2.50216	-4.45776	0.06755
H	-3.48489	-3.79204	-1.21469
H	-2.3442	-5.06936	-1.56158

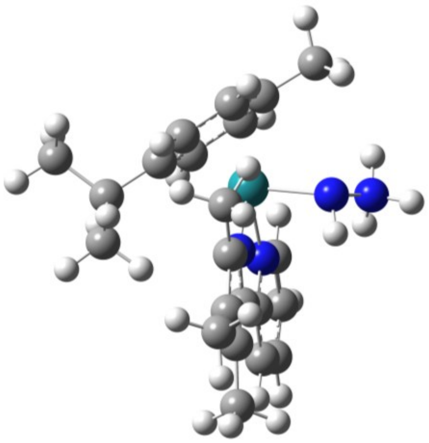

^{1}**[4-VIII]**$^{+}$

E = -1168.905680 (a.u.)

Charge = 1 Multiplicity = 1

Ru	0.89283	0.04172	-0.42361
N	0.07686	1.65599	0.52679
N	-1.08132	-0.28056	-0.69475
N	0.94334	0.74269	-2.09465
H	-0.00308	0.99398	-2.42734
C	-1.28463	1.64325	0.66481
C	-1.92307	0.55136	0.01491
C	2.74687	0.02579	0.88606
H	3.19123	0.90279	1.36134
C	3.1873	-0.34183	-0.40705
C	2.52442	-1.44238	-1.00833
H	2.75865	-1.72047	-2.03851
C	1.83047	-0.77265	1.63981
H	1.58416	-0.4625	2.65648
C	1.16572	-1.8374	1.0443
C	-1.91545	2.68801	1.3723
H	-2.99676	2.67016	1.49866
C	-3.25991	0.13535	-0.17034

C	1.47857	-2.10593	-0.33684
H	0.94433	-2.9085	-0.84621
C	0.80277	2.67893	1.00731
H	1.87779	2.63703	0.82804
C	-1.82946	-1.18882	-1.34984
C	-3.19442	-0.973	-1.03893
C	4.23962	0.42143	-1.13553
H	4.4177	1.39817	-0.66597
H	5.18511	-0.14419	-1.1265
H	3.95259	0.57212	-2.18637
C	0.22973	3.73614	1.68715
H	0.85784	4.54629	2.05897
C	-1.15941	3.72583	1.8808
H	-1.64653	4.53912	2.42415
C	0.12049	-2.67578	1.73292
H	-0.63467	-2.90509	0.96209
C	0.7463	-3.99726	2.18328
H	1.52132	-3.82213	2.94667
H	-0.0224	-4.65285	2.62029
H	1.21201	-4.53318	1.34196
C	-0.57387	-1.95642	2.87892
H	-0.9884	-0.98975	2.55259
H	-1.40283	-2.57192	3.25873
H	0.11176	-1.77196	3.72117
N	0.96224	2.17975	-2.16286
H	0.20497	2.75093	-1.78818
H	1.82279	2.46491	-1.69838
H	1.05916	2.39447	-3.15419
C	-1.27899	-2.2212	-2.26902
H	-0.28564	-1.93254	-2.63923
H	-1.1889	-3.20866	-1.78393
H	-1.94268	-2.355	-3.13694
C	-4.32318	-1.79287	-1.57375
H	-4.38587	-1.74023	-2.67436
H	-4.21594	-2.85997	-1.31536
H	-5.29148	-1.45565	-1.17681
C	-4.48683	0.74747	0.42028
H	-4.43504	0.79592	1.52029
H	-4.65048	1.77798	0.06216
H	-5.38252	0.16616	0.15984

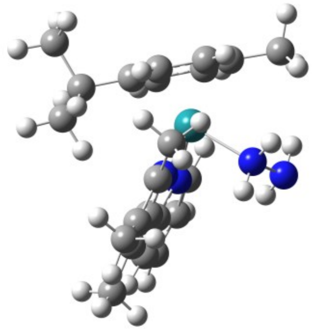

[1][**4-IX**]$^{+}$

E = -1170.084262 (a.u.)

Charge = 1 Multiplicity = 1

Ru	0.87909	-0.01928	-0.38504
N	-0.19673	1.6774	0.15501
N	-1.05533	-0.69185	-0.44538
N	0.41628	0.54415	-2.37578
H	-0.58989	0.40524	-2.50962
C	-1.54747	1.51706	0.28158
C	-2.01928	0.21102	-0.04782
C	2.94683	0.57868	0.14851
H	3.40847	1.56336	0.0552
C	3.02034	-0.33368	-0.92653
C	2.36	-1.59686	-0.79582
H	2.36627	-2.30167	-1.62937
C	2.20182	0.25204	1.31917
H	2.07999	1.02163	2.08386
C	1.55249	-0.99386	1.4774
C	-2.33951	2.60828	0.69635
H	-3.41566	2.47971	0.7992
C	-3.29677	-0.38275	-0.15243
C	1.64221	-1.90277	0.37874
H	1.08471	-2.83954	0.43021
C	0.37095	2.86524	0.41546
H	1.45306	2.92181	0.2856
C	-1.6737	-1.82864	-0.82541
C	-3.07023	-1.68359	-0.64889
C	3.75139	0.00608	-2.18341
H	3.89809	1.09024	-2.28485
H	4.74435	-0.47182	-2.16224
H	3.22506	-0.37534	-3.0704
C	-0.35811	3.96731	0.82463
H	0.14995	4.91111	1.02615
C	-1.74451	3.82572	0.96814
H	-2.35618	4.67155	1.29156
C	0.75732	-1.37366	2.70415

H	-0.08136	-1.99192	2.34186
C	1.61851	-2.24104	3.62233
H	2.48378	-1.67175	3.99923
H	1.03343	-2.58438	4.48968
H	2.00029	-3.13005	3.09681
C	0.1741	-0.18058	3.44794
H	-0.4167	0.46552	2.78085
H	-0.48833	-0.52975	4.25425
H	0.95949	0.43467	3.9148
N	0.72532	1.84529	-2.87319
H	0.25882	2.5125	-2.25659
H	1.7238	1.99201	-2.71599
H	0.85008	-0.12537	-3.01677
C	-0.96898	-3.02243	-1.36782
H	-0.02609	-2.74083	-1.85756
H	-0.73264	-3.76528	-0.58606
H	-1.59944	-3.53481	-2.11012
C	-4.08078	-2.7434	-0.94771
H	-4.00975	-3.09696	-1.99052
H	-3.95437	-3.63219	-0.30579
H	-5.1069	-2.37775	-0.79777
C	-4.61585	0.23681	0.17378
H	-4.67667	0.55359	1.22832
H	-4.81873	1.13104	-0.4393
H	-5.43818	-0.47117	-0.0016

Reference:

[1] C. Ducloiset, P. Jouin, E. Paredes, R. Guillot, M. Sircoglou, M. Orio, W. Leibl, A. Aukauloo, *Eur. J. Inorg. Chem*. **2015**, *32*, 5405-5410.

[2] J. J. Klappa, A. E. Rich, K. McNeill, *Org. Lett.* **2002**, *4*, 435-437.

[3] G. W. Watt, J. D. Chrisp, *Anal. Chem.* **1952**, *24*, 2006-2008.

[4] A. Najafian, T. R. Cundari, *J. Phys. Chem. A.* **2019**, *123*, 7973-7982.

[5] Q. Chen, J. Liang, L. Yue, Y. Luo, Q. Liu, N. Li, A. A. Alshehri, T. Li, H. Guo, X. Sun, *Chem. Commun.* **2022**, *58*, 5091-5904.

[6] D. Zhu, L. Zhang, R. E. Ruther, R. J. Hamers, *Nat. Mater.* **2013**, *12*, 836-841.

[7] M. J. Frisch, G. W. Trucks, H. B. Schlegel, *Gaussian Inc*. **2016**.

[8] C. Adamo, V. Barone, *J. Chem. Phys*. **1999**, *110*, 6158-6170.

[9] S. Grimme, S. Ehrlich, L. Goerigk, *J. Comput. Chem*. **2011**, *32*, 1456-1465.

[10] F. Weigend, R. Ahlrichs, *Phys. Chem. Chem. Phys*. **2005**, *7*, 3297-3305.

[11] F. Weigend, *Phys. Chem. Chem. Phys.***2006**, *8*, 1057-1065.

[12] A. V. Marenich, C. J. Cramer, D. G. Truhlar, *J. Phys. Chem. B*. **2009**, *113*, 6378-6396.

[13] J. Shen, M. Wang, P. Zhang, J. Jiang, L. Sun, *Chem. Commun.* **2017**, *53*, 4374-4377.
